# Influence of apolipoprotein E genotype on the proteomic profile in cerebral microdialysis after human severe traumatic brain injury: a prospective observational study

**DOI:** 10.1093/braincomms/fcaf096

**Published:** 2025-03-03

**Authors:** Caroline Lindblad, Andrea Klang, David Bark, Cristina Bellotti, Anders Hånell, Per Enblad, Anders Lewén, Elham Rostami

**Affiliations:** Department of Medical Sciences, Section of Neurosurgery, Uppsala University, Uppsala SE-751 85, Sweden; Department of Clinical Neuroscience, Karolinska Institutet, Stockholm SE-171 64, Sweden; Department of Medical Sciences, Section of Neurosurgery, Uppsala University, Uppsala SE-751 85, Sweden; Department of Rehabilitation Medicine, Uppsala University Hospital, Uppsala SE-751 85, Sweden; Department of Medical Sciences, Section of Neurosurgery, Uppsala University, Uppsala SE-751 85, Sweden; Department of Neuroscience, Karolinska Institutet, Stockholm SE-171 65, Sweden; Department of Medical Sciences, Section of Neurosurgery, Uppsala University, Uppsala SE-751 85, Sweden; Department of Medical Sciences, Section of Neurosurgery, Uppsala University, Uppsala SE-751 85, Sweden; Department of Medical Sciences, Section of Neurosurgery, Uppsala University, Uppsala SE-751 85, Sweden; Department of Medical Sciences, Section of Neurosurgery, Uppsala University, Uppsala SE-751 85, Sweden; Department of Neuroscience, Karolinska Institutet, Stockholm SE-171 65, Sweden

**Keywords:** traumatic brain injury, cerebral microdialysis, apolipoprotein E, neuroinflammation, proteomics

## Abstract

Patient-tailored treatment, also known as precision-medicine, has been emphasized as a prioritized area in traumatic brain injury research. In fact, pre-injury patient genetic factors alone account for almost 26% of outcome prediction variance following traumatic brain injury. Among implicated genetic variants single-nucleotide polymorphism in apolipoprotein E has been linked to worse prognosis following traumatic brain injury, but the underlying mechanism is still unknown. We hypothesized that apolipoprotein E genotype would affect the levels of pathophysiology-driving structural, or inflammatory, proteins in cerebral microdialysate following severe traumatic brain injury. We conducted a prospective observational study of patients with severe traumatic brain injury treated with invasive neuromonitoring including cerebral microdialysis at Uppsala University Hospital. All patients were characterized regarding apolipoprotein E genotype. Utilizing fluid- and plate-based antibody arrays, we quantified 101 proteins (of which 89 were eligible for analysis) in cerebral microdialysate at 1 day and 3 days following trauma. Statistical analysis included clustering techniques, as well as uni- and multi-variate linear mixed modelling. In total, 26 patients were included, and all relevant genotypes of apolipoprotein E were represented in the data. Among all proteins tested, 41 proteins showed a time-dependent expression level. There was a weak clustering tendency in the data, and not primarily to genotype, either depicted through t-distributed stochastic neighbour embedding or hierarchical clustering. Using linear mixed models, two proteins [the inflammatory protein CD300 molecule like family member f (CLM-1) and the neurotrophic protein glial-derived neurotrophic factor family receptor α1] were found to have protein levels concomitantly dependent upon time and genotype, albeit this effect was not seen following multiple testing corrections. Apart from amyloid-β-40 (Aβ) and Microtubule-associated protein tau, neither Aβ peptide levels nor the Aβ42/40 ratio were seen related to time from trauma or apolipoprotein E genotype. This is the first study in clinical severe traumatic brain injury examining the influence of apolipoprotein E genotype on microdialysate protein expression. Protein levels in cerebral microdialysate following trauma are seen to be strongly dependent on time from trauma, corroborating previous work on protein expression longitudinally following traumatic brain injury. We also identified protein expression level alterations dependent on apolipoprotein E genotype, which might indicate that apolipoprotein E affects ongoing pathophysiology in the injured brain at the proteomic level.

## Teaser

Lindblad *et al.* utilized antibody arrays to quantify 101 proteins in cerebral microdialysate longitudinally following human severe traumatic brain injury among 26 patients with different apolipoprotein E genotypes. They delineate time, age and apolipoprotein E genotype-dependency for brain extracellular protein levels, and present two novel candidates for future pathophysiological studies.

## Graphical Abstract

**Graphical Abstract:**
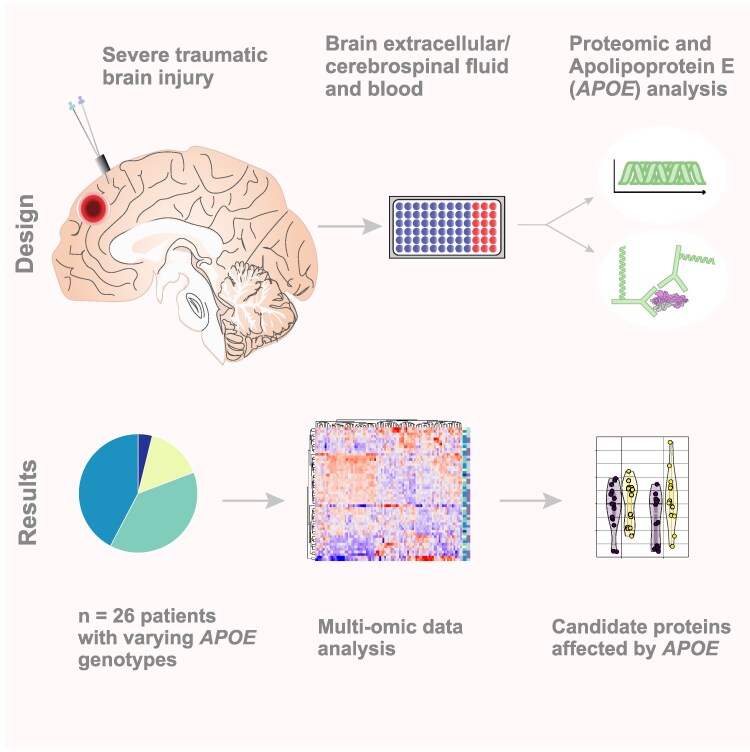

## Introduction

Precision-medicine has been emphasized as a prioritized area in traumatic brain injury (TBI) research.^1,2^ Among putative factors eligible for personalized treatment considerations, pre-injury patient characteristics are likely to be important. In fact, patient genetic set-up alone accounted for almost 26% of outcome prediction variance utilizing Glasgow Outcome Score Extended (GOSE)^3^ 6 months post-TBI.^4^ Several diverging genetic variants have been implicated,^5^ but genetic variations in the apolipoprotein E (*APOE*) stands out as particularly well-studied.^6^ In the CNS, APOE is a predominantly astrocytic protein encoded by the *APOE* gene located at chromosome 19^7,8^ with two commonly occurring single-nucleotide polymorphisms (SNPs) at position 112 and 158 causing the replacement of cysteine with arginine.^8^ In total, there are three different *APOE* alleles—*APOE* ɛ2, ɛ3 and ɛ4, thus enabling six different genotypes.^8,9^ In the absence of head injury, the ɛ4 allele has been attributed to exhibit a dose-dependent increased risk for Alzheimer's disease.^10^ Interestingly, in small TBI cohorts of varying injury severity, *APOE* ɛ4 carriers have been observed to have a worse prognosis.^11,12^ These findings have later been corroborated in meta-analyses with robustly incremented patient cohorts.^6,13^

The exact mechanism by which *APOE* ɛ4 is deleterious is still unknown. Recently, *APOE* ɛ4 carriers without head injury were shown to exhibit blood–brain barrier (BBB) breakdown and activation of the matrix metalloproteinase 9 pathway.^14^  *APOE* ɛ4 has also been associated with heightened microglial activation and other neuroinflammatory mediators^15^ in a cohort of non-TBI subjects.^15,16^ We have previously investigated BBB disruption, and neuroinflammatory pathways including matrix metalloproteinase 9 in CSF following severe TBI,^17^ but without finding any relationship to *APOE* ɛ4. One opportunity onwards to understand *APOE*-associated pathophysiology is to use cerebral microdialysis,^18^ utilized to focally monitor metabolism in the cerebral extracellular space,^19^ while it also allows for studies of e.g. neuroinflammation and directed proteomic investigations.^20^ With this in mind, we hypothesized that *APOE* ɛ4 allele homozygosity or heterozygosity would influence focal levels of proteins in brain extracellular fluid (i.e. microdialysate) following severe human TBI. We did not assume that protein levels would be related to *APOE* ɛ4 in a dose-dependent manner. We, therefore, aimed to undertake antibody-based proteomic profiling of microdialysate in a human severe TBI cohort, where the *APOE* genotype was known.

## Materials and methods

This was a prospective, observational study undertaken at the neurocritical care unit (NCCU) at Uppsala University Hospital (Uppsala, Sweden) between December 2014 and September 2020. Written informed consent was obtained from next-of-kin. The study was undertaken in accordance with the Declaration of Helsinki and Swedish law. Ethical approval was granted by the Uppsala County branch of the Swedish Ethical Review Authority (#2010/138, #2010/138/1, #2010/379, #2015/224 and #2020-05462).

### Patient inclusion, management and sample size considerations

All patients included were 15 years or older and had sustained a severe TBI, which necessitated multi-modal invasive intracranial monitoring including cerebral microdialysis. A TBI was defined as ‘an alteration in brain function, or other evidence of brain pathology, caused by an external force’.^21^ A severe TBI was defined clinically as a Glasgow Coma Scale (GCS) ≤ 8,^22,23^ which although imperfect is congruent with recent international multi-centre collaborative efforts.^2^ In addition, patients with a GCS > 8 but with neuroradiological signs on computerized tomography evocative of impending deterioration or increased intracranial pressure such as compressed basal cisterns, mid-line shift >5 mm and/or an un-evacuated mass lesion, or smaller lesions concurrently co-existing^24^ were also deemed as a severe TBI. These findings were routinely assessed by the neurosurgeon together with the neuroradiologist on-call in our centre. These radiological findings are also well in-line with formal CT classification systems,^25^ of which the original Marshall classification system^26^ was specifically developed to find patients with a mis-match between their clinical status and neuroradiological imagery. Sample size estimation was decided utilizing currently available literature, where several studies have investigated neuroinflammatory proteins in brain extracellular fluid from severe TBI patients.^27-29^ Two studies were observational^28,29^ and included *n* = 10 patients, whereas one^27^ was a follow-up study to a phase II randomized controlled trial^30^ that included *n* = 20 patients in total. In total, we therefore sought to recruit >20 patients. Patient enrolment was dependent on senior author presence, thus leading to non-consecutive enrolment of subjects into the study. Data on patients not enrolled are not available.

Uppsala University Hospital has a catchment area of 1.5–2 million citizens. Patients are either admitted directly from our hospital or transferred from regional hospitals across the catchment area, where initial patient stabilization and resuscitation are undertaken before transfer to our department. Patient management is undertaken in accordance with the Brain Trauma Foundation Guidelines,^31^ meaning that intracranial pressure (ICP) is maintained <20 mmHg, while cerebral perfusion pressure is maintained at >60 mmHg. In addition, a standardized clinical management protocol focused on secondary insult prevention has been implemented and shown to improve long-term outcome.^32^ In brief, patients were mechanically ventilated and received propofol and morphine for sedation and analgesia. We aimed for PaO_2_ ≥ 12 kPa. Patients with unstable ICP were initially hyperventilated, aiming at a PaCO_2_ range of 4.0–4.5 kPa. Patients were gradually normo-ventilated as soon as ICP permitted. For all patients, systolic blood pressure was targeted to be >100 mmHg. All patients obtained neuromonitoring equipment comprising an intracranial pressure monitor entailing either an external ventricular drain (HanniSet, Xtrans, Smith Medical GmbH), or an intra-parenchymatous monitor (Codman ICP Micro-Sensor, Codman & Shurtleff). The cerebral microdialysis catheter was inserted concurrently with the ICP device via the same burr-hole or craniotomy as the ICP device. The implantation site of choice was the non-dominant (usually right) frontal lobe, ca. 1–2 cm anteriorly to the coronal suture or else the same as the location of the craniotomy. A needle-based corticotomy was done, and the catheter was inserted at ∼20–30 mm depth. A 71 high cut-off brain microdialysis catheter with a membrane length of 10 mm and a membrane cut-off of 100 kDa (M Dialysis AB, Stockholm, Sweden) was used. We utilized perfusion fluid CNS (M Dialysis AB) containing NaCl (147 mmol/L), KCl (2.7 mmol/L, CaCl_2_ (1.2 mmol/L) and MgCl_2_ (0.85 mmol/L). Across the study period, we initiated perfusion fluid supplementation with human serum albumin^33^ followed by 3% dextran, where the latter has been shown to improve inflammatory marker recovery *in vitro*.^34^ A standard perfusion rate of 0.3 μL/min was achieved using a 106 Microdialysis pump (M Dialysis AB) and analysed utilizing either the ISCUSflex microdialysis analyzer or the CMA 600 analyzer (M Dialysis AB). As a routine, the microdialysis monitoring should continue for at least 5 days. When the patient was stable and the neurointensive care was completed, the patient was either moved to a step-down unit or discharged to the referring hospital. Following discharge, patients were followed-up at 6–12 months through either a structured questionnaire or a telephone interview documenting the GOSE.^3^

### Sample collection, data acquisition and raw-data processing

Patients were sampled from whole-blood via an arterial line and from brain extracellular fluid (microdialysate) upon the first and third day following trauma. DNA was extracted from 200 μL whole-blood by using the Qiagen QIAamp (Qiagen) blood mini kit. DNA quantification was performed utilizing the Invitrogen Qubit 1X dsDNA HS Assay kit (Thermo Fisher Scientific) and the DNA quality was assessed through the Agilent Tapestation genomic DNA screen tape (Agilent). All samples were of high quality (DNA Integrity Number > 7). PCR amplification (background read and allelic discrimination) was run with Mastermix TaqPath ProAmp (Thermo Scientific). PCR reactions were run in triplicates in 384-well plates in 5 μL reactions with 5 ng of DNA. Amplification, background read and allelic discrimination were performed on Applied Biosystem HT 7900 PCR-machine (Thermo Fisher Scientific). For SNP discrimination, the human TaqMan SNP Genotyping Assay (Thermo Fisher Scientific) with the catalogue numbers C_3084793_20 (rs429358) and C_904973_10 (rs7412) were used. Raw-data processing was undertaken via Thermo Fisher Scientific's analytical platform Thermo Fisher Connect™. *APOE* allele status was determined by combining the output of the two assays so that *APOE* ɛ2 allele denotes rs7412T/rs429358T (amino acids: cysteine 112/cysteine 158); *APOE* ɛ3 allele denotes rs7412 C/rs429358T (amino acids cysteine 112/arginine 158) and *APOE* ɛ4 allele denotes rs7412 C/rs429358 C (amino acids arginine 112/arginine158).^8,9^ All genotyping steps were undertaken via the Karolinska Institutet Bioinformatics and Expression Analysis Core Facility.

Microdialysis samples were analysed through a proximity extension assay (Olink, Uppsala, Sweden), quantifying *n* = 92 proteins implicated in the CNS or its disorders. All Olink assays were undertaken by the company. For one protein [Interleukin (IL)-12], the protein was matched with two different UniProt IDs,^35^ as the protein IL-12 occurs both as IL-12α and IL-12β. All protein levels are reported as ‘normalized protein expression’, an arbitrary unit on log_2_ scale derived from the company. Quality control in conjunction with the analysis was conducted per assay and sample through *n* = 4 internal controls. To fulfil quality control criteria, the sample concentration of the internal control should deviate ≤0.3 from the median control concentration. All samples passed quality control, but several samples exhibited specific protein levels below the limit of detection. For each instance where the protein concentration was below the lower limit of detection, the protein concentration value was encoded as a missing value.

In addition, *n* = 9 proteins not available for proximity extension assay were quantified in microdialysate through multi-array technology, i.e. a combination between a protein, antibody-based array and electrochemiluminescence via Meso Scale Discovery Inc. (Maryland, USA), who also undertook all sample processing. We utilized the pre-designed V-plex Ab peptide panel for the neurodegenerative proteins Amyloid-β (Aβ)-38, -40 and -42 (Meso Scale Discovery Inc.). Aβ-38, -40 and -42 are all fragments from the Aβ precursor protein and are thus reported together for tissue enrichment descriptions. We also customized a U-plex panel for quantifications of brain-derived neurotrophic factor and human nerve growth factor (β-NGF); as well as the neuroinflammatory proteins IL-1β, -6, -8 and vascular endothelial growth factor (VEGF) (Meso Scale Discovery Inc.). For meso scale analyses, all protein concentrations (reported in picograms per millilitre) were derived through backfit calculation using a standard curve. Values below or above the fitted curve range were considered as missing values, while values within the curve fit but outside of the optimal detection range were included.

**Figure 1:**
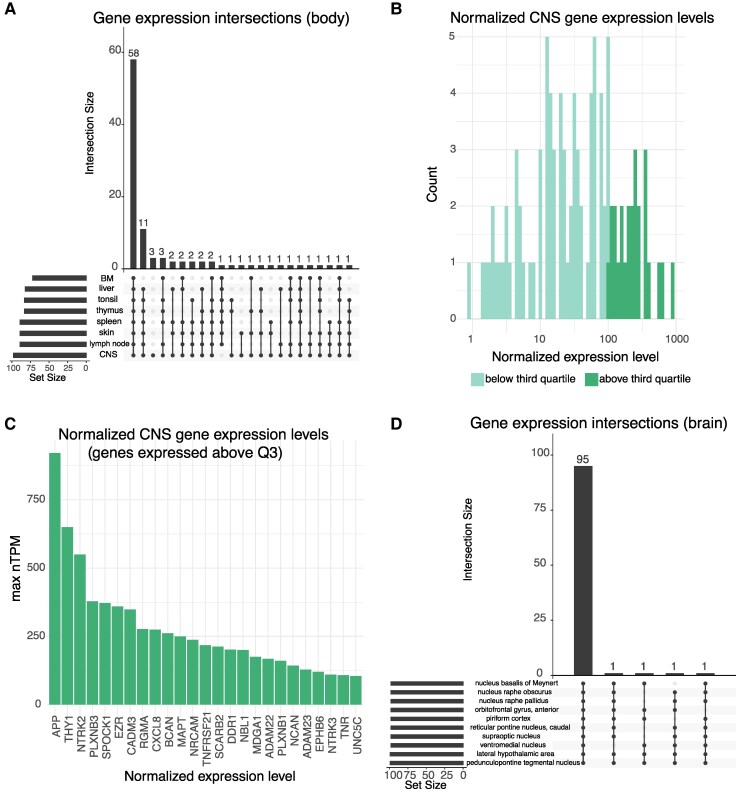
**Baseline characteristics of the investigated proteins.** In total, *n* = 101 proteins were investigated. At baseline, the genes coding for these proteins have a distinct enrichment in both the CNS and lymphoid tissues across the body (**A**). Among CNS-enriched genes in our data (**B**), *n* = 25 had the 25% highest expression levels (**C**). These proteins are, under homeostatic conditions expressed at the gene level in a multitude of CNS sub-regions (**D**). BM, bone marrow; CNS, central nervous system. Full protein names can be found in Supplementary Table 1.

A comprehensive overview on the analysed proteins, including Uniprot and Ensembl ID is found in Supplementary Table 1. Normal tissue expression and regional relative tissue enrichment have been robustly described through the Human Protein Atlas (HPA) effort.^36-39^ We used two public RNA sequencing datasets from the HPA (version 23) with Ensembl annotations (version 109), available through https://v23.proteinatlas.org/about/download (files: rna_tissue_consensus.tsv; rna_brain_hpa.tsv) to determine relative tissue enrichment (reported as the normalized tissue expression levels)^40^ of the proteins that we measured (Fig. 1A). Utilizing this, we set a cut-off of 0.1^40^ to exclude non-enriched proteins. Notably, several of the proteins assessed were enriched across both the CNS and the immune system under homeostatic conditions. Among the protein-encoding genes that we studied, we report HPA-derived CNS expression levels (Fig. 1B), as well as the upper quartile of CNS-enriched genes (Fig. 1C), and their regional CNS expression under homeostasis (Fig. 1D).

### Statistical analysis

All statistical operations were conducted utilizing R version 4.3.1,^41^ through the interface Rstudio version 2023.09.0 + 463. Continuous variables are presented as mean (SD) if normally distributed or else median (inter-quartile range). Categorical variables are presented as count (percentage). Unless stated otherwise, a *P* value ≤ 0.05 was considered significant. Across all operations, the tidyverse package^42^ was used. In addition, for data curation the janitor package^43^ was used. For graphical illustrations, the additional packages UpSetR,^44-46^ ggforce,^47^ RColorBrewer,^48^ cowplot,^49^ CompexHeatmap^50,51^ and circlize^52^ were used. Other relevant packages are cited in adjunct to specific operations where they were applied.

Missing data, most notably for the various proteins measured, were visualized graphically and computationally utilizing the visdat^53^ and naniar packages^54^ in addition to base R. In total, *n* = 12 proteins had missing values (for either of the two sampling time points) exceeding 70% of the total number of observations (Supplementary Table 2). This pertained to the proteins: NGF-β (measured through the proximity extension assay platform), CLEC10A, BMP4, NEP, WFIKKN1, FCRL2, IL-5R-α, LAT, CHD3, CDH6, NAAA and PRTG (full protein names described in Supplementary Table 1). These proteins were deemed too uncertain to impute and were therefore excluded from downstream analysis. For the remaining proteins, we imputed data utilizing the mice package.^55^ We undertook this analysis utilizing the pipeline suggested by one of the mice package creators.^56,57^ Hence, we imputed *n* = 20 datasets with *n* = 10 iterations. The method defaulted to by the mice package was predictive mean matching for the proteins. Imputations were conducted utilizing age and sex data on the study subjects as well as protein data. For downstream analyses, calculations and operations were conducted for each imputed dataset whereafter results were pooled.

Dimensionality reduction analysis was performed using t-distributed stochastic neighbouring (tSNE) algorithms, through the package Rtsne.^58^ Clustering analyses were undertaken utilizing the packages cluster,^59^ factoextra,^60^ NbClust,^61^ clValid^62^ and mclust.^63^ Clustering tendency of the data was evaluated utilizing the Hopkins statistic. The number of clusters and the clustering technique was determined by comparing *n* = 21 various clustering algorithms and defining the optimal number of clusters as the most common output from the different clustering algorithms. Clustering methods and number of clusters were evaluated utilizing three different clustering scores (Connectivity, Dunn and Silhouette).

For inferential analyses, we employed linear mixed models through the nlme package.^64^ Primarily, we were interested in protein levels as the dependent variable and *APOE* genotype (dichotomized into ɛ4 carrier or not) as the independent predictor variable, utilizing time from trauma and patient age as tentative covariates. We utilized uni- and multi-variable regression modelling. For multi-variable models, we employed a top–down modelling strategy, including all tentative predictors from univariate analysis including interaction terms, after which non-significant variables were sequentially removed so that the final model retained only significant variables. Across all models, we allowed random intercept but abstained from random slopes models, as we only had *n* = 2 time points for observations. We also abstained from defining an autocorrelation structure, as we utilized time as covariate. We also abstained from including distance from the microdialysis probe to the lesion as covariate, primarily because of the small sample size and risk for data overfitting. Analysis results are presented both in their unadjusted form (denoted *p*_unadjusted_), and following Benjamini–Hochberg (i.e. the false discovery rate) multiple testing correction (denoted *p*_adjusted_). Regression assumptions, such as residual variance homogeneity and residual normality, were modelled graphically. In addition, since we did not use any autocorrelation structure in the data, we also modelled the partial autocorrelation of model residuals, the latter through the forecast package.^65^ If the regression criteria seemed to be fulfilled graphically for the majority of proteins across the majority of imputations, the regression model was accepted to enable comparison between proteins.

## Results

### Patient and neuromonitoring characterization

In total, 26 patients were included and underwent invasive neuromonitoring as well as *APOE* genotyping (Table 1). Most patients were middle-aged men, who were unconscious at admission with a mixed injury pattern. Mortality in this cohort was notably lower than compared with reference literature.^66^ In total, 11 patients (42%, including subjects lost to follow-up) had an unfavourable long-term outcome at 6 (5–7) months follow-up.

**Table 1:** Patient characteristics

Variable	TBI cohort (*n* = 26)	Unit/metric
Age	45 (18) [15–81] Missing: 0 (0)	Years Mean (SD) [min, max]
Sex (Male)	19 (73) Missing: 0 (0)	Count (%)
Pupils	Bilaterally normal: 22 (85) Unilaterally unresponsive: 1 (3.8) Bilaterally unresponsive: 3 (12) Missing: 0 (0)	Count (%)
Glasgow coma scale (admission)	7 (7–9) Missing: 15 (58)	Arbitrary unit (median, interquartile range [IQR])
Glasgow Coma Scale Motor Score (admission)	5 (5–5) Missing: 0 (0)	Arbitrary unit (median, IQR)
Anticoagulant therapy	2 (7.7) Missing: 1 (3.8)	Count (%)
Injury mechanism	Assault: 1 (3.8) Sport/recreational: 1 (3.8) Pedestrian accident: 2 (7.7) Bike accident: 3 (12) Other: 3 (12) Unspecified vehicle accident: 7 (27) Fall: 9 (35) Missing: 0 (0)	
Dominant injury pattern	Fracture of the calvarium: 1 (3.8) Traumatic contusion: 5 (19) Acute subdural haematoma: 7 (27) Mixed injuries: 13 (50) Missing: 0 (0)	Count (%)
Pentobarbital coma throughout NCCU stay	2 (7.7) Missing: 2 (7.7)	Count (%)
Days in respirator	13 (9–18) Missing: 0 (0)	Days (median, IQR)
Months to follow-up	6 (5–7) Missing: 6 (23)	Months (median, IQR)
Glasgow Outcome Scale Extended	1. Dead: 1 (3.8) 2. Vegetative state: 1 (3.8) 3. Lower severe disability: 5 (19) 4. Upper severe disability: 4 (15) 5. Lower moderate disability: 2 (7.7) 6. Upper moderate disability: 1 (3.8) 7. Lower good recovery: 5 (19) 8. Upper good recovery: 4 (15) Missing: 3 (12)	Count (%)
Glasgow Outcome Scale Extended (dichotomized)	Favourable: 12 (46) Unfavourable: 11 (42) Missing: 3 (12)	Count (%)

Patient demographics summarized across the study cohort. Continuous data are depicted as mean (SD) if normally distributed and otherwise median (inter-quartile range). Categorical data are described as count (percentage). NCCU, neurocritical care unit; TBI, traumatic brain injury.

**Figure 2:**
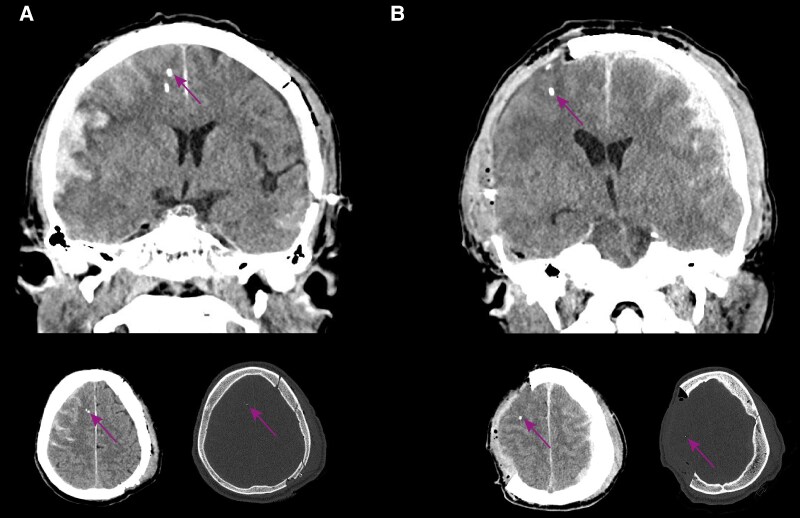
**Representative examples of localization of cerebral microdialysis.** The cerebral microdialysis probe has a gold-tip coating,^20^ making it discernible on computerized tomography imaging. In **A**, a conventional non-dominant right frontal lobe placement of the probe is seen whereas in **B**, probe placement has been placed in adjunct to a hemicraniectomy. The arrows highlight the microdialysis tip. The other hyperattenuating tip is the parenchymal intracranial pressure monitor.

Characteristics of the implanted cerebral microdialysis are shown in Fig. 2 and Table 2. Most commonly, the microdialysis probe was positioned in the non-dominant (usually right) frontal lobe (Table 2 and Fig. 2A). If the patient underwent a craniotomy in the same séance as the neuromonitoring equipment was implanted, the microdialysis was occasionally pragmatically put in the vicinity of the craniotomy (Fig. 2B).

**Table 2:** Cerebral microdialysis

Variable	TBI cohort (*n* = 26)	Unit/metric
Anatomical region	Frontal lobe: 24 (92) Missing: 2 (7.7)	Count (%)
Hemisphere	Right: 20 (77) Left: 4 (15) Missing: 2 (7.7)	Count (%)
Distance from cortical surface	18 (12) Missing: 2 (7.7)	Millimetre (mean, SD)
Distance from closest lesion	36 (22) Missing: 2 (7.7)	Millimetre (mean, SD)
Distance from largest lesion	42 (25) Missing: 2 (7.7)	Millimetre (mean, SD)
Type of largest lesion used for distance measurement	Extra-axial bleeding (non-evacuated): 4 (15) Extra-axial bleeding (evacuated): 5 (19) Intra-axial bleeding (non-evacuated): 10 (38) Intra-axial bleeding (evacuated): 1 (3.8) Mixed bleeding (non-evacuated): 1 (3.8) Hemicraniectomy: 2 (7.7) Skull base fracture: 1 (3.8) Missing: 2 (7.7)	Count (%)

Characteristics of the implanted cerebral microdialysis, reported in accordance with currently available consensus literature.^67^ TBI, traumatic brain injury.

The microdialysis probe was implanted 18 (±12) mm from the cortical surface, and notably 36 (±22) mm from the closest injury lesion and 42 (±25) mm from the largest detectable lesion.

### Protein levels in brain extracellular fluid follow a temporally distinct pattern and are dependent on patient age

**Figure 3:**
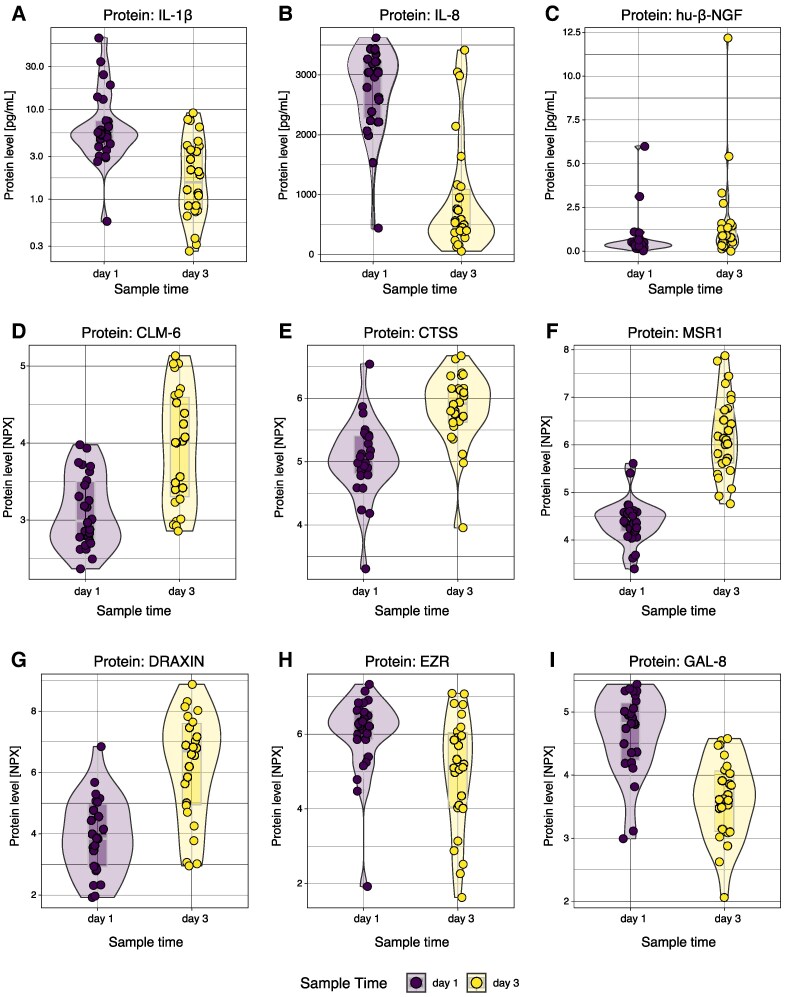
**Protein expression in brain extracellular fluid is dynamic following severe TBI.** Protein analysis was undertaken at the first and third day following trauma. Notably, several proteins of varying origin and functions display a longitudinal trajectory. In total, *n* = 41 proteins demonstrated a temporal trajectory (*n* = 34 proteins retained significance following multiple testing correction), of which a representative subset is demonstrated above (**A**-**I**). Here, one dot represents one study subject at one time point. Calculations were done utilizing a univariate linear mixed model with time as independent variable and patient identity as the random effect. Numerical results (*t*-statistics and *P*-values) are available in Supplementary Table 3. Here, no adjustment to APOE status is undertaken. All protein concentrations (except for IL-1β, IL-8 and β-NGF, where concentration is in picograms per millilitre) are provided in normalized protein expression (NPX) which is the arbitrary unit on the log_2_ scale utilized by the proximity extension assay company. Full protein names are displayed in Supplementary Table 1. d, day; NPX, normalized protein expression; TBI, traumatic brain injury.

We quantified 89 proteins and 1 protein ratio (Aβ42/40) longitudinally in cerebral microdialysate following TBI. Several proteins displayed a time-dependent trajectory upon univariate analysis without consideration of *APOE* status (*n* = 41, with *p*_unadjusted_ ≤ 0.05; *n* = 34 with *p*_adjusted_ ≤ 0.05; Supplementary Table 3). Normalized longitudinal trajectories are depicted for all significant proteins in Supplementary Fig. 1, while absolute protein concentrations for nine significant proteins are depicted in Fig. 3. Notably, as no control subjects were included in the study, the impact of the TBI on the Day 1 protein level is difficult to assess. The neuroinflammatory proteins IL-1β and IL-8 were seen to express higher values at Day 1 than at Day 3 following trauma (Fig. 3A and B). In accordance, it is well-known that innate immune proteins such as IL-β (Fig. 3A) increase early following trauma,^68^ reflecting microglia-mediated caspase-driven cleavage of pro-IL-1β into its mature form.^69^ IL-8 (Fig. 3B) has also been shown to be incremented closely following clinical TBI.^70^ Interestingly, both IL-1β and IL-8 have been linked to the production of β-NGF (Fig. 3C),^70,71^ and it has been suggested that astrocytes drive NGF production.^72^ Here, β-NGF demonstrated higher values at Day 3 than at Day 1, possibly speaking in favour of a delayed production. Other proteins, normally enriched in lymphoid regions of the body (CLM-6, CTSS and MSR1)^39,73^ (Fig. 3D–F), demonstrated higher values at Day 3 compared with Day 1 following trauma. CNS-enriched proteins^39,73^ (DRAXIN and EZR; Fig. 3G-H) demonstrated opposite trends. While DRAXIN (Fig. 3G) demonstrated higher values at Day 3 compared with Day 1 following trauma, EZR (Fig. 3H) showed the opposite trend. Other proteins, not necessarily primarily enriched in the immune system or CNS^39,73^ such as GAL-8 (Fig. 3I) also demonstrated time-dependent protein concentration alterations. Among all proteins investigated, *n* = 4 were dependent on patient age in univariate analysis, and comprised the proteins EDA2R, HAGH, MANF and SPOCK1 (Supplementary Table 4). None of these age-dependent proteins had protein levels dependent on time from trauma.

### Protein clustering depends on time from trauma, but not on *APOE* genotype

**Figure 4:**
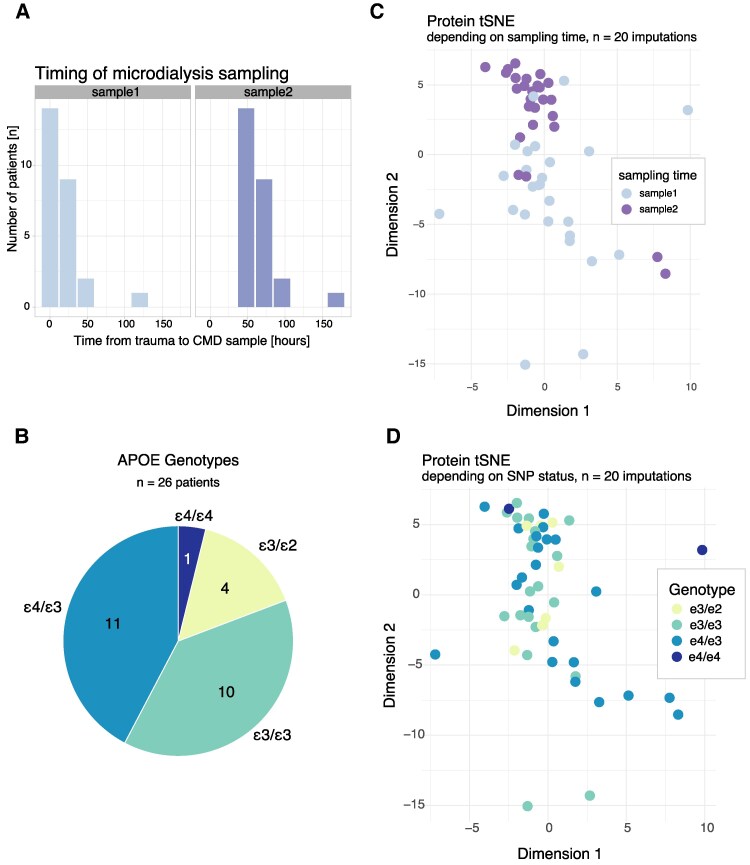
**tSNE analysis of cerebral microdialysate proteins.** Cerebral microdialysate sampling was undertaken for *n* = 89 proteins at 1 day and 3 days following trauma (**A**) among patients with varying *APOE* genotypes (**B**). Cluster analysis utilizing tSNE was undertaken with regard to sampling time, where a clear clustering tendency was shown (**C**), as well as genotype, where however no obvious clustering tendency was shown (**D**). For **C** and **D**, the pooled results of all *n* = 20 imputations are shown. *APOE*, apolipoprotein E; SNP, single-nucleotide polymorphism; tSNE, t-distributed stochastic neighbour embedding.

Cerebral microdialysis samples analysed for protein content at the first [0 (0–24) h] and third day [48 (48–72) h] following severe TBI are shown in Fig. 4A. All patients that underwent microdialysate sampling were genotyped for *APOE* SNP status (Fig. 4B). In large clinical datasets of patients, the ɛ3 allele is the most common, followed by firstly the ɛ4 and secondly the least common ɛ2 allele.^74^ This is partly mimicked in our data, where *n* = 14 patients (54%) express either ɛ3/ɛ3 or ɛ3/ɛ2. However, *n* = 12 patients (46%) expressed homozygosity or heterozygosity for ɛ4, which is probably higher than the general population. We next attempted clustering analysis utilizing tSNE to account for time and genotype (Fig. 4C and D). Whereas, patients’ protein content clearly clustered depending on time from trauma (Fig. 4C), there was no obvious clustering tendency depending on patient genotype (Fig. 4D).

**Figure 5:**
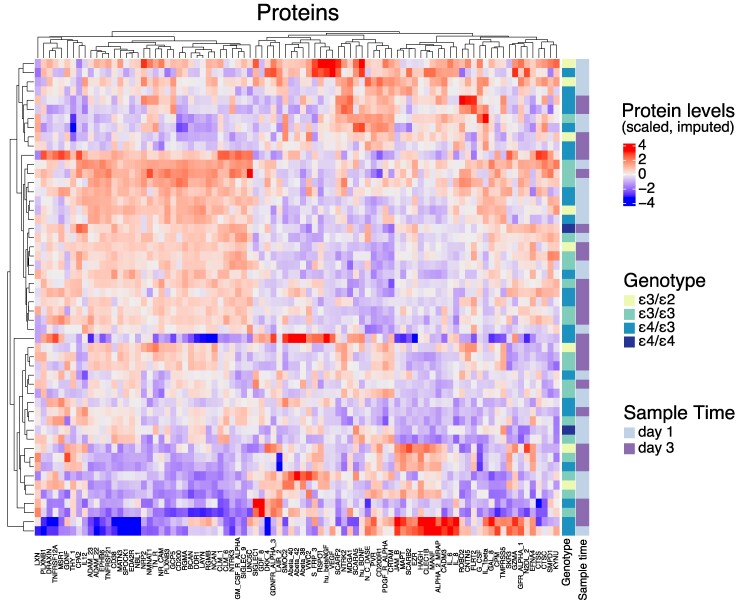
**Heatmap of cerebral microdialysate proteins in relation to genotype.** Cerebral microdialysis was undertaken on *n* = 89 proteins from *n* = 26 patients. Samples clustered with regard to protein content, but not obviously with relation to *APOE* genotype. d, day following trauma. Full protein names are displayed in Supplementary Table 1.

To further explore this, we also depicted all protein levels for all patients depending on time and *APOE* status utilizing a heatmap (Fig. 5). Here, a weak clustering between proteins is discerned, but without any obvious relationship to patient genotype.

### Two proteins have *APOE* SNP-dependent levels, following adjustment for time from trauma

**Figure 6:**
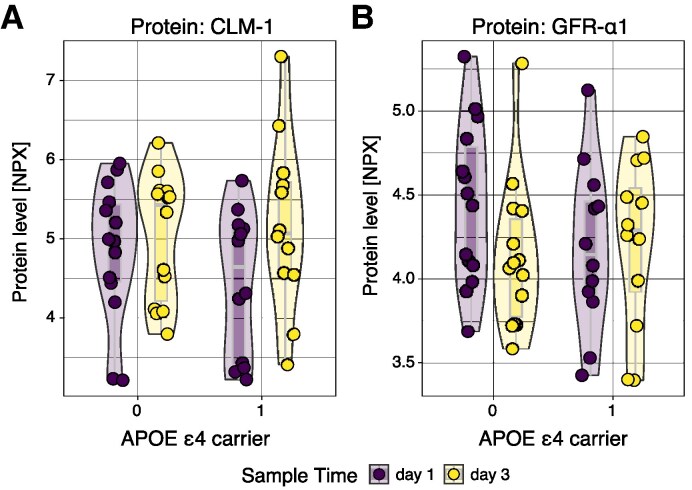
**Graphical output of interaction model of *APOE* ɛ4 carrier status and time for microdialysate protein level.** Levels of both the immune protein CLM-1 (CD300LF and CD300f) (**A**), and the GDNF receptor GFR-α1 (**B**), are seen to be concomitantly dependent on time from trauma and *APOE* ɛ4 carrier status. CLM-1 (**A**) is higher among non-*APOE* ɛ4 carriers at both Day 1 and Day 3 following trauma, although there is a general increase in CLM-1 levels over time. Conversely, GFR-α1 levels (**B**) decrease over time among patients without *APOE* ɛ4, whereas protein levels increase longitudinally among *APOE* ɛ4 carriers. Here, one dot represents one study subject at one time point. Calculations were done utilizing a multi-variable mixed model with *APOE* ɛ4 carrier and time as independent variables presented as an interaction effect and patient identity as the random effect. Numerical results (β coefficients and *P-*values) are available in Supplementary Table 5. Full protein names are displayed in Supplementary Table 1. *APOE*, apolipoprotein; d, day; GDNF, glial-derived neurotrophic factor.

We conducted regression analyses, modelling protein levels as dependent on the time elapsed since trauma and/or *APOE* ɛ4 carriership (homozygote or heterozygote). Upon interaction modelling, *APOE* ɛ4 carriership was a significant predictor of cerebral microdialysate levels of two proteins—the glial-derived neurotrophic factor (GDNF) family receptor α1 (GFRα1) and the protein CD300 molecule like family member f (CLM-1, also known as CD300f, CD300LF) (Fig. 6A-B). This was discerned as an interaction effect, for which the pooled *P*-values were *P* = 0.039 (CLM-1) and *P* = 0.020 (GFR-α1), respectively. CLM-1 levels were higher in non-*APOE* ɛ4 carriers at all time points, while GFR-α1 levels were only higher at the first day following trauma. For both, the interaction effect conferred increased protein levels over time, meaning that *APOE* ɛ4 carriership increased protein levels as time from trauma increased (Supplementary Table 5). Notably, CLM-1 is enriched in lymphoid organs^39,73^ and has been implicated as a protective factor in experimental (excitotoxic) brain injury.^75^ In contrast, GFR-α1 is the core receptor for GDNF, implicated in neurite outgrowth and branching.^76^ Similar results were found when conducting the same regression analysis but also adjusting for age. Notably, following multiple testing correction, no proteins retained significance.

### Inflammatory and neurodegenerative proteins exhibit time-dependent concentration levels following TBI, but do not seem to be related to *APOE* ɛ4 carrier status

*APOE* ɛ4 has been associated with inflammatory-mediated disease and BBB disruption as possible mechanisms prompting neurodegenerative disease.^10,14,15^ We assessed numerous inflammatory and BBB proteins, including the previously mentioned CLM-1, IL-1β and IL-8, but also IL-6 and VEGF. In contrast to IL-1β, *APOE* SNP status had a trend to predict IL-6 levels (*p*_unadjusted_ = 0.081). Upon multiple testing corrections, this effect was no longer significant. Protein levels of VEGF were not dependent on either time from trauma or *APOE* SNP status.

**Figure 7:**
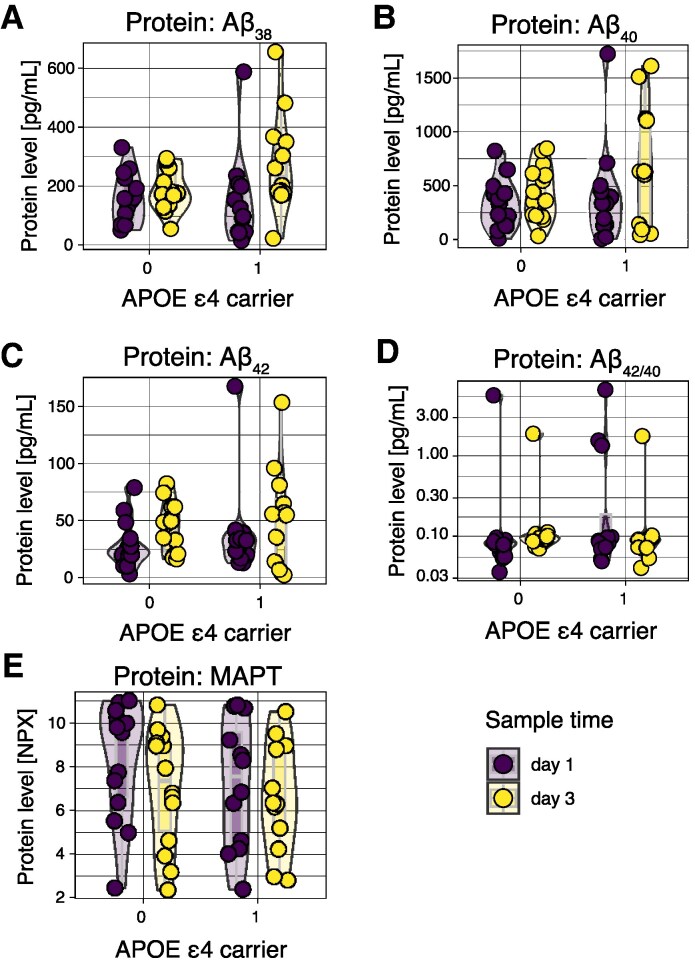
**Graphical output of regression model of *APOE* ɛ4 carrier status and time for neurodegenerative proteins assessed in microdialysate.** Levels of Aβ38 (**A**) seem to be independent of time from trauma and *APOE* ɛ4 carrier status. Levels of Aβ40 (**B**) seem time-dependent (*p*_unadjusted_ = 0.044, *p*_adjusted_ = NS), while Aβ42 (**C**) and the ratio of Aβ42/40 (**D**) do not exhibit any strong trends. Protein levels of MAPT (**E**) were dependent on time (*p*_unadjusted_ = 0.040, *p*_adjusted_ = 0.096), but not genotype. Here, one dot represents one study subject at one time point. Calculations were done utilizing a univariate linear mixed model with time as independent variable and patient identity as the random effect. Numerical results (*t*-statistics and *P* values) are available for significant proteins in Supplementary Table 3. Full protein names are displayed in Supplementary Table 1. *APOE*, apolipoprotein; d, day; Aβ, amyloid-β; MAPT, microtubule-associated protein tau.

We also assessed the neurodegenerative proteins microtubule-associated protein tau (MAPT) and the amyloid-β (Aβ) peptides Aβ38, Aβ40, Aβ42 and the ratio Aβ42/40, as the latter has been evocative of intracerebral deposition of amyloid plaques (Fig. 7).^77^ The time elapsed since trauma predicted the levels of MAPT (*p*_unadjusted_ = 0.032, *p*_adjusted_ = 0.077; Supplementary Table 3) and Aβ40 peptide (*p*_unadjusted_ = 0.044, *p*_adjusted_ = NS; Supplementary Table 3). However, none of the other neurodegenerative protein markers, including the Aβ42/40 ratio, exhibited a significant association. *APOE* ɛ4 homozygosity or heterozygosity was not a predictor for microdialysate levels of these neurodegenerative proteins, neither upon univariate analysis nor adjusted for time upon multi-variate analysis.

## Discussion

This is the first report on brain extracellular fluid proteomics in relation to *APOE* SNP in 26 human patients with severe TBI. Brain extracellular fluid retrieved utilizing microdialysis demonstrated temporally distinct patterns of protein expression, reflecting severe TBI pathophysiology in the injured brain. We also characterized all proteins regarding SNP in *APOE* ɛ4 carriership, the currently known strongest genetic risk factor for late onset Alzheimer's disease^78^ and an independent predictor of poor outcome following severe TBI.^6,13^ Notably, acute (1 day and 3 days) protein levels of MAPT and Aβ peptides did not seem to be related to *APOE* genotype. In contrast, for the inflammatory protein CLM-1 and the neurotrophic protein GFR-α1, we found that protein levels in adjunct to time from trauma were dependent on carriership of *APOE* ɛ4, however, neither protein retained significance following multiple testing correction. This possibly reflects how a patient's genetic background also affects disease-processes following TBI. Our findings could be utilized for continuous fluid biomarker efforts, while they also entail novel pathophysiological data, laying the foundation for future disease-modifying studies as well as therapeutic implications, discussed in detail below.

### Precision-based medicine warrants detailed pathophysiological portrayal in meticulously characterized clinical cohorts

Despite intense research efforts, treatment for severe TBI is directed to prevent secondary insult development rather than its underlying pathophysiology.^1,2^ In fact, some aspects of TBI pathophysiology are likely not actively monitored clinically. In line with this, the International Mission for Prognosis and Clinical Trial prognostic tool for assessment of functional neurologic outcome^79,80^ (via Glasgow Outcome Scale^81^) has an explained variance of ∼35%.^82^ This model contains exclusively pre-hospital and admission data, leading recent work to also include variables reflecting patient trajectories following admission.^83^ Even though this improves prediction to ∼52%, almost half of the variance in functional neurological outcome is still unaccounted for.^83^ We hypothesize that ongoing pathophysiology, such as neuroinflammation^69,84^ underlies at least some of this difference. The same holds true for pre-injury patient characteristics, such as specific genotypes, known to influence outcome.^4^

We therefore assessed *n* = 101 proteins in cerebral microdialysate following injury, of which *n* = 89 were eligible for analysis. We utilized two different platforms, one utilizing a proximity extension assay,^85,86^ and the other through a plate-based antibody-array coupled with electrochemiluminescence.^87^ Although it naturally would have been preferable to utilize one method, the current approach allowed us to profit from the highly multiplexed capability offered by proximity extension assays, while we could also assess proteins not yet reliably quantifiable utilizing this technique. This allowed us to examine a panel of structural neurological proteins, as well as inflammatory and neurodegenerative proteins. These proteins clustered with relation to time, and time was also an important predictor for the protein levels of 41 proteins in microdialysate. This mimics the sequential onset of neuroinflammation following TBI, known to occur in a strict time-dependent fashion.^88^ We can corroborate this in our data, by demonstrating the time-dependency of among else IL-1β and IL-8.^68,70^ Further, our results here also indicate that other structural (e.g. EZR and DRAXIN) as well as inflammatory proteins (e.g. CLM-6, CTSS and MSR1) follow a similarly stringent time-dependency. Jointly, this might indicate that the protein expression discerned is evocative of ongoing pathophysiology throughout the first 3 days following TBI. Importantly, our data shows that cerebral microdialysate detects these pathophysiological events. Onwards, this means that cerebral microdialysis might be utilized for monitoring of pathophysiology in the injured brain beyond the conventional detection of ischaemia. It would also be of interest to use microdialysis to monitor treatment response, as has been done both following metabolic and inflammatory modulation.^30,89^

### *APOE* ɛ4 homozygosity or heterozygosity is related to focal protein expression, but does not influence MAPT or amyloid-β levels acutely

In adjunct to microdialysate protein quantifications, we characterized all patients with regard to *APOE* ɛ4 carrier status. Upon regression modelling, levels of two proteins (CLM-1 and GFR-α1) seemed to be influenced by an interaction effect between time and *APOE* status. In contrast to our findings, previous work utilizing CSF proteomics in a larger patient cohort did not show any convincing relationship between *APOE* ɛ4 status and protein levels.^17^ The discrepancy likely reflects that the underlying study design and protein panels were different. Yet, as our results did not retain significance following multiple testing corrections, they need to be replicated and then externally validated in an autonomous dataset.

We observed altered levels of the proteins CLM-1 and GFRα1. CLM-1 levels were higher in non-APOE ɛ4 carriers at all time points, while GFR-α1 levels were only higher at the first day following trauma. For both, the interaction effect conferred increased protein levels over time, meaning that *APOE* ɛ4 carriership increased protein levels as time from trauma increased. CLM-1 is an immune-system protein that in the CNS is expressed by glial cells.^75,90^ Recent data indicates that inhibition of CLM-1 is neurotoxic *in vivo* following an experimental penetrating TBI.^91^ In our data, *APOE* ɛ4 carriers seemed to have somewhat lower levels of CLM-1 both at Day-1 and -3 following trauma. In contrast to this, the GFRα1 is the core receptor for the neurotrophic peptide GDNF.^92^ Originally, GDNF-GFRα1 was found to support survival of mid-brain dopaminergic neurons and was thus of interest in Parkinson's disease research,^93^ but has since been seen to be important also for neuronal maintenance and plasticity in homeostasis.^94,95^ GDNF rather than GFRα1 has been investigated in experimental TBI^96,97^ and showed some promise. In our data, it would seem as if patients without the *APOE* ɛ4 allele express higher GFRα1 levels earlier, which speculatively could be beneficial for neuronal survival. Taken together, we show that protein levels might alter in relation to patient genotype. Speculatively, genotype-dependent levels of immune cells and neurotrophic cues could affect acute lesion development following TBI, thus possibly accounting for prognostic differences seen among patients with *APOE* ɛ4 SNP.

We also investigated the neurodegenerative proteins MAPT, Aβ38, Aβ40, Aβ42 and the ratio of Aβ42/40, the latter as it has been associated with amyloid plaque deposition in the brain.^77^ We did not see any relationship between either MAPT or amyloid-β peptide levels and *APOE* ɛ4 carrier status. SNP of *APOE* is the currently known strongest genetic risk factor for late-onset Alzheimer's disease,^78^ linked to BBB dysfunction.^14^ Numerous reports^6,7,11,13^ have described a link between *APOE* ɛ4 and poor outcome following TBI. Interestingly, *APOE* ɛ4 homozygosity or heterozygosity has also been linked to deficient repair of the BBB also following TBI.^98^ As BBB disruption following TBI seems to be influenced by neuroinflammatory pathways,^98^ and *APOE* is synthesized by astrocytes and microglia, two innate neuroinflammatory cells,^9^ it is reasonable to believe that neuroinflammation is altered because of *APOE* genotype. We therefore specifically quantified the levels of neuroinflammatory proteins following human TBI. Among neuroinflammatory proteins, IL-1β is a core cytokine mediator of the TBI inflammatory response.^69^ The IL-1 signalling pathway prompts downstream inflammatory activation, e.g. of IL-6.^99^ IL-6 has also been shown to be increased in CSF following human TBI,^17^ and is of relevance for cell interactions *in vitro* in neuroinflammatory model systems.^100^ We did not see any robust relationship between IL-1β or IL-6 and *APOE* SNP status. Taken together, the *APOE* genotype is observed to be related to certain acute protein alterations (CLM-1 and GFR-α1), but not to acute changes in conventional neuroinflammatory or neurodegenerative proteins.

## Limitations

We acknowledge limitations with our study, most notably a small cohort size derived from a single centre. Study enrolment was non-consecutive, and we do not have access to data on patients not enrolled. Even though our cohort is similar to other TBI cohorts in the literature, we cannot exclude a systematic difference between patients enrolled and not-enrolled thus affecting external validity of our results. In addition, the limited cohort also likely yields the non-significant results upon multiple testing corrections, possibly reflecting a type II error. Yet, this cohort is amongst the most rigorously characterized in a severe TBI context, allowing hypothesis-generating work for future investigations in larger cohorts. Another limitation with our work is that cerebral microdialysis potentially is too focal a method to allow CNS-wide extrapolation,^20^ illustrated by earlier work that found limited overlap between protein quantifications in CSF, followed by cerebral microdialysis.^101^

## Conclusion

Cerebral microdialysis reflects ongoing temporally distinct severe TBI pathophysiology. Whereas acute and subacute protein levels of Aβ peptides do not seem to be related to *APOE* genotype, both the neurotrophic receptor GFRα1 and the inflammatory receptor CLM-1 exhibited protein levels dependent on *APOE* genotype. This possibly reflects how a patient's genetic background also affects acute disease-processes following TBI, which might have therapeutic implications, but warrants further contextualization and external validation in larger clinical cohorts.

## Supplementary Material

fcaf096_Supplementary_Data

## Data Availability

Data and/or analysed data are available from the corresponding author on reasonable request and will be sent in a format that adheres to current Swedish and European Union legislation regarding study participant anonymity. All code relevant for inferential analysis is available via https://github.com/carolinelindblad/apoE_TBI_20240804_CL (currently a private repository until publication).
