# Supplementary material for: Influence of apolipoprotein E genotype on the proteomic profile in cerebral microdialysis after human severe traumatic brain injury: a prospective observational study
Source: Brain Commun. 2025 Mar 3;7(2):fcaf096. doi: 10.1093/braincomms/fcaf096 (PMC11920868; doi:10.1093/braincomms/fcaf096)
Supplement: fcaf096_Supplementary_Data [file fcaf096_supplementary_data.pdf]

Supplementary Material

Influence of Apolipoprotein E Genotype on the Proteomic Profile in Cerebral Microdialysis after Human Severe Traumatic Brain Injury: A Prospective Observational Study

Caroline Lindblad[1, 2], Andrea Klang[1, 3], David Bark[1], Cristina Bellotti[4], Anders Hånell[1], Per Enblad[1], Anders Lewén[1], and Elham Rostami[1, 4]

Supplementary Table 1: List of all included proteins, included both through proximity extension assay (Olink) and Meso Scale Discovery.

UniProt ID	OlinkID	Protein abbreviation	Alternative protein abbreviation	EnsemblID (version109)	Full Protein Name
Q9HAN9	OID00287	NMNAT1		ENSG00000173614	nicotinamide nucleotide adenylyltransferase 1
O60462	OID00288	NRP2		ENSG00000118257	neuropilin 2
P10636	OID05024	MAPT		ENSG00000186868	microtubule associated protein tau
Q8N126	OID00290	CADM3		ENSG00000162706	cell adhesion molecule 3
P39905	OID00291	GDNF		ENSG00000168621	glial cell derived neurotrophic factor
O95185	OID00292	UNC5C		ENSG00000182168	unc-5 netrin receptor c
Q2TAL6	OID00293	VWC2		ENSG00000188730	von willebrand factor c domain containing 2
Q9Y336	OID00294	SIGLEC_9		ENSG00000129450	sialic acid binding Ig like lectin 9
Q08708	OID00295	CLM_6	CD300c	ENSG00000167850	CD300c molecule
P15311	OID00296	EZR		ENSG00000092820	ezrin
Q9H3U7	OID00297	SMOC2		ENSG00000112562	sparc related modular calcium binding 2
P41271	OID00298	NBL1		ENSG00000158747	nbl1, dan family bmp antagonist
P52798	OID00299	EFNA4		ENSG00000243364	ephrin a4
Q14108	OID00300	SCARB2		ENSG00000138760	scavenger receptor class b member 2
O14594	OID00301	NCAN		ENSG00000130287	neurocan
Q2VWP7	OID00302	PRTG		ENSG00000166450	protogenin
Q9HCK4	OID00303	ROBO2		ENSG00000185008	roundabout guidance receptor 2
O95727	OID00304	CRTAM		ENSG00000109943	cytotoxic and regulatory t cell molecule
Q96B86	OID00305	RGMA		ENSG00000182175	repulsive guidance molecule bmp co-receptor a
Q9ULL4	OID00306	PLXNB3		ENSG00000198753	plexin b3
P48052	OID00307	CPA2		ENSG00000158516	carboxypeptidase a2
P28907	OID00308	CD38		ENSG00000004468	CD38 molecule
P17405	OID00309	SMPD1		ENSG00000166311	sphingomyelin phosphodiesterase 1

Supplementary Table 1: List of all included proteins, included both through proximity extension assay (Olink) and Meso Scale Discovery.

UniProt ID	OlinkID	Protein abbreviation	Alternative protein abbreviation	EnsemblID (version109)	Full Protein Name
P21757	OID00310	MSR1		ENSG00000038945	macrophage scavenger receptor 1
P30533	OID00311	ALPHA_2_MRAP	LRPAP1	ENSG00000163956	LDL receptor related protein associated protein 1
Q92765	OID00312	S_FRP_3	FRZB	ENSG00000162998	frizzled related protein
O15197	OID00313	EPHB6		ENSG00000106123	eph receptor b6
Q6NW40	OID00314	RGMB		ENSG00000174136	repulsive guidance molecule bmp co-receptor b
Q9BZZ2	OID00315	SIGLEC1		ENSG00000088827	sialic acid binding Ig like lectin 1
O94779	OID00316	CNTN5		ENSG00000149972	contactin 5
Q9P0K1	OID00317	ADAM_22		ENSG00000008277	adam metallopeptidase domain 22
Q9P126	OID00318	CLEC1B		ENSG00000165682	c-type lectin domain family 1 member b
O75077	OID00319	ADAM_23		ENSG00000114948	adam metallopeptidase domain 23
O15232	OID00320	MATN3		ENSG00000132031	matrilin 3
Q2MKA7	OID00321	RSPO1		ENSG00000169218	r-spondin 1
Q16775	OID00322	HAGH		ENSG00000063854	hydroxyacylglutathione hydrolase
Q9BS40	OID00323	LXN		ENSG00000079257	latexin
O00214	OID00324	GAL_8	LGALS8	ENSG00000116977	galectin 8
Q96GW7	OID00325	BCAN		ENSG00000132692	brevican
Q6UX15	OID00326	LAYN		ENSG00000204381	layilin
P08473	OID00327	NEP	MME	ENSG00000196549	membrane metalloendopeptidase
O14793	OID00328	GDF_8	MSTN	ENSG00000138379	myostatin
P04216	OID00329	THY_1		ENSG00000154096	thy-1 cell surface antigen
Q96NZ8	OID00330	WFIKKN1		ENSG00000127578	wap, follistatin/kazal, immunoglobulin, kunitz and netrin domain containing 1
Q9H3S3	OID00331	TMPRSS5		ENSG00000166682	transmembrane serine protease 5
P22223	OID00332	CDH3		ENSG00000062038	cadherin 3

Supplementary Table 1: List of all included proteins, included both through proximity extension assay (Olink) and Meso Scale Discovery.

UniProt ID	OlinkID	Protein abbreviation	Alternative protein abbreviation	EnsemblID (version109)	Full Protein Name
P56159	OID00333	GFR_ALPHA_1	GFRA1	ENSG00000151892	gdnf family receptor alpha 1
P15509	OID00334	GM_CSF_R_ALPHA	CSF2RA	ENSG00000198223	colony stimulating factor 2 receptor subunit alpha
P01138	OID00335	BETA_NGF	NGF	ENSG00000134259	nerve growth factor
Q6ZMJ2	OID00336	SCARA5		ENSG00000168079	scavenger receptor class a member 5
P41217	OID00337	CD200		ENSG00000091972	cd200 molecule
Q16620	OID00338	NTRK2		ENSG00000148053	neurotrophic receptor tyrosine kinase 2
P12544	OID00339	GZMA		ENSG00000145649	granzyme a
P09919	OID00340	G_CSF	CSF3	ENSG00000108342	colony stimulating factor 3
Q8NBI3	OID00341	DRAXIN		ENSG00000162490	dorsal inhibitory axon guidance protein
Q96GP6	OID00342	SCARF2		ENSG00000244486	scavenger receptor class F member 2
O60609	OID00343	GDNFR_ALPHA_3	GFRA3	ENSG00000146013	GDNF family receptor alpha 3
P15151	OID00344	PVR		ENSG00000073008	pvr cell adheson molecule
Q9NP84	OID00345	TNFRSF12A		ENSG00000006327	tnf receptor superfamily member 12a
P37023	OID00346	SKR3	ACVRL1	ENSG00000139567	activin a receptor like type 1
O43155	OID00347	FLRT2		ENSG00000185070	fibronectin leucine rich transmembrane protein 2
P14384	OID00348	CPM		ENSG00000135678	carboxypeptidase m
Q8IUN9	OID00349	CLEC10A		ENSG00000132514	c-type lectin domain containing 10a
P78333	OID00350	GCP5	GPC5	ENSG00000179399	glypican 5
P12644	OID00351	BMP_4		ENSG00000125378	bone morphogenetic protein 4
Q96LA5	OID00352	FC_RL2	FCRL2	ENSG00000132704	Fc receptor like 2
Q8NFP4	OID00353	MDGA1		ENSG00000112139	mam domain containing glycosylphosphatidylinostol anchor 1
Q01344	OID00354	IL_5R_ALPHA	IL5RA	ENSG00000091181	interleukin 5 receptor subunit alpha
P16234	OID00355	PDGF_R_ALPHA	PDGFRA	ENSG00000134853	platelet derived growth factor receptor alpha

Supplementary Table 1: List of all included proteins, included both through proximity extension assay (Olink) and Meso Scale Discovery.

UniProt ID	OlinkID	Protein abbreviation	Alternative protein abbreviation	EnsemblID (version109)	Full Protein Name
P53634	OID00356	CTSC		ENSG00000109861	cathepsin c
P55285	OID00357	CDH6		ENSG00000113361	cadherin 6
Q08345	OID00358	DDR1		ENSG00000204580	discoidin domain receptor tyrosine kinase 1
P57087	OID00359	JAM_B	JAM2	ENSG00000154721	junctional adhesion molecule 2
P25774	OID00360	CTSS		ENSG00000163131	cathepsin s
Q9NR71	OID00361	N_C_DASE	ASAH2	ENSG00000188611	n-acylsphingosine amidohydrolase 2
Q02083	OID00362	NAAA		ENSG00000138744	n-acylethanolamine acid amidase
Q9BZM5	OID00363	N2DL_2	ULBP2	ENSG00000131015	UL16 binding protein 2
O43157	OID00364	PLXNB1		ENSG00000164050	plexin b1
O75509	OID00365	TNFRSF21		ENSG00000146072	tnf receptor superfamily member 21
Q8TDQ1	OID00366	CLM_1	CD300LF	ENSG00000186074	CD300 molecule like family member f
Q08629	OID00367	SPOCK1		ENSG00000152377	SPARC (osteonectin) cwcv and kazal like domains proteoglycan 1
P29460	OID00368	IL12	IL12B	ENSG00000113302	interleukin 12b
P29459	OID00368	IL12	IL12A	ENSG00000168811	interleukin 12a
Q9UBT3	OID00369	DKK_4		ENSG00000104371	dickkopf wnt signaling pathway inhibitor 4
Q9HAV5	OID00370	EDA2R		ENSG00000131080	ectodysplasin a2 receptor
O43561	OID00371	LAT		ENSG00000213658	linker for activation of t cells
Q16288	OID00372	NTRK3		ENSG00000140538	neurotrophic receptor tyrosine kinase 3
Q6ISS4	OID00373	LAIR_2		ENSG00000167618	leukocyte associated immunoglobulin like receptor 2
P55145	OID00374	MANF		ENSG00000145050	mesencephalic astrocyte derived neurotrophic factor
Q92752	OID00375	TN_R		ENSG00000116147	tenascin r
Q8TD46	OID00376	CD200R1		ENSG00000163606	cd200 receptor 1
Q92823	OID00377	NR_CAM		ENSG00000091129	neuronal cell adhesion molecule

Supplementary Table 1: List of all included proteins, included both through proximity extension assay (Olink) and Meso Scale Discovery.

UniProt ID	OlinkID	Protein abbreviation	Alternative protein abbreviation	EnsemblID (version109)	Full Protein Name
Q16719	OID00378	KYNU		ENSG00000115919	kynureninase
P01584	NA	IL_1beta	IL1B	ENSG00000125538	interleukin 1 beta
P05231	NA	IL_6	IL6	ENSG00000136244	interleukin 6
P10145	NA	IL_8	CXCL8	ENSG00000169429	c-x-c motif chemokine ligand 8
P15692	NA	VEGF	VEGFA	ENSG00000112715	vascular endothelial growth factor a
P23560	NA	hu_BDNF	BDNF	ENSG00000176697	brain derived neurotrophic factor
P01138	NA	hu_betaNGF	NGFB	ENSG00000134259	nerve growth factor
P05067	NA	Abeta_38	APP	ENSG00000142192	amyloid beta precursor protein
P05067	NA	Abeta_40	APP	ENSG00000142192	amyloid beta precursor protein
P05067	NA	Abeta_42	APP	ENSG00000142192	amyloid beta precursor protein

All proteins examined in the study. For proteins analyzed utilizing proximity extension assay, OLINK identification number is reported in addition to UniProt ditto. For enrichment analyses utilizing RNA sequencing data, the Ensembl ID (version 109) was used.

Supplementary Table 2: Missing values per protein assessed

Protein Abbreviation	Count missing	Percentage missing
BETA_NGF	52	100
BMP_4	52	100
CLEC10A	52	100
FC_RL2	51	98.1
NEP	51	98.1
WFIKKN1	51	98.1
IL_5R_ALPHA	48	92.3
LAT	45	86.5
CDH3	44	84.6
CDH6	43	82.7
NAAA	43	82.7
PRTG	38	73.1
GDF_8	35	67.3
MDGA1	35	67.3
LXN	33	63.5
SIGLEC1	33	63.5
SMPD1	31	59.6
RSPO1	29	55.8
S_FRP_3	25	48.1
GDNF	24	46.2
Abeta_38*	21	40.4
IL12	20	38.5
KYNU	18	34.6
ROBO2	18	34.6
SCARA5	18	34.6
UNC5C	18	34.6
VWC2	13	25
CRTAM	10	19.2
SCARF2	10	19.2
GDNFR_ALPHA_3	8	15.4
FLRT2	7	13.5
ADAM_23	6	11.5
Abeta_42*	6	11.5
NMNAT1	6	11.5
EFNA4	5	9.6
PLXNB3	5	9.6
hu_BDNF*	5	9.6
CADM3	3	5.8
CNTN5	3	5.8
NRP2	3	5.8
Abeta_40*	2	3.8

Supplementary Table 2: Missing values per protein assessed

Protein Abbreviation	Count missing	Percentage missing
EPHB6	2	3.8
GCP5	2	3.8
SPOCK1	2	3.8
TMPRSS5	2	3.8
BCAN	1	1.9
DKK_4	1	1.9
GM_CSF_R_ALPHA	1	1.9
G_CSF	1	1.9
HAGH	1	1.9
MANF	1	1.9
MAPT	1	1.9
PDGF_R_ALPHA	1	1.9
TN_R	1	1.9
VEGF*	1	1.9
ADAM_22	0	0
ALPHA_2_MRAP	0	0
CD200	0	0
CD200R1	0	0
CD38	0	0
CLEC1B	0	0
CLM_1	0	0
CLM_6	0	0
CPA2	0	0
CPM	0	0
CTSC	0	0
CTSS	0	0
DDR1	0	0
DRAXIN	0	0
EDA2R	0	0
EZR	0	0
GAL_8	0	0
GFR_ALPHA_1	0	0
GZMA	0	0
IL_1beta*	0	0
IL_6*	0	0
IL_8*	0	0
JAM_B	0	0
LAIR_2	0	0
LAYN	0	0
MATN3	0	0
MSR1	0	0

Supplementary Table 2: Missing values per protein assessed

Protein Abbreviation	Count missing	Percentage missing
N2DL_2	0	0
NBL1	0	0
NCAN	0	0
NR_CAM	0	0
NTRK2	0	0
NTRK3	0	0
N_C_DASE	0	0
PLXNB1	0	0
PVR	0	0
RGMA	0	0
RGMB	0	0
SCARB2	0	0
SIGLEC_9	0	0
SKR3	0	0
SMOC2	0	0
THY_1	0	0
TNFRSF12A	0	0
TNFRSF21	0	0
hu_betaNGF*	0	0

The extent of missing values (count and rounded percentages) per proteins (all sample time points, all study subjects). Full protein names are described in **Supplementary Table 1**. Proteins denoted with * were analyzed using Meso Scale Discovery Inc. and the rest were analyzed using the proximity extension assay technology. The percentage missing column was rounded to one decimal for convenience.

Supplementary Table 3: Relationship between protein level and time from trauma

Protein abbreviation	t_{time}	$p_{unadjusted}$	$p_{adjusted}$
ADAM_22	-4.92	0	0
α-2-MRAP	-2.997	0.006	0.018
Aβ-40	2.203	0.044	0.096
BCAN	-2.742	0.034	0.08
CD200	-5.346	0	0
CLEC1B	-5.161	0	0
CLM-1	3.172	0.004	0.013
CLM-6	9.788	0	0
CTSC	5.097	0	0
CTSS	9.741	0	0
DDR1	-5.378	0	0
DKK-4	-2.432	0.042	0.094
DRAXIN	7.703	0	0
EZR	-3.641	0.001	0.004
FLRT2	-3.426	0.025	0.061
GAL-8	-7.374	0	0
GCP5	-3.534	0.006	0.019
GFR-α-1	-2.145	0.042	0.094
IL-1β	-2.878	0.008	0.023
IL-8	-9.088	0	0
JAM_B	-6.061	0	0
LAIR_2	-6.746	0	0
LAYN	-5.72	0	0
MAPT	-2.852	0.032	0.077
MATN3	5.206	0	0
MSR1	11.861	0	0
NBL1	-2.795	0.01	0.027
NCAN	-4.436	0	0.001
NTRK2	-4.097	0	0.002
NTRK3	-3.524	0.002	0.006
PDGF-R-α	-3.687	0.006	0.018
PLXNB3	-3.369	0.022	0.056
RGMA	-5.372	0	0
RGMB	-4.077	0	0.002
SIGLEC-9	4.151	0	0.001
SKR3	4.835	0	0
SMOC2	7.478	0	0
TMPRSS5	-7.949	0	0
TNFRSF12A	9.291	0	0
TNFRSF21	-3.948	0.001	0.002
Hu-βNGF	2.576	0.016	0.043

Univariate linear mixed model depicting protein level as the dependent variable and time from trauma as the independent variable. Above, the models t statistic (t_{time}), the unadjusted p value ($p_{unadjusted}$) and the false-discovery rate adjusted p value ($p_{adjusted}$) are presented for proteins, where protein levels were significantly influenced by the time from trauma ($p_{unadjusted} \leq 0.05$). For t_{time} and $p_{unadjusted}$, the mean value from all pooled imputations are presented. Patient identity was used as random effect. No correlation structure was used. Full protein names are described in **Supplementary Table 1**.

Supplementary Table 4: Relationship between protein level and patient age

Protein Abbreviation	$Coefficient_{age}$	$p_{unadjusted}$	$p_{adjusted}$
EDA2R	0.039	0.005	0.41
HAGH	-0.03	0.036	0.669
MANF	-0.037	0.045	0.669
SPOCK1	0.029	0.027	0.669

Univariate linear mixed model depicting protein level as the dependent variable and patient age as the independent variable. Above, the models coefficient ($coefficient_{age}$), the unadjusted p value ($p_{unadjusted}$) and the false-discovery rate adjusted p value ($p_{adjusted}$) are presented for proteins, where protein levels were significantly influenced by the patient's age ($p_{unadjusted} \leq 0.05$). For $coefficient_{age}$ and $p_{unadjusted}$, the mean value from all pooled imputations are presented. Patient identity was used as random effect. No correlation structure was used. Full protein names are described in **Supplementary Table 1**.

Supplementary Table 5: Protein expression levels of CLM-1 and GFR-α1 depend on APOE single-nucleotide polymorphism and time from trauma.

**Protein abbreviation**	**$Coef_{interaction}$**	**$p_{interaction\_unadjusted}$**	**$p_{interaction\_adjusted}$**	**$Coef_{APOE\epsilon4}$**	**$p_{APOE\epsilon4\_unadjusted}$**	**$Coef_{time}$**	**$p_{time\_unadjusted}$**
CLM-1	0.396	**0.039**	0.899	-0.128	0.707	0.124	0.322
GFR-α1	0.238	**0.02**	0.899	-0.09	0.609	-0.222	0.002

Multivariable linear mixed model depicting protein level as the dependent variable and APOE ϵ4 single-nucleotide polymorphism and time from trauma as independent variables, modelled as an interaction term. Above, the model's coefficients ($Coef_{interaction}$, $Coef_{APOE\epsilon4}$, $Coef_{tim}$), and the unadjusted p values ($p_{interaction\_unadjusted}$, $p_{APOE\epsilon4\_unadjusted}$, $p_{time\_unadjusted}$) are presented for the proteins. For the interaction term, we also present the false-discovery rate adjusted p value ($p_{interaction\_adjusted}$). The model coefficients and the unadjusted p values were derived through calculation of the mean value from all pooled imputations. Patient identity was used as random effect. No correlation structure was used. Full protein names are described in **Supplementary Table 1**. Abbreviations: APOE, apolipoprotein E.

Supplementary Figure Legends

Supplementary Figure 1: Longitudinal protein levels in brain extracellular fluid per patient following severe TBI. Protein analysis was undertaken at the first and third day following trauma. Notably, several proteins of varying origin and functions displayed a longitudinal trajectory. In total, n = 41 proteins demonstrated a temporal trajectory (n = 34 proteins retained significance following multiple testing correction). Here, no adjustment to apolipoprotein E status is undertaken. All protein concentrations are normalized to day 1 levels and represent ratios. Full protein names are displayed in **Supplementary Table 1**.

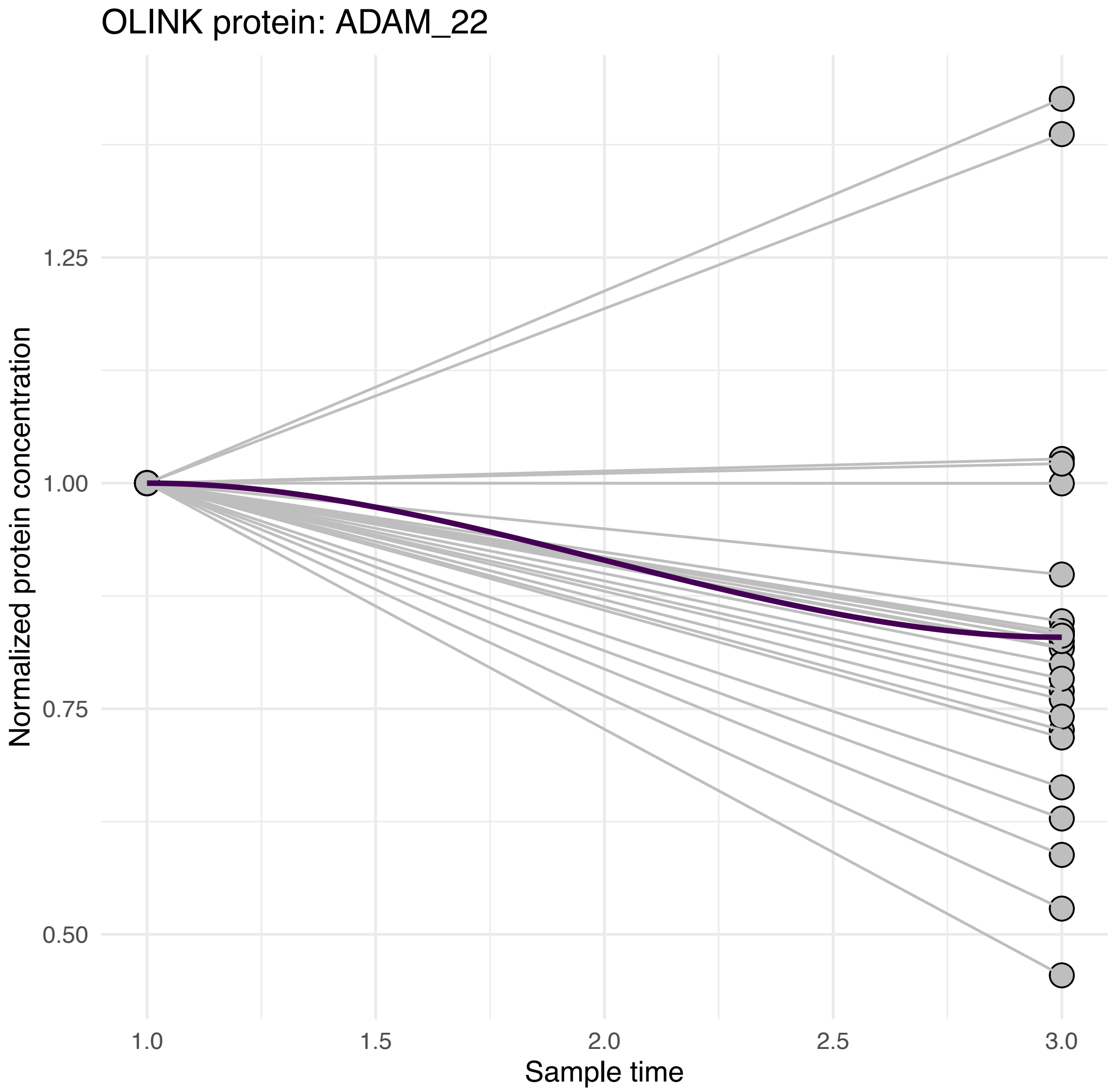
OLINK protein: ADAM_22
Normalized protein concentration
1.25
1.00
0.75
0.50
1.0
1.5
2.0
2.5
3.0
Sample time

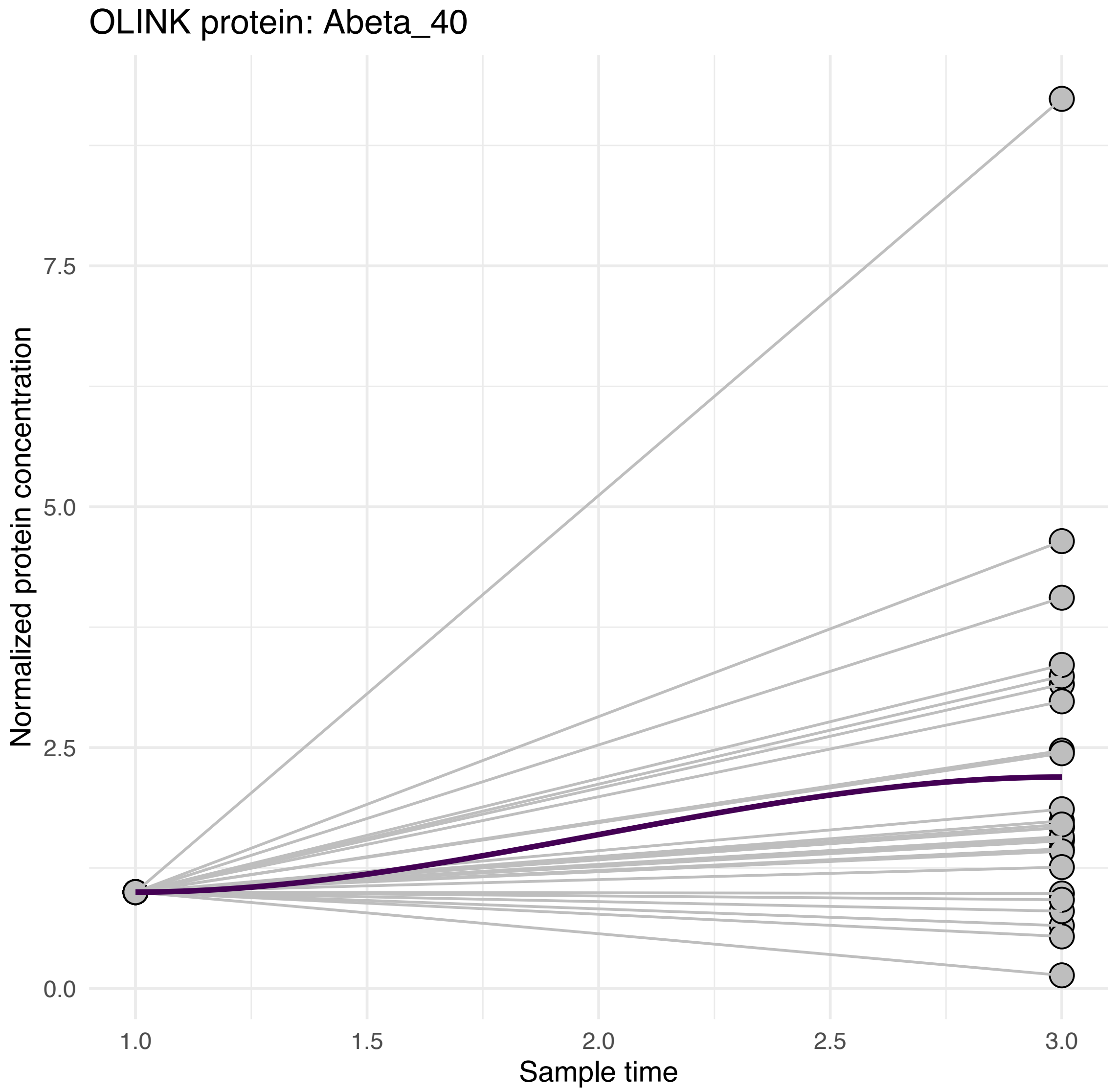
OLINK protein: Abeta_40
Normalized protein concentration
7.5
5.0
2.5
0.0
1.0
1.5
2.0
2.5
3.0
Sample time

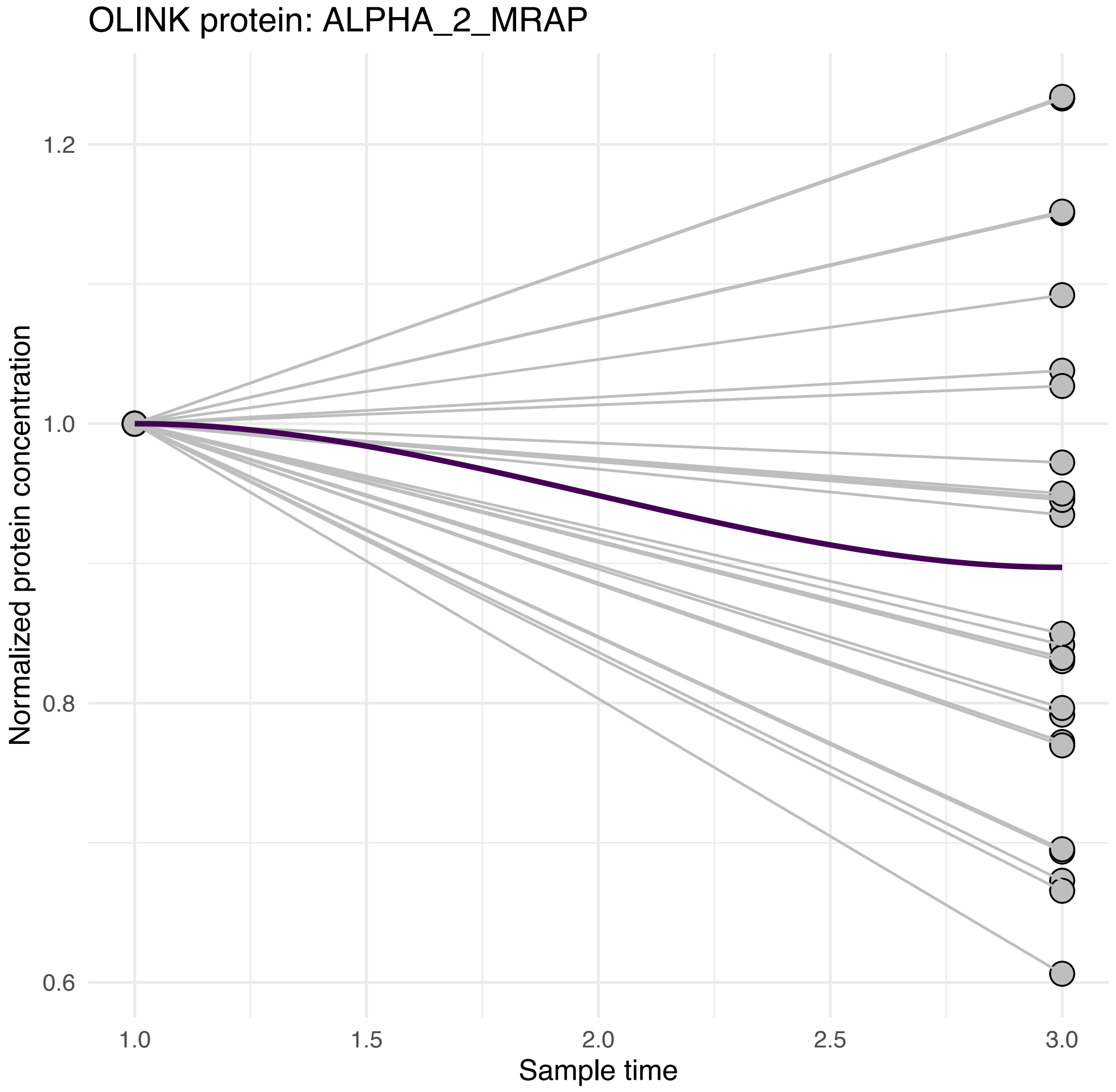
OLINK protein: ALPHA_2_MRAP
Normalized protein concentration
1.2
1.0
0.8
0.6
1.0
1.5
2.0
2.5
3.0
Sample time

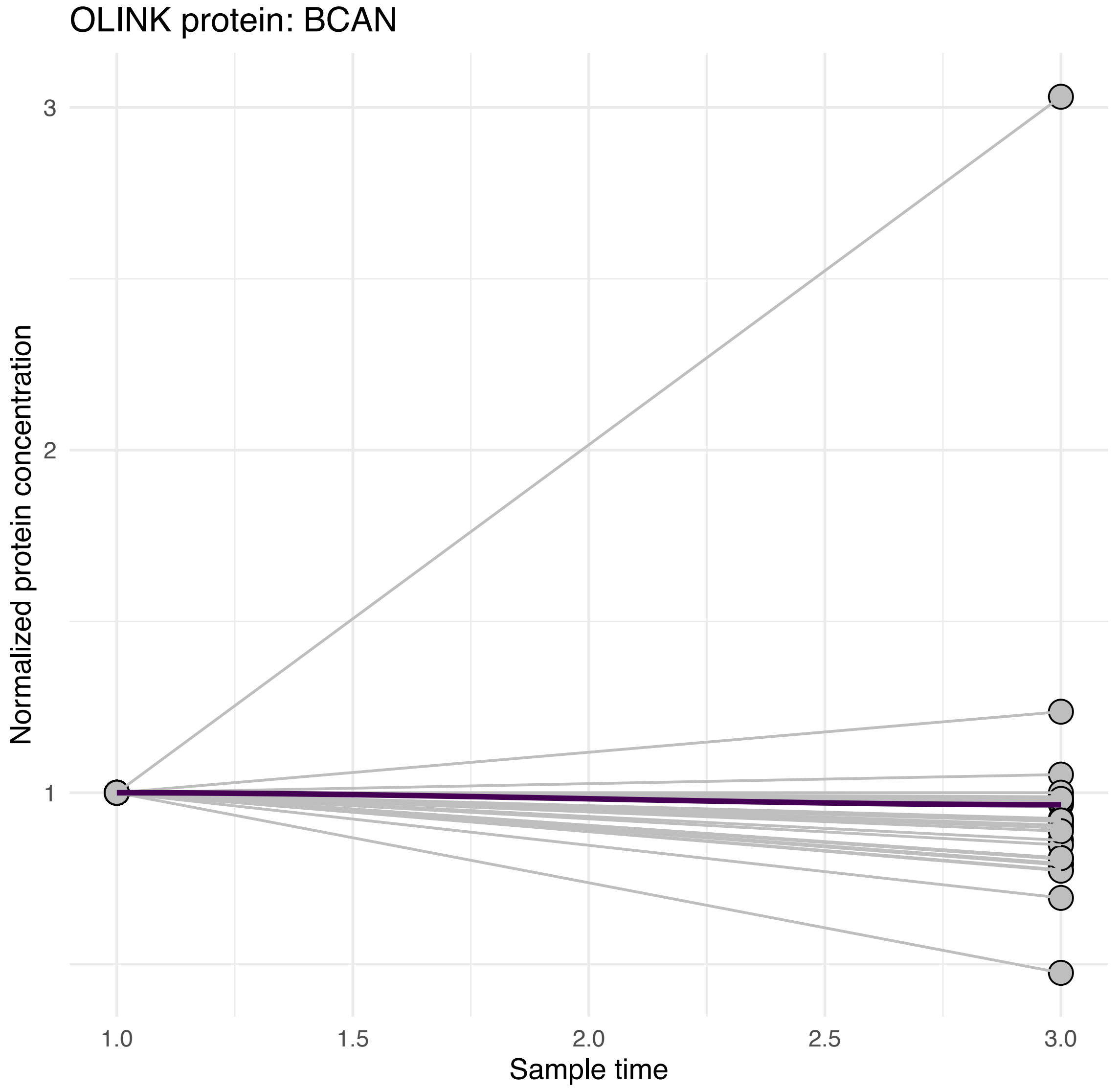
OLINK protein: BCAN
Normalized protein concentration
3
2
1
1.0
1.5
2.0
2.5
3.0
Sample time

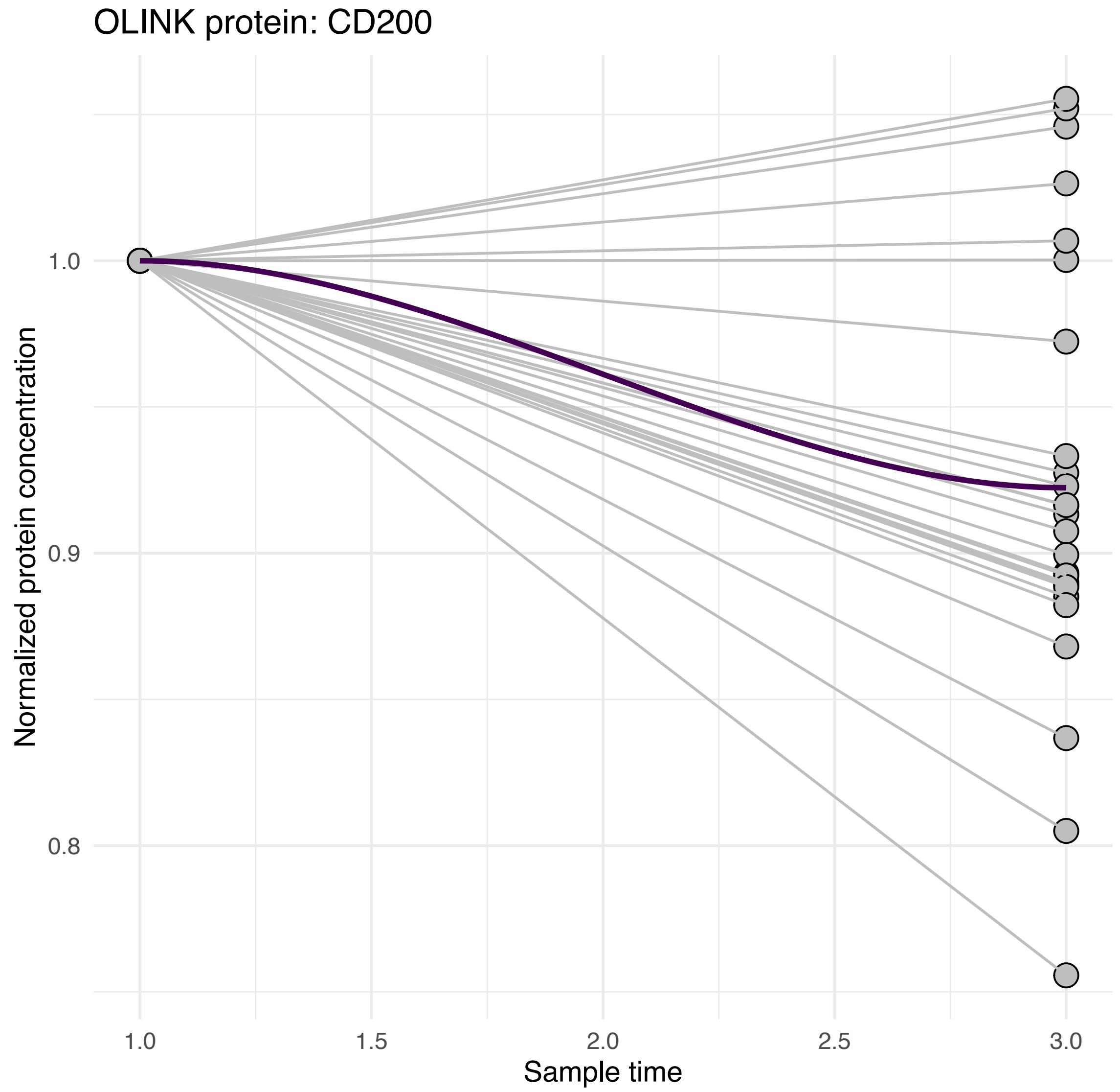

OLINK protein: CD200
Normalized protein concentration
1.0
0.9
0.8
1.0
1.5
2.0
2.5
3.0
Sample time

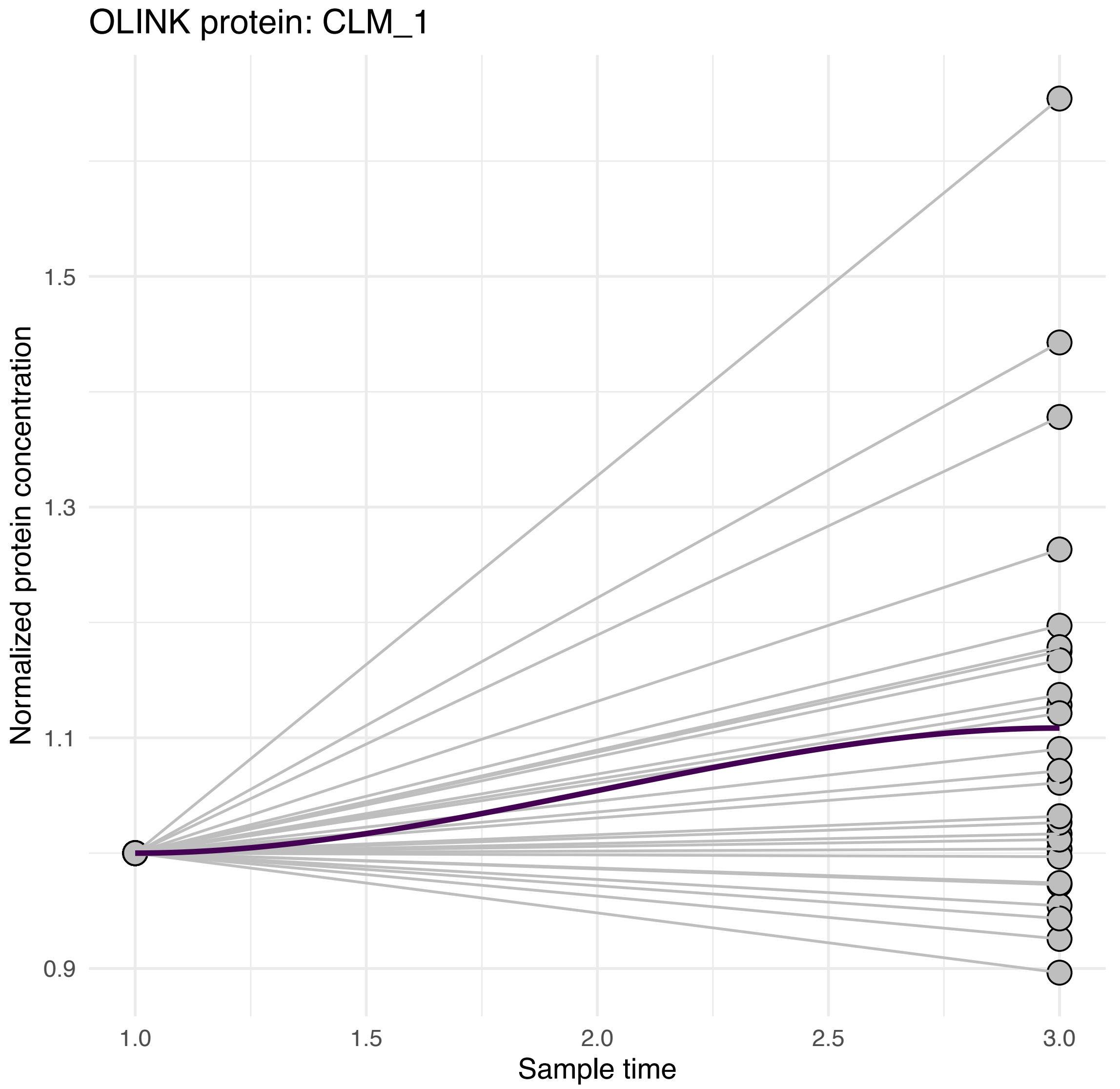

OLINK protein: CLM_1
Normalized protein concentration
1.5
1.3
1.1
0.9
1.0
1.5
2.0
2.5
3.0
Sample time

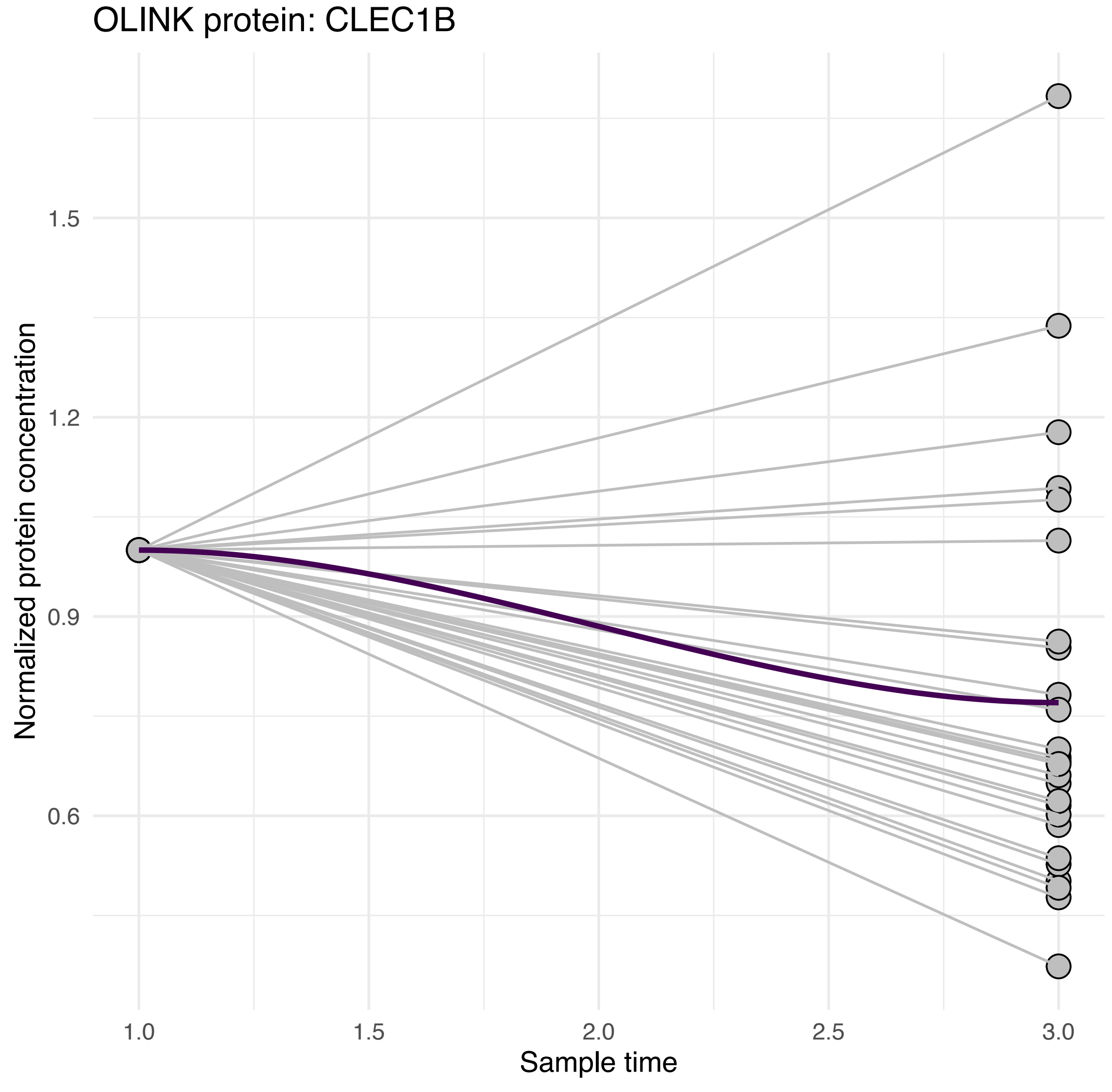

OLINK protein: CLEC1B
Normalized protein concentration
1.5
1.2
0.9
0.6
1.0
1.5
2.0
2.5
3.0
Sample time

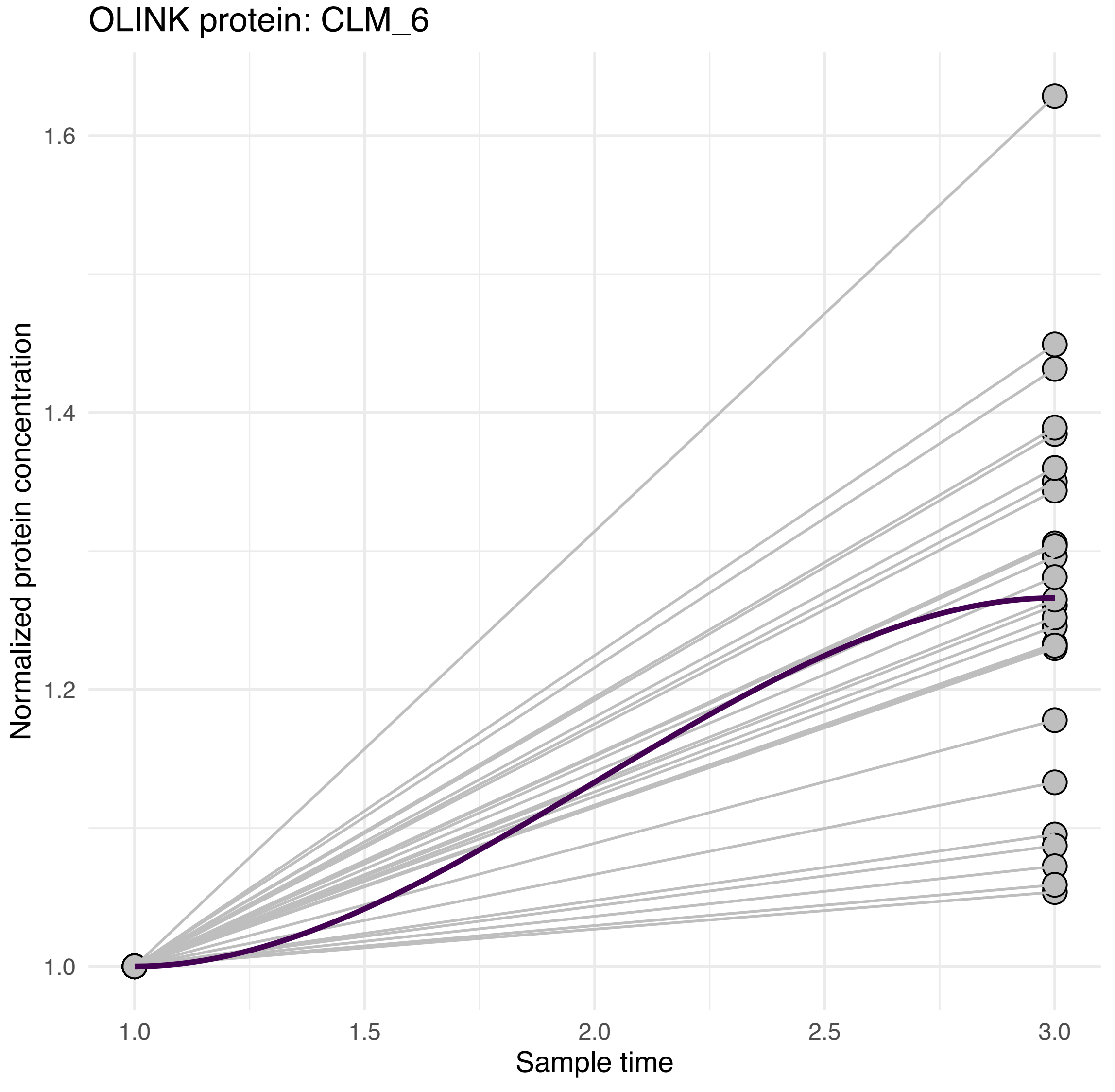

OLINK protein: CLM_6
Normalized protein concentration
1.6
1.4
1.2
1.0
1.0
1.5
2.0
2.5
3.0
Sample time

OLINK protein: CTSC

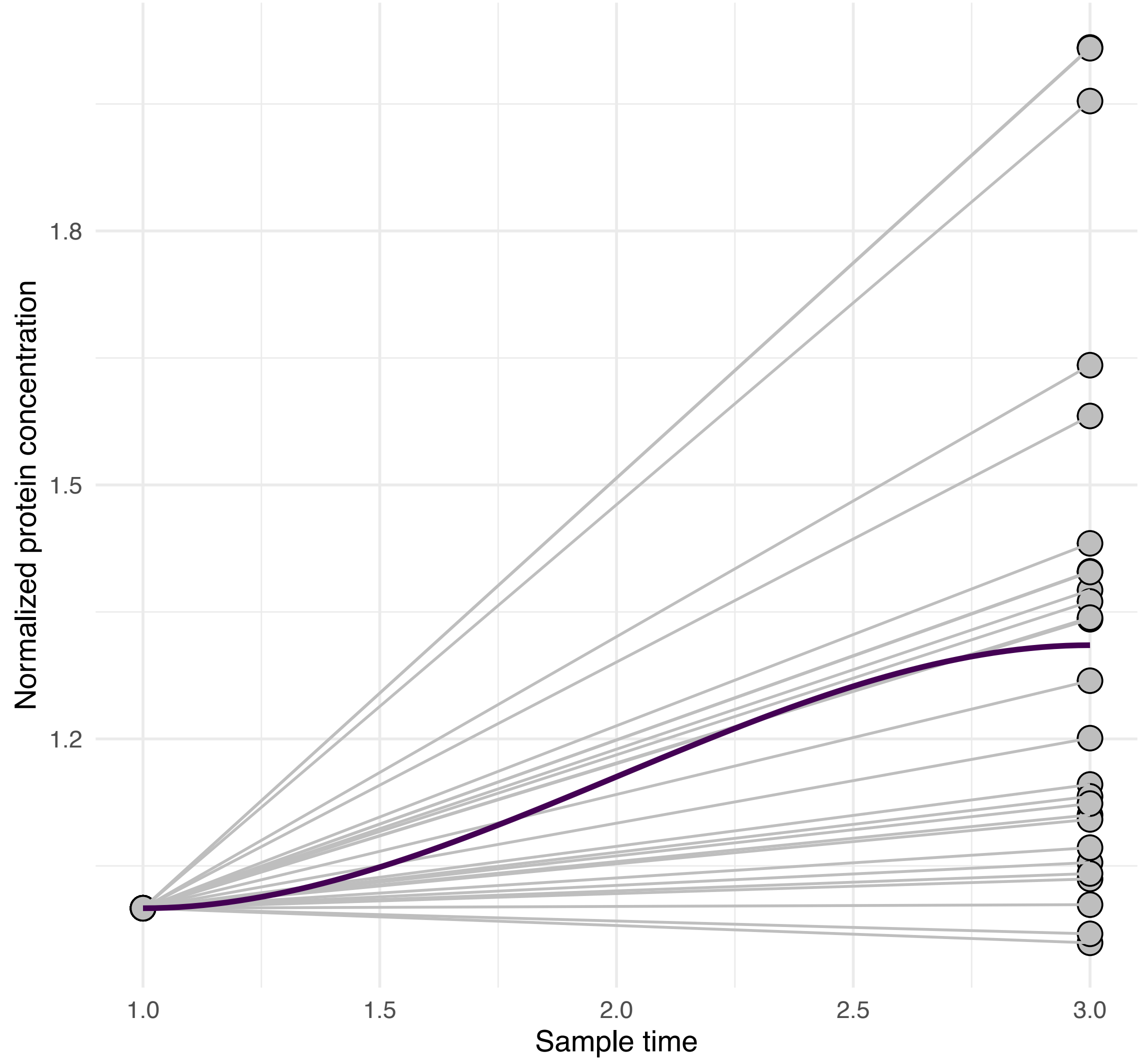

OLINK protein: DDR1

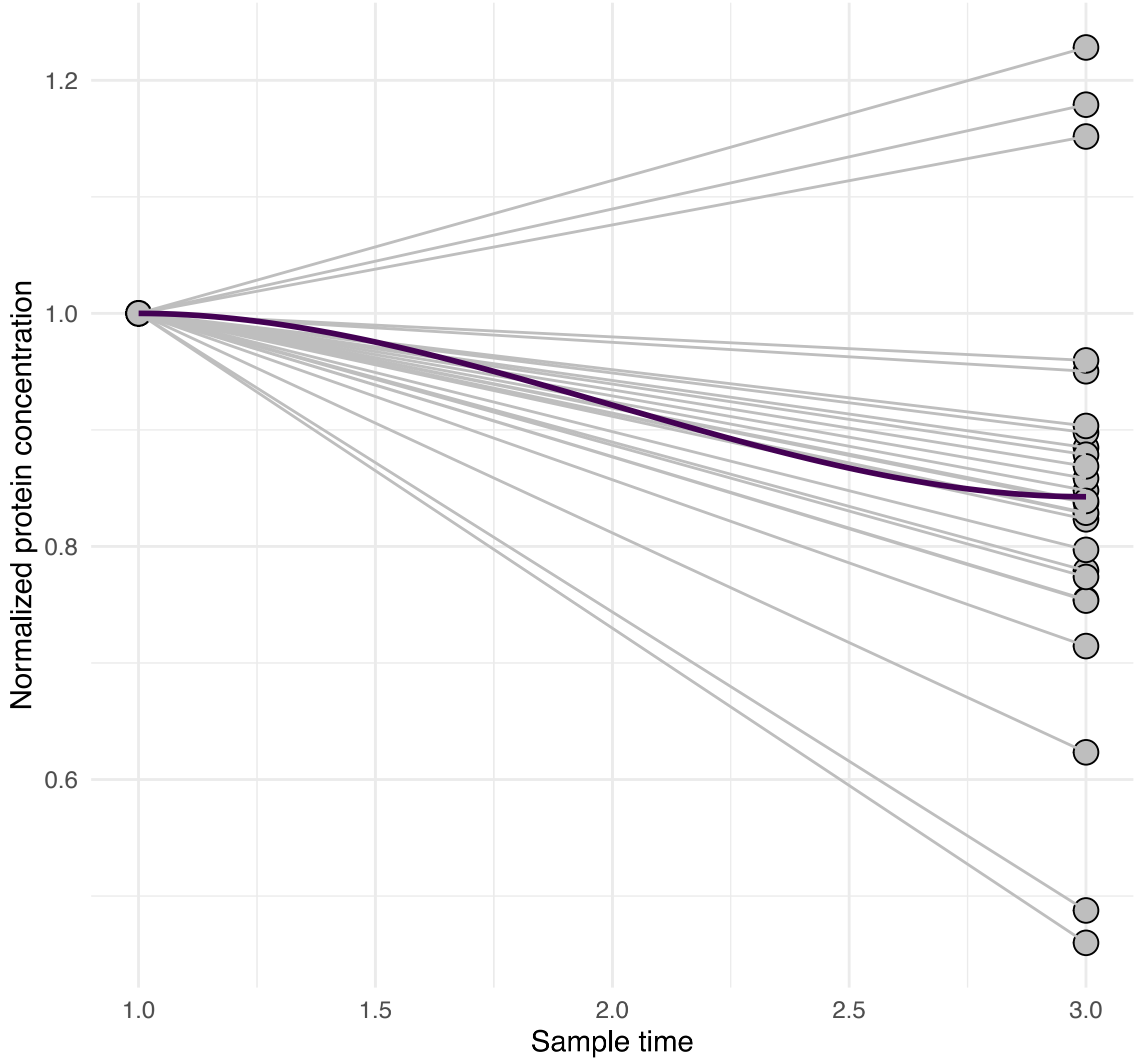

OLINK protein: CTSS

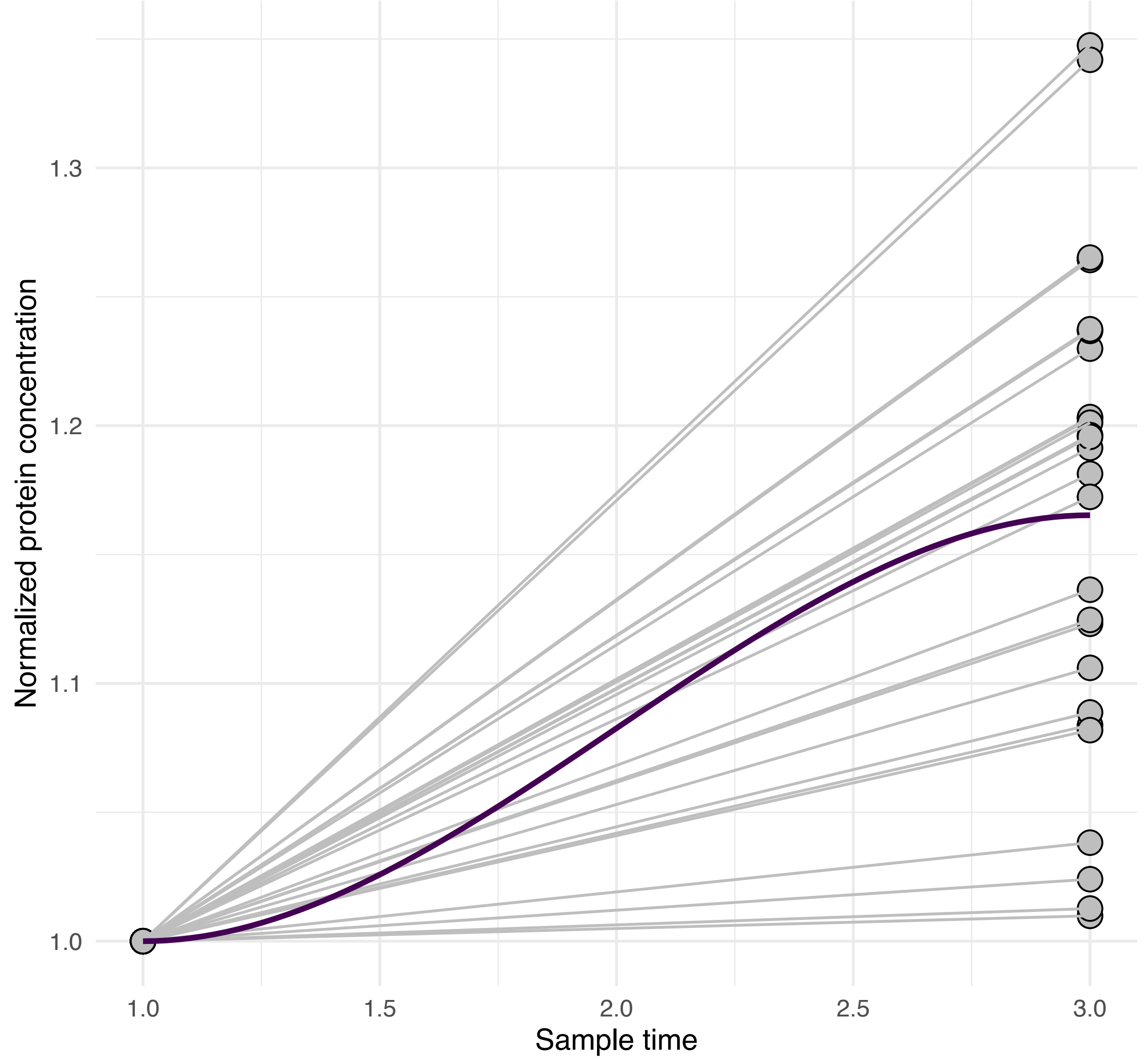

OLINK protein: DKK_4

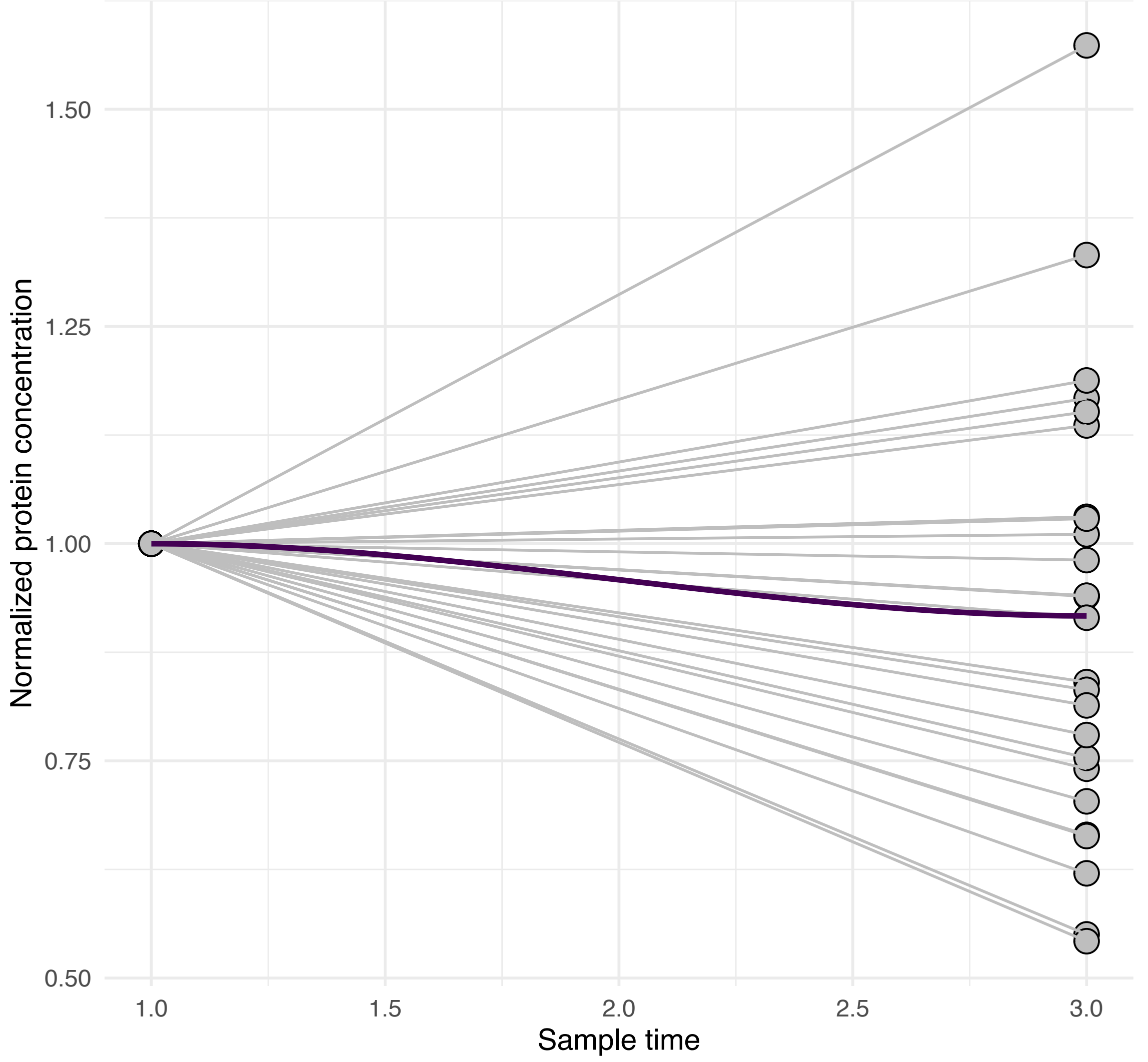

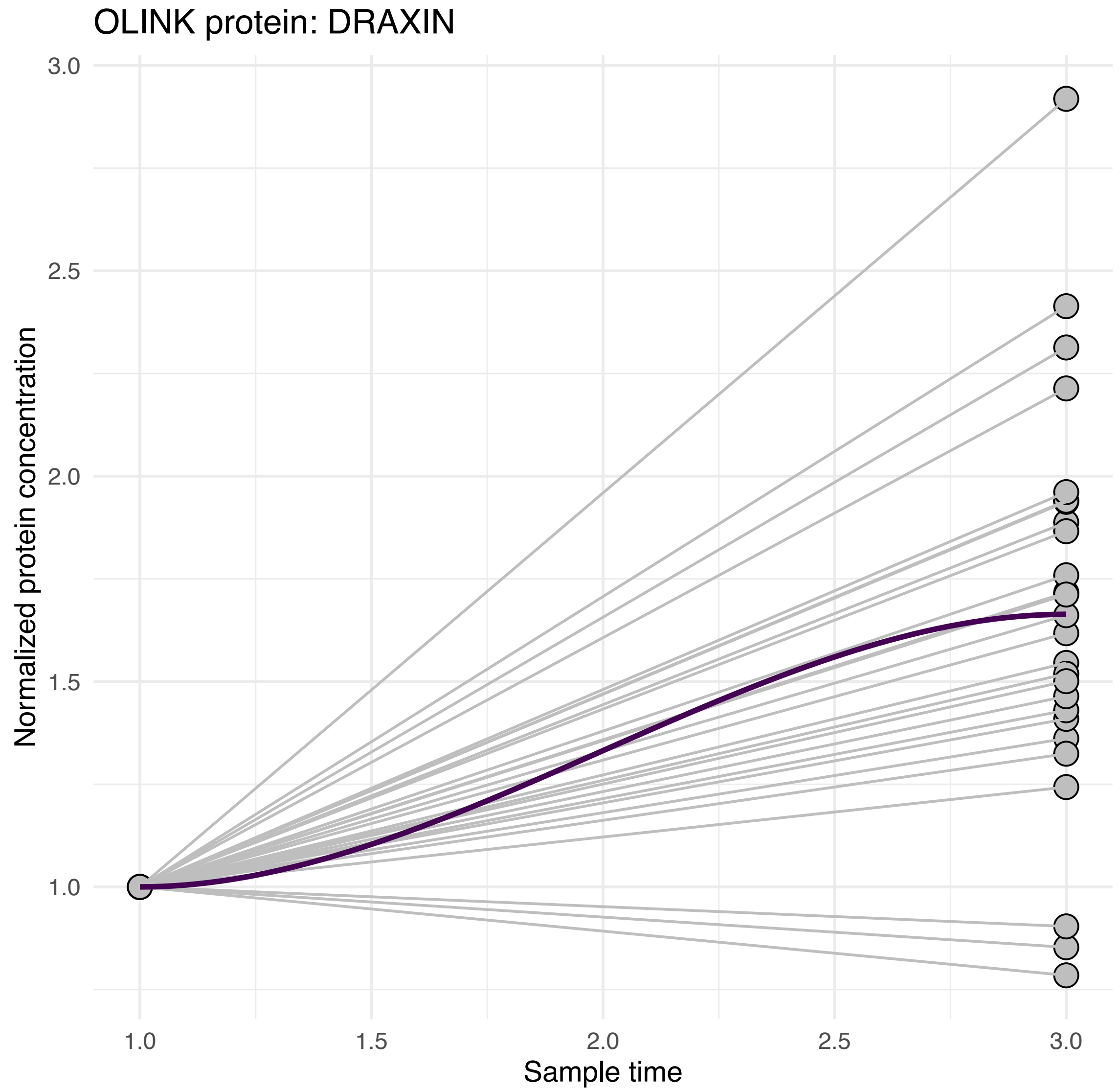
OLINK protein: DRAXIN
Normalized protein concentration
Sample time
1.0
1.5
2.0
2.5
3.0

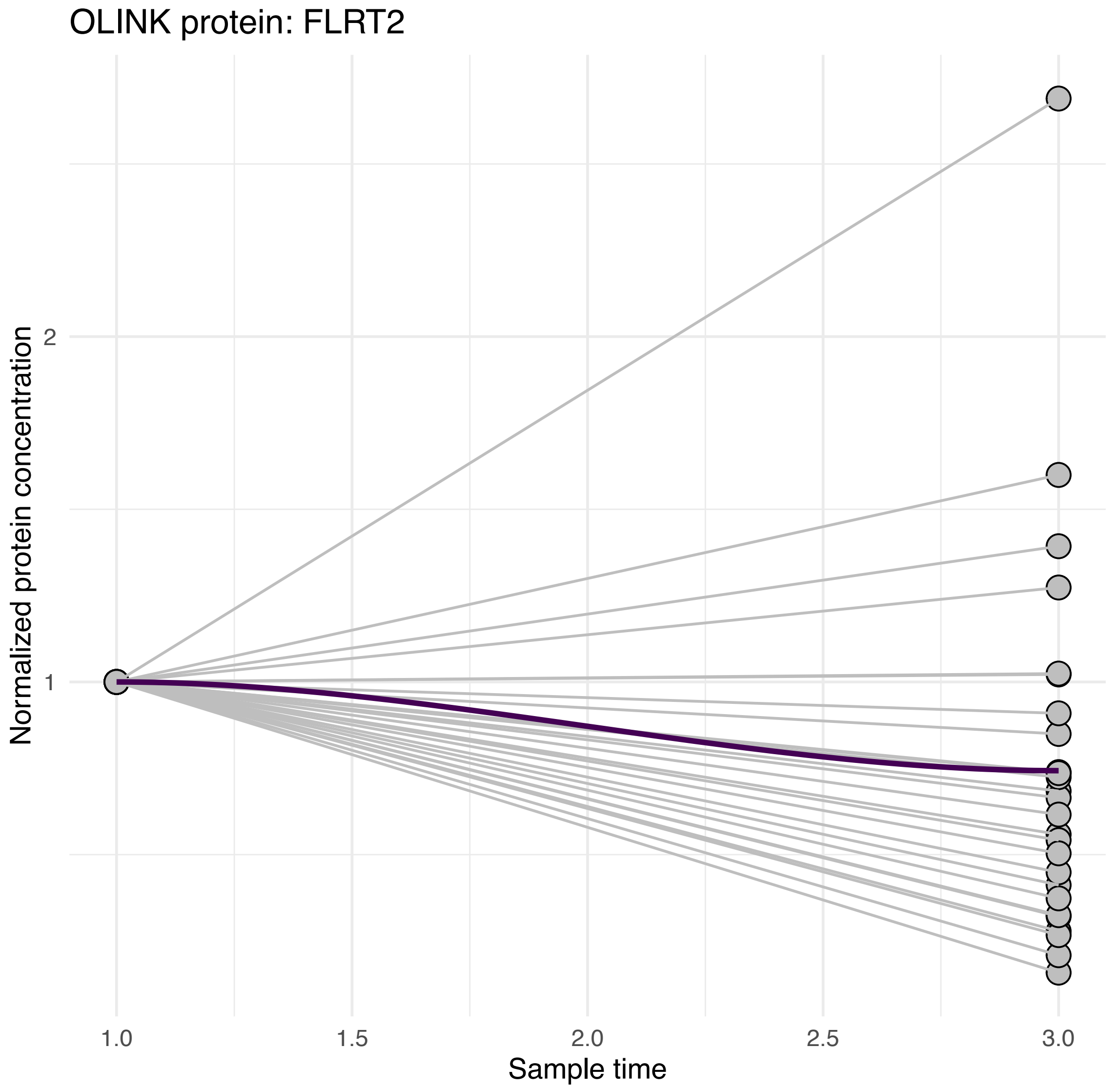
OLINK protein: FLRT2
Normalized protein concentration
Sample time
1
2
1.0
1.5
2.0
2.5
3.0

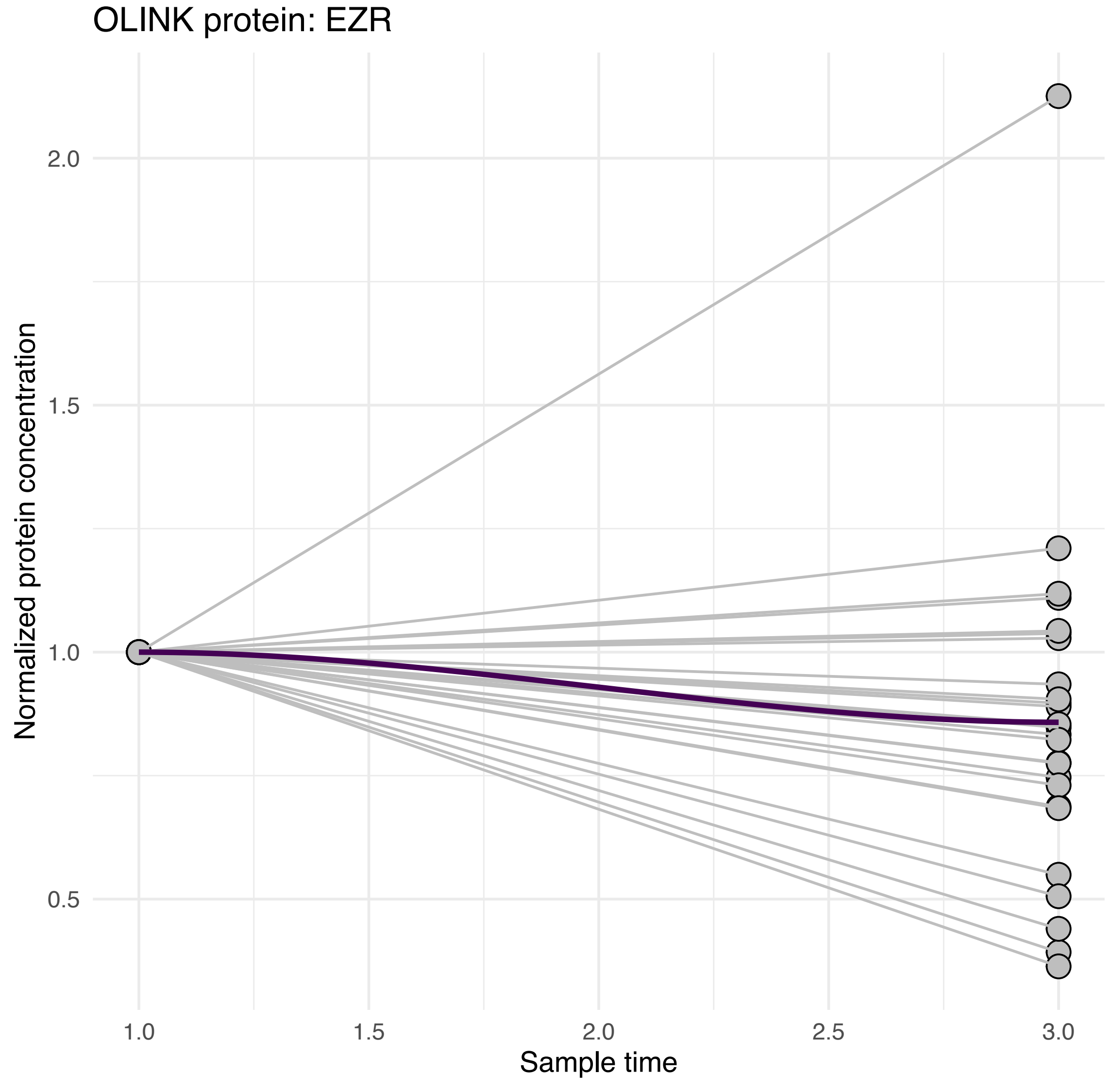
OLINK protein: EZR
Normalized protein concentration
Sample time
0.5
1.0
1.5
2.0
2.5
3.0

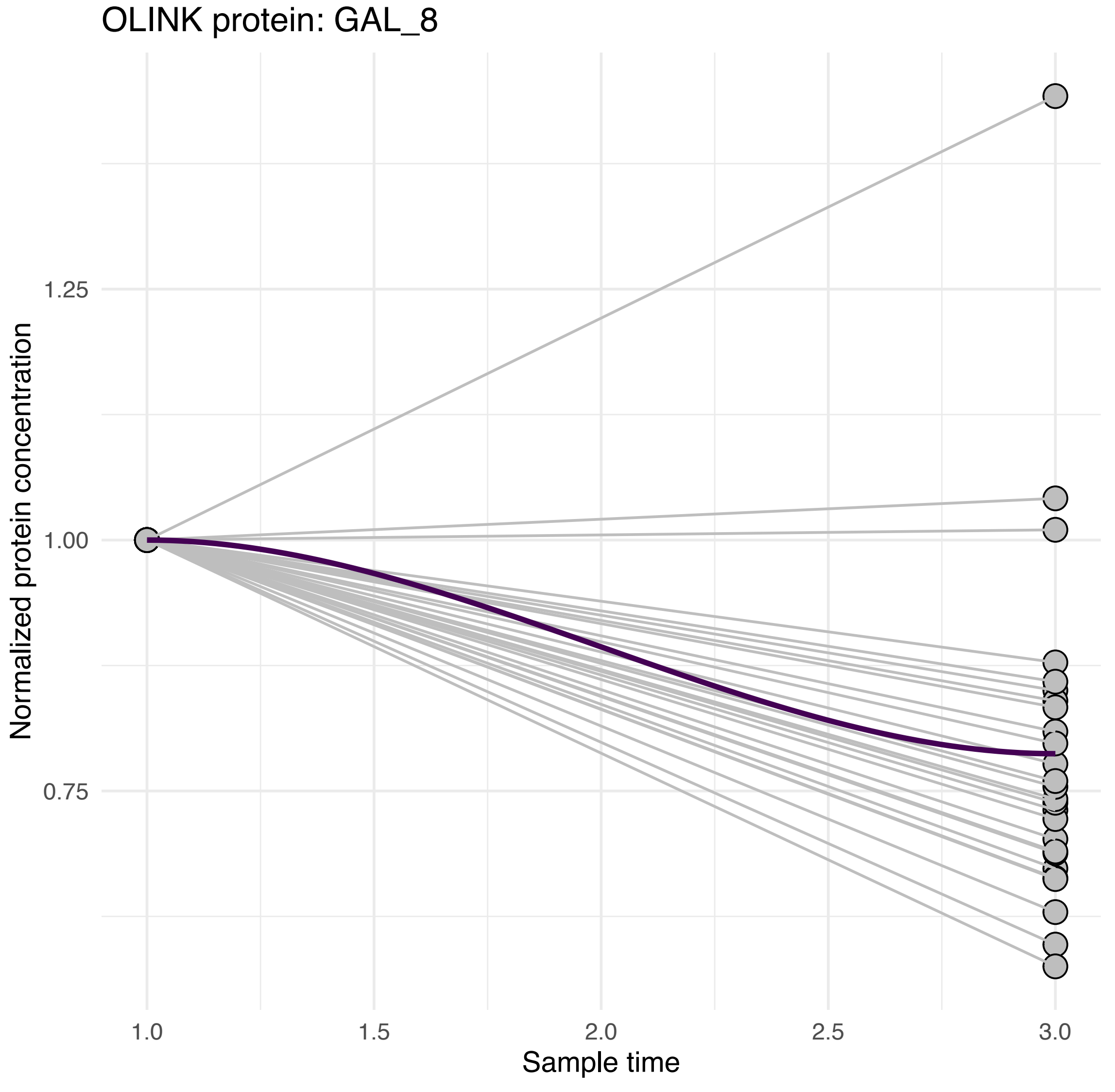
OLINK protein: GAL_8
Normalized protein concentration
Sample time
0.75
1.00
1.25
1.0
1.5
2.0
2.5
3.0

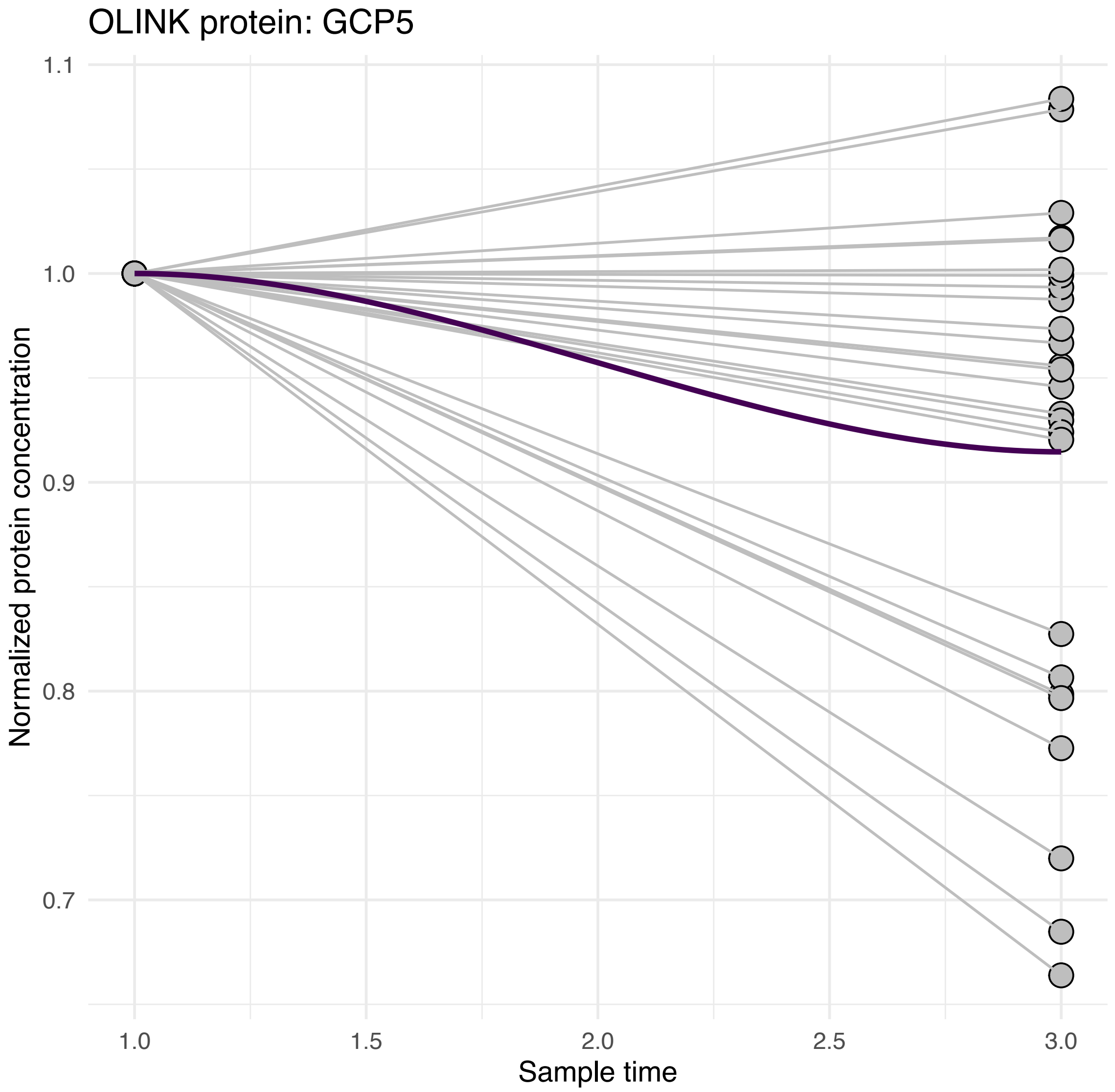
OLINK protein: GCP5
Normalized protein concentration
1.1
1.0
0.9
0.8
0.7
Sample time
1.0
1.5
2.0
2.5
3.0

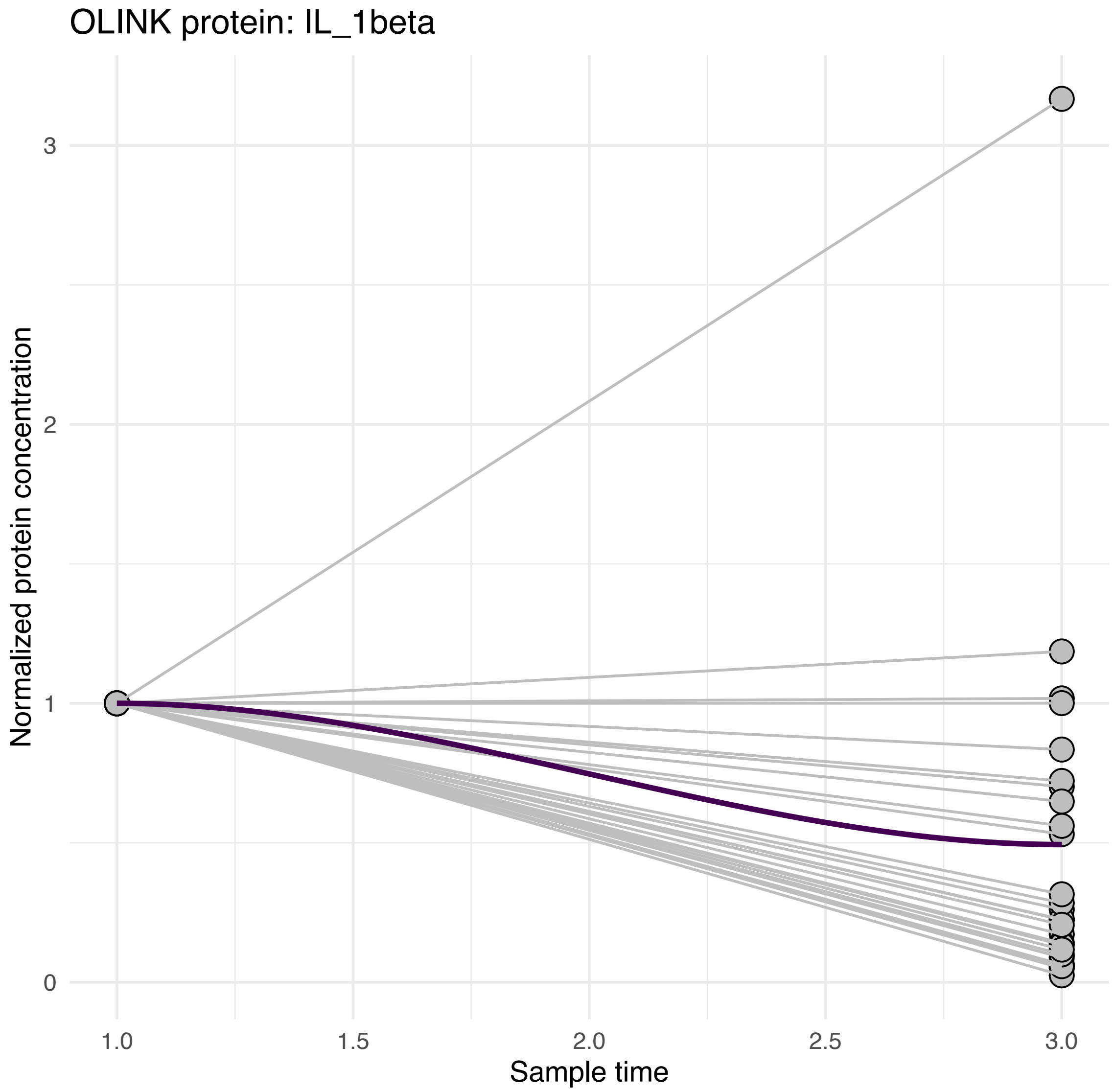
OLINK protein: IL_1beta
Normalized protein concentration
3
2
1
0
Sample time
1.0
1.5
2.0
2.5
3.0

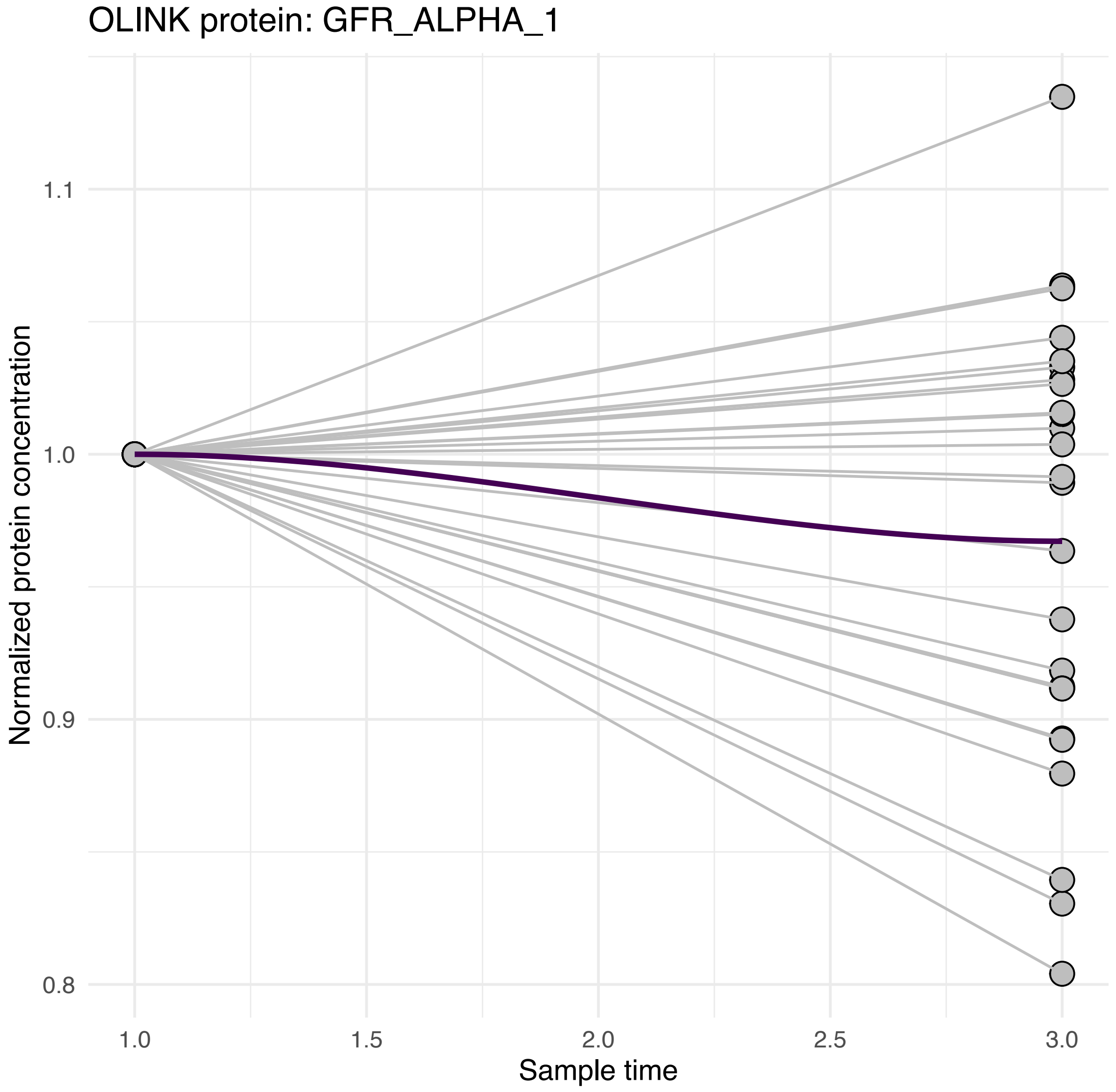
OLINK protein: GFR_ALPHA_1
Normalized protein concentration
1.1
1.0
0.9
0.8
Sample time
1.0
1.5
2.0
2.5
3.0

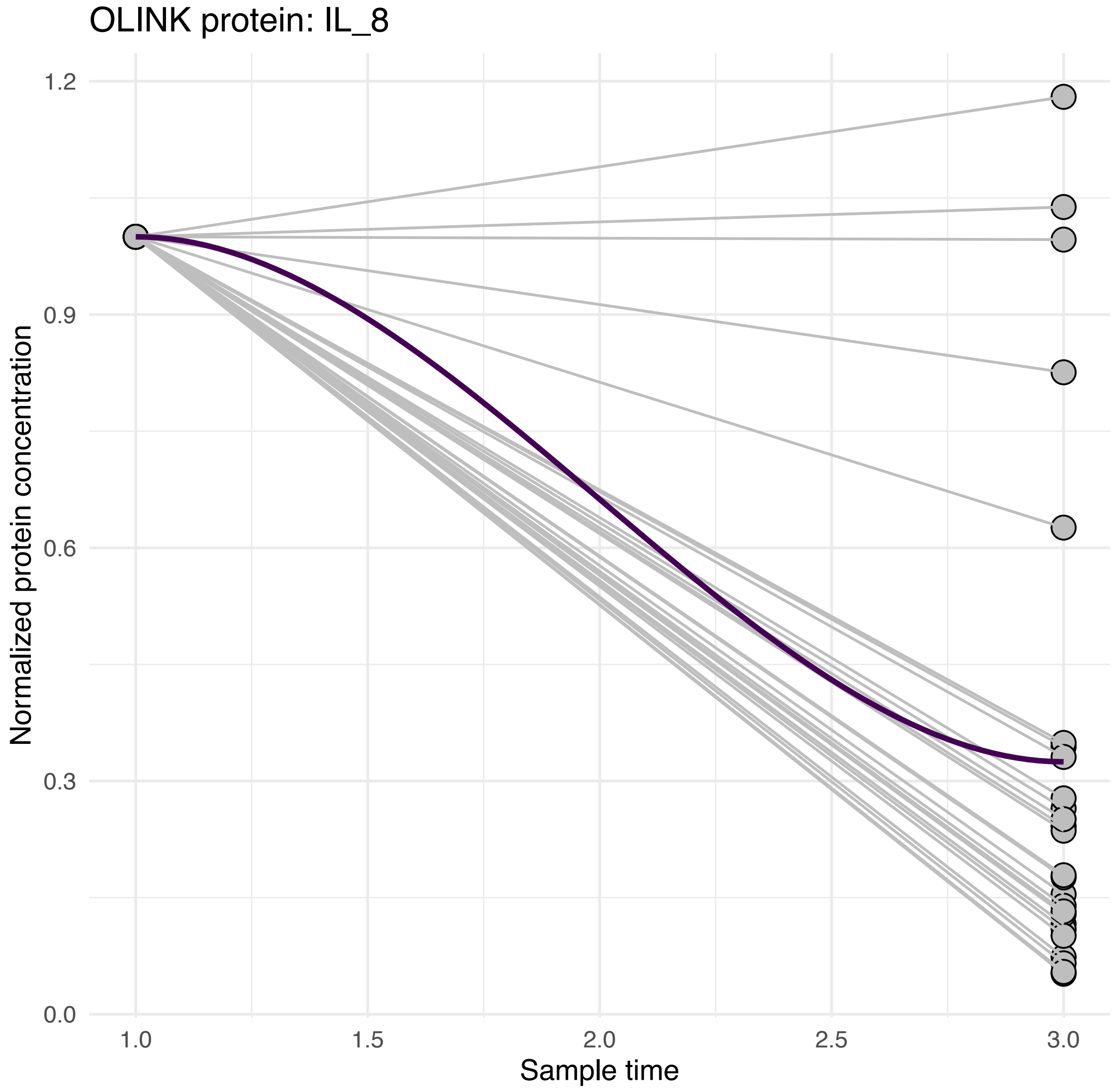
OLINK protein: IL_8
Normalized protein concentration
1.2
0.9
0.6
0.3
0.0
Sample time
1.0
1.5
2.0
2.5
3.0

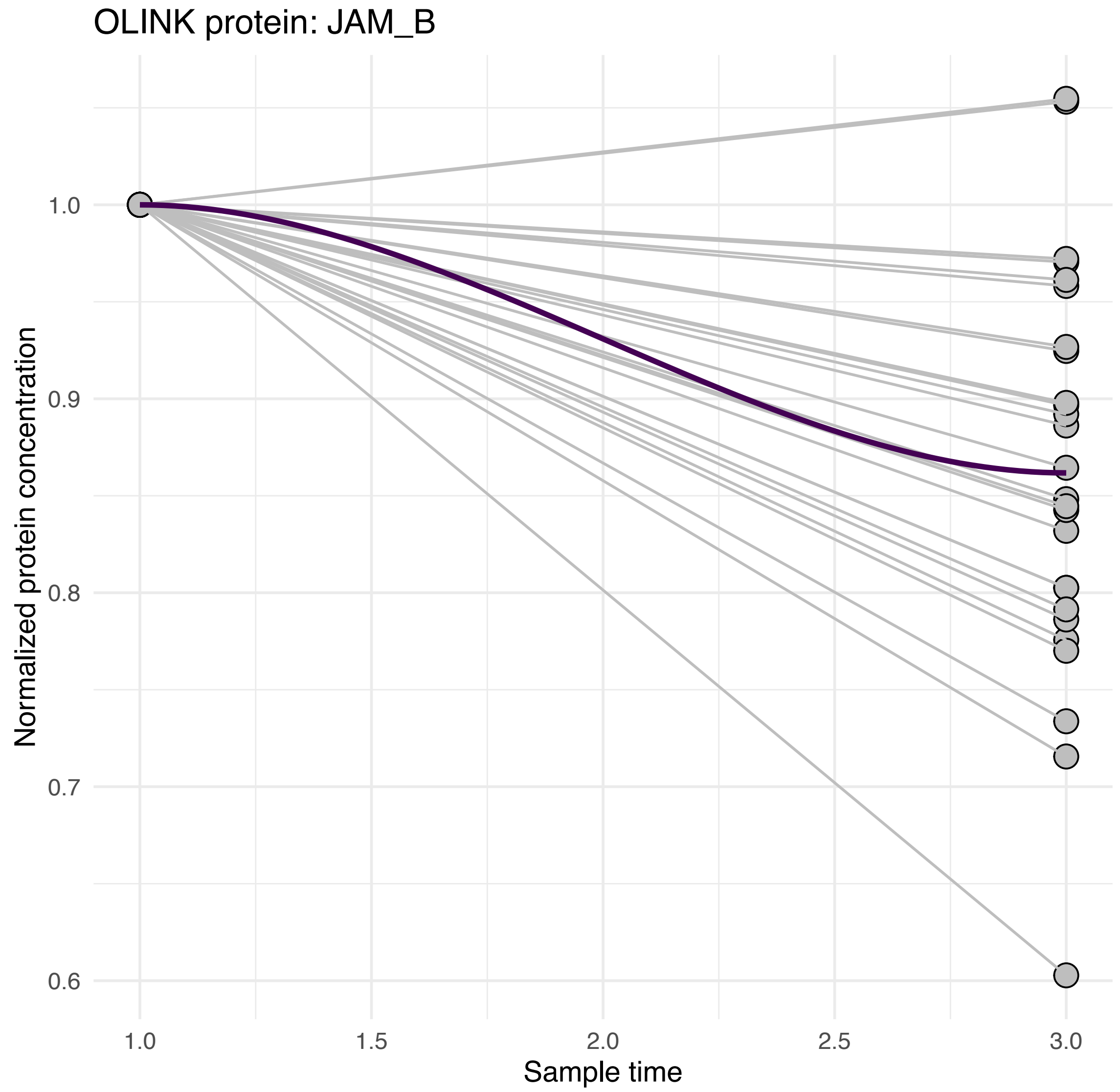
OLINK protein: JAM_B
Normalized protein concentration
1.0
0.9
0.8
0.7
0.6
1.0 1.5 2.0 2.5 3.0
Sample time

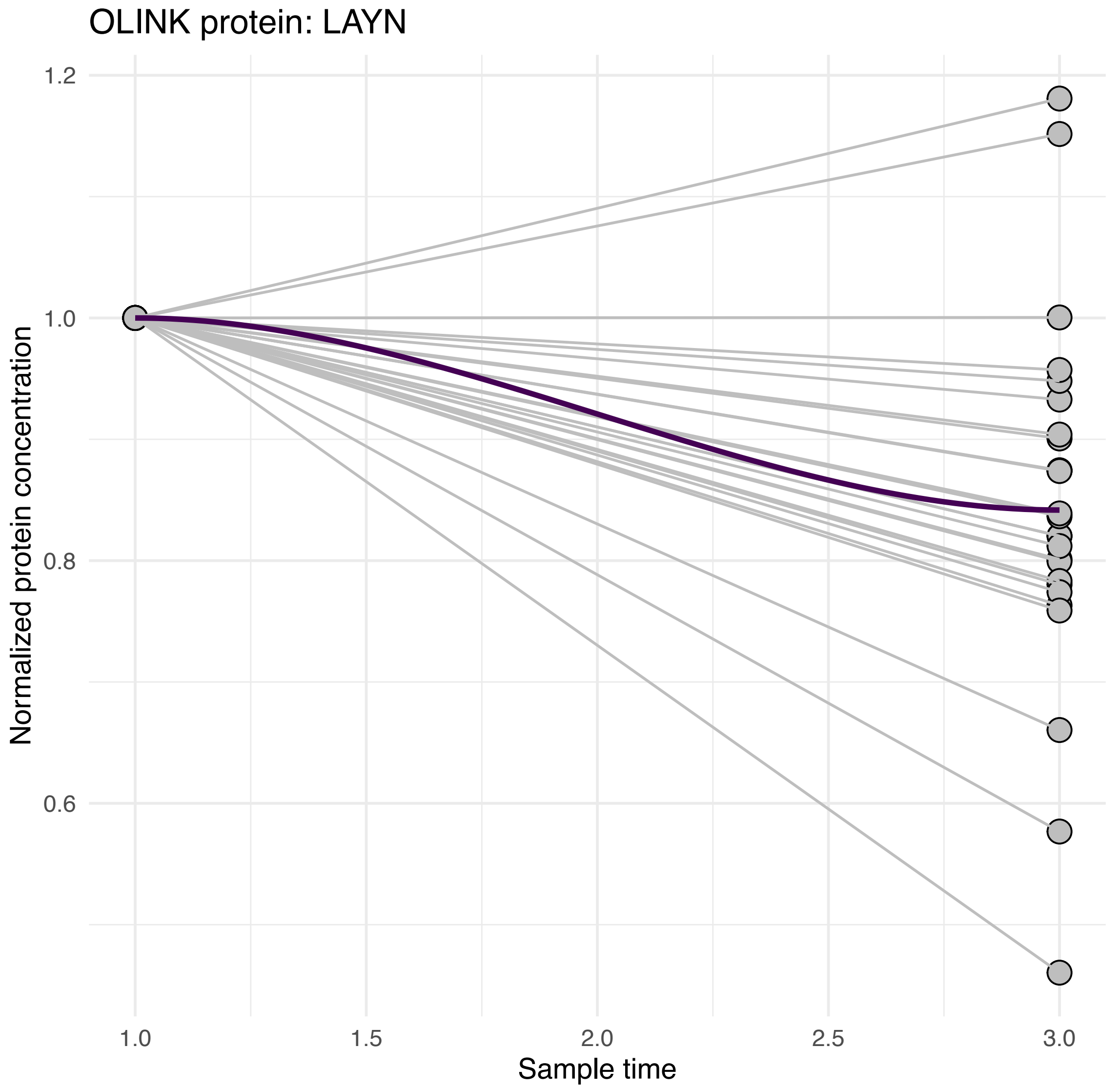
OLINK protein: LAYN
Normalized protein concentration
1.2
1.0
0.8
0.6
1.0 1.5 2.0 2.5 3.0
Sample time

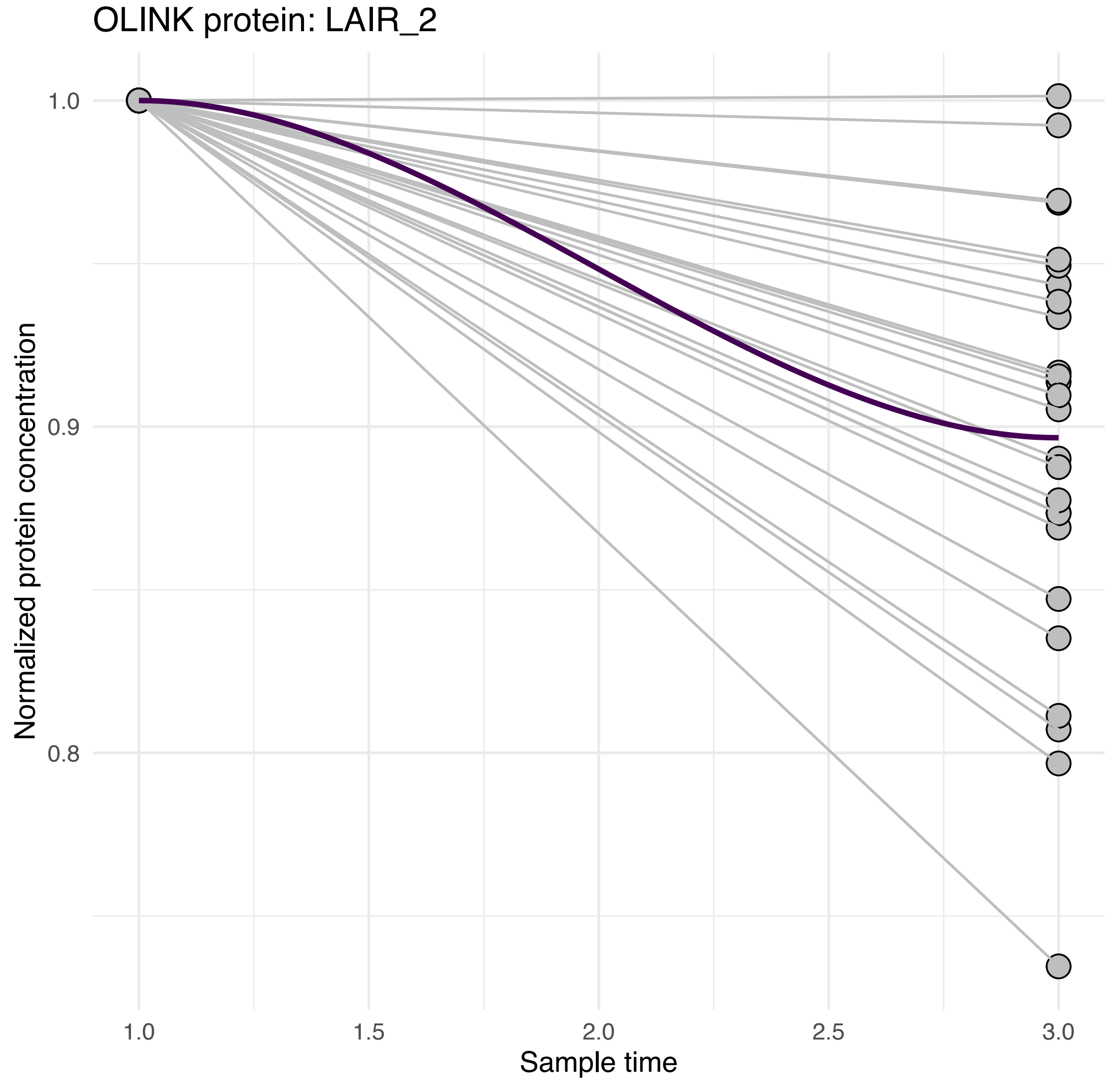
OLINK protein: LAIR_2
Normalized protein concentration
1.0
0.9
0.8
1.0 1.5 2.0 2.5 3.0
Sample time

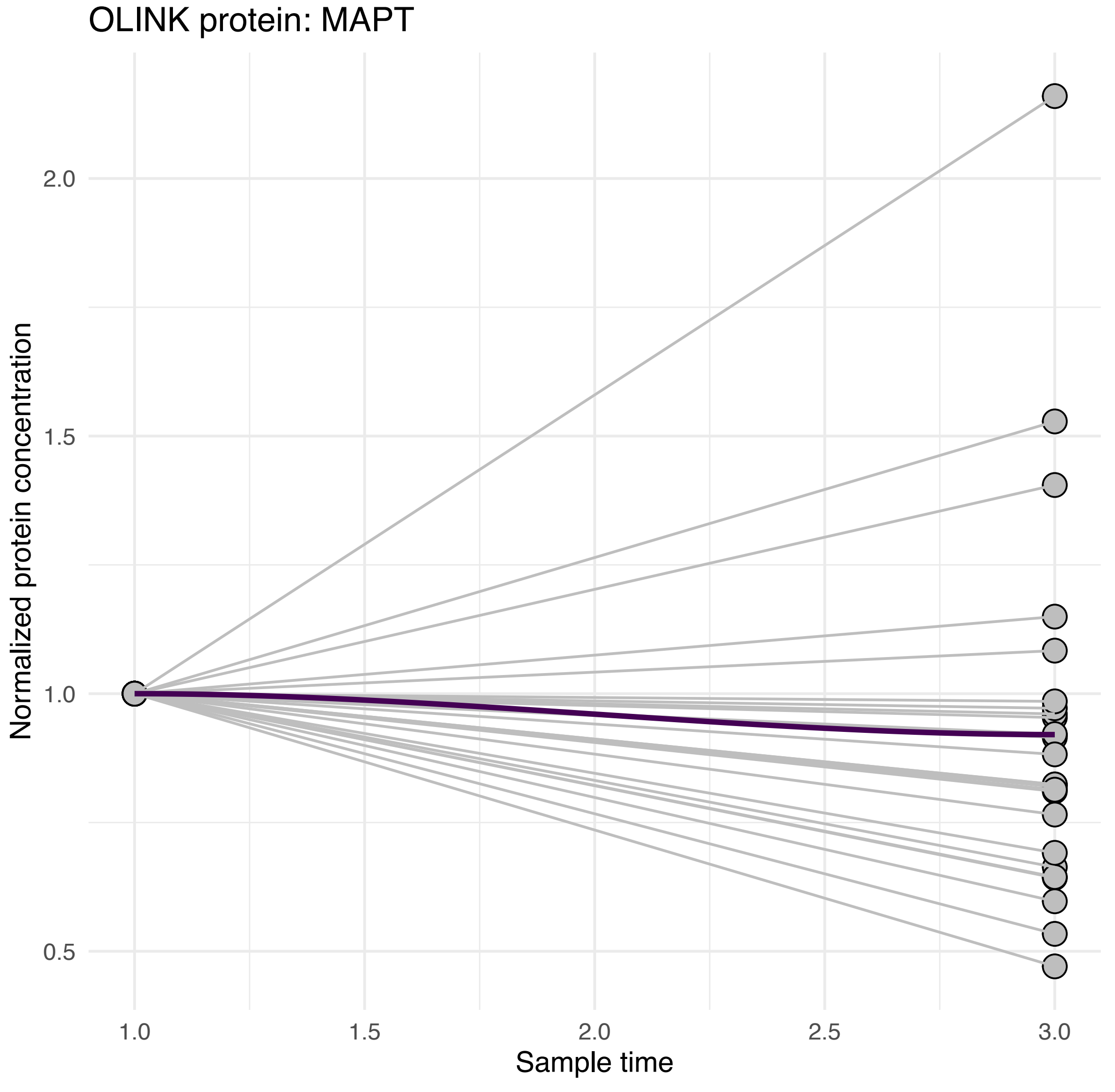
OLINK protein: MAPT
Normalized protein concentration
2.0
1.5
1.0
0.5
1.0 1.5 2.0 2.5 3.0
Sample time

OLINK protein: MATN3

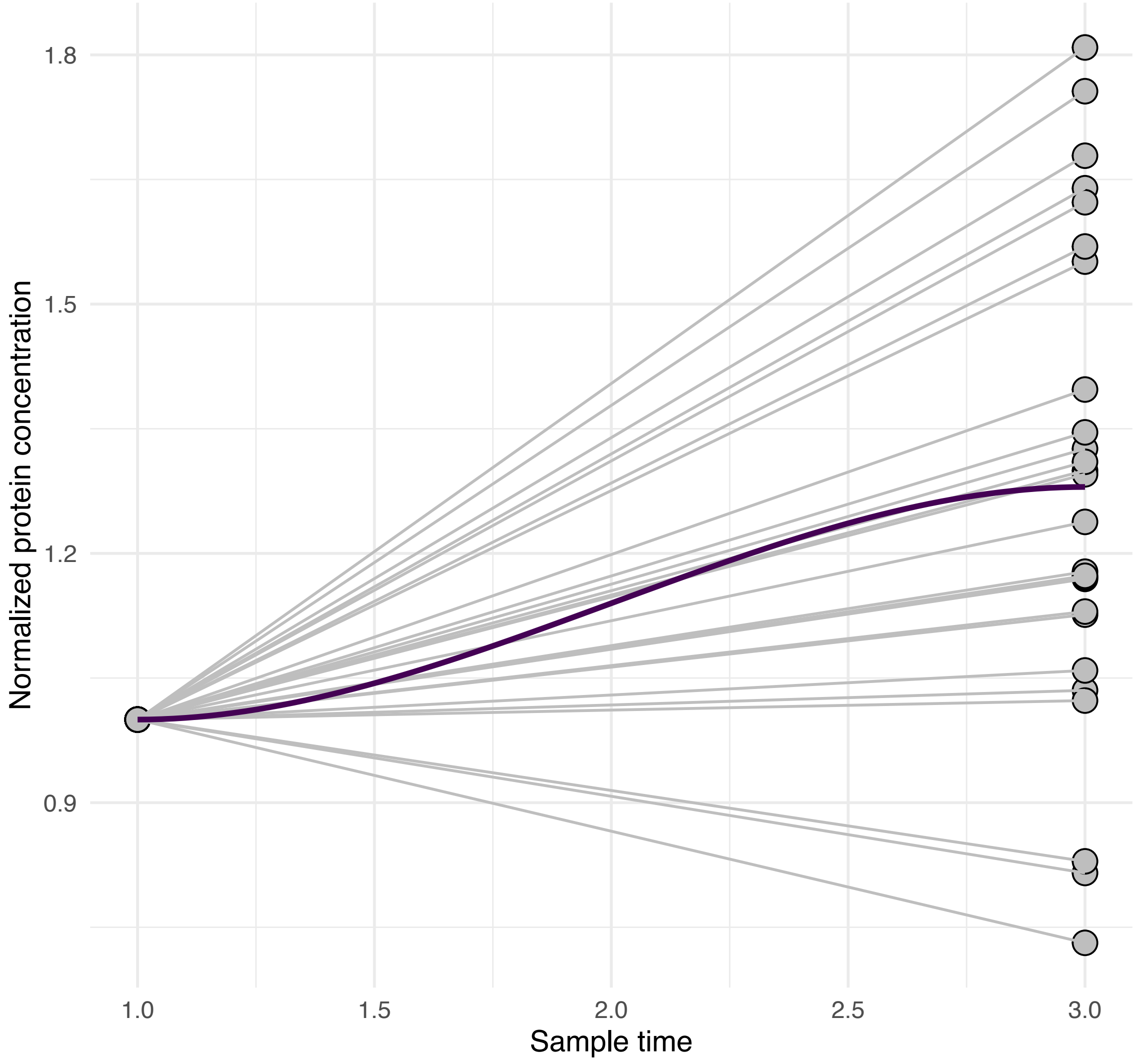

OLINK protein: NBL1

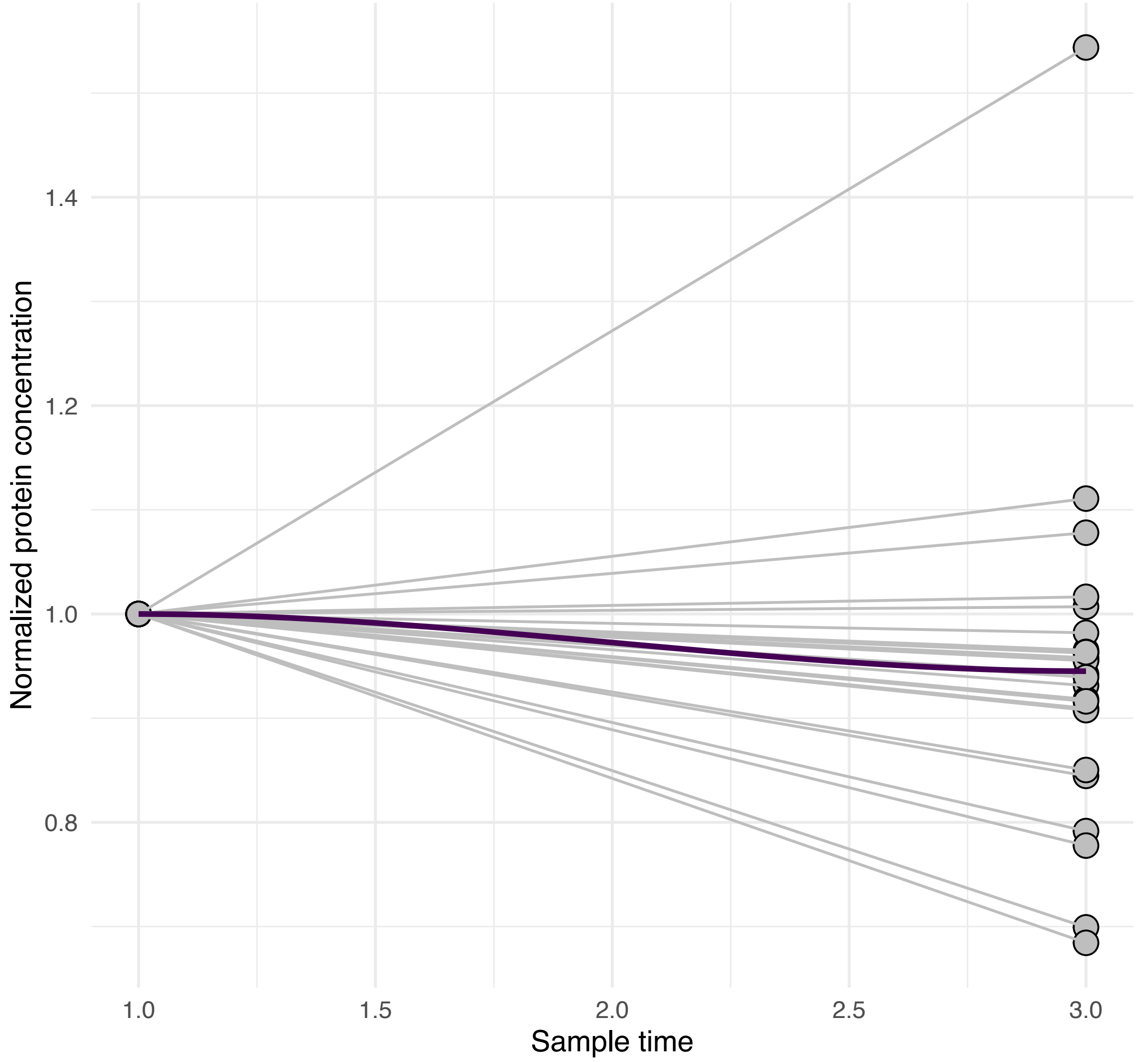

OLINK protein: MSR1

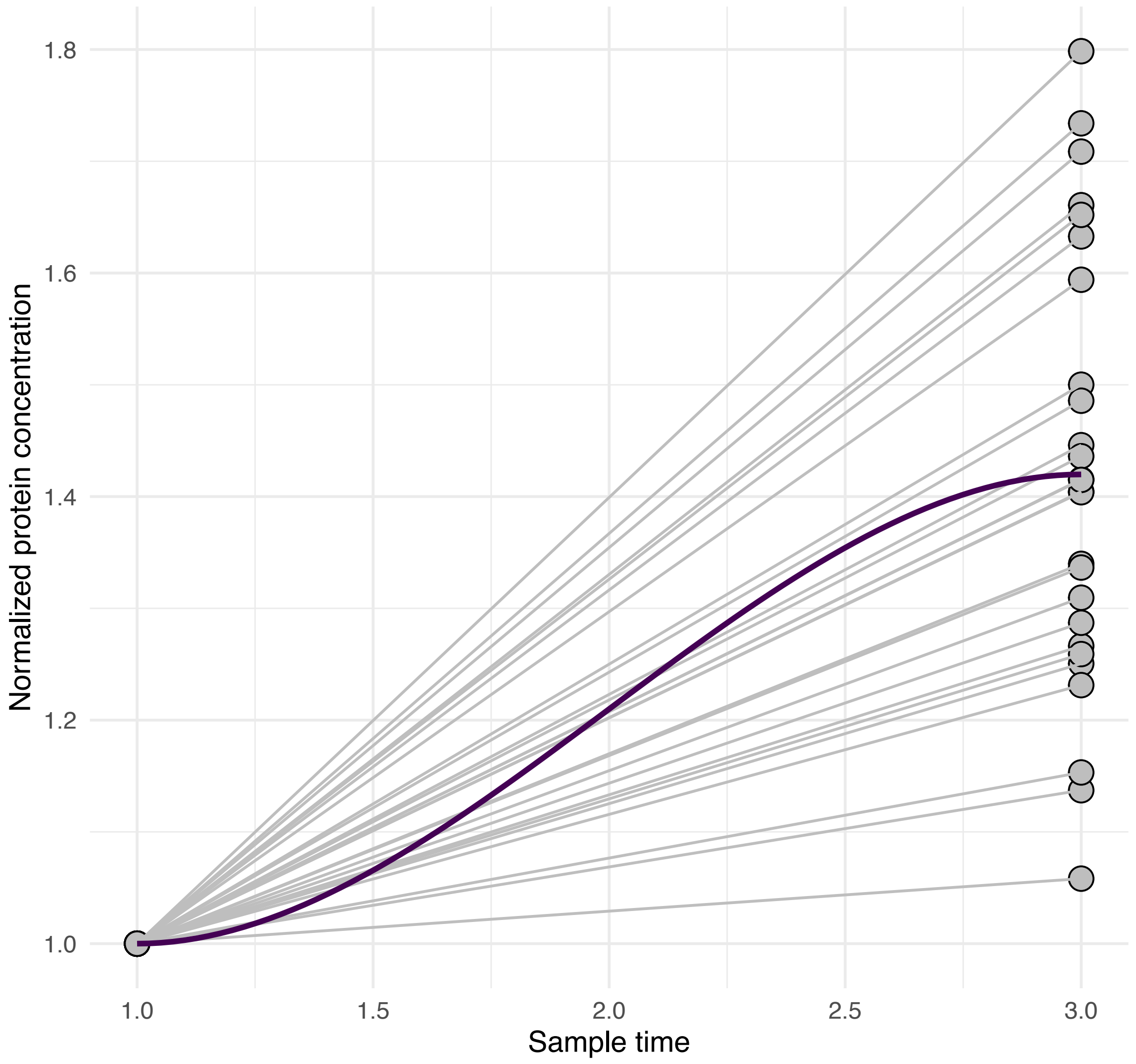

OLINK protein: NCAN

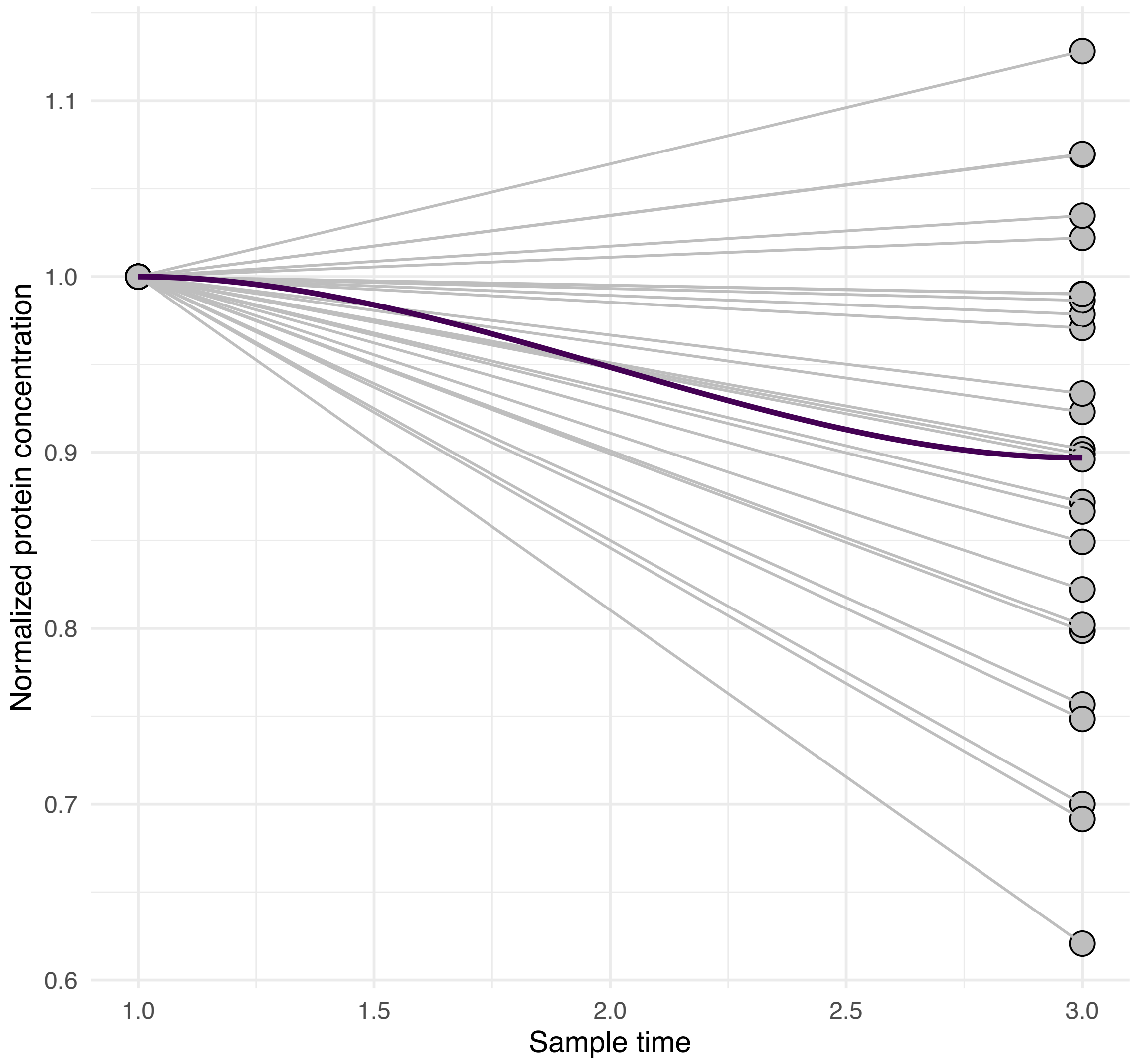

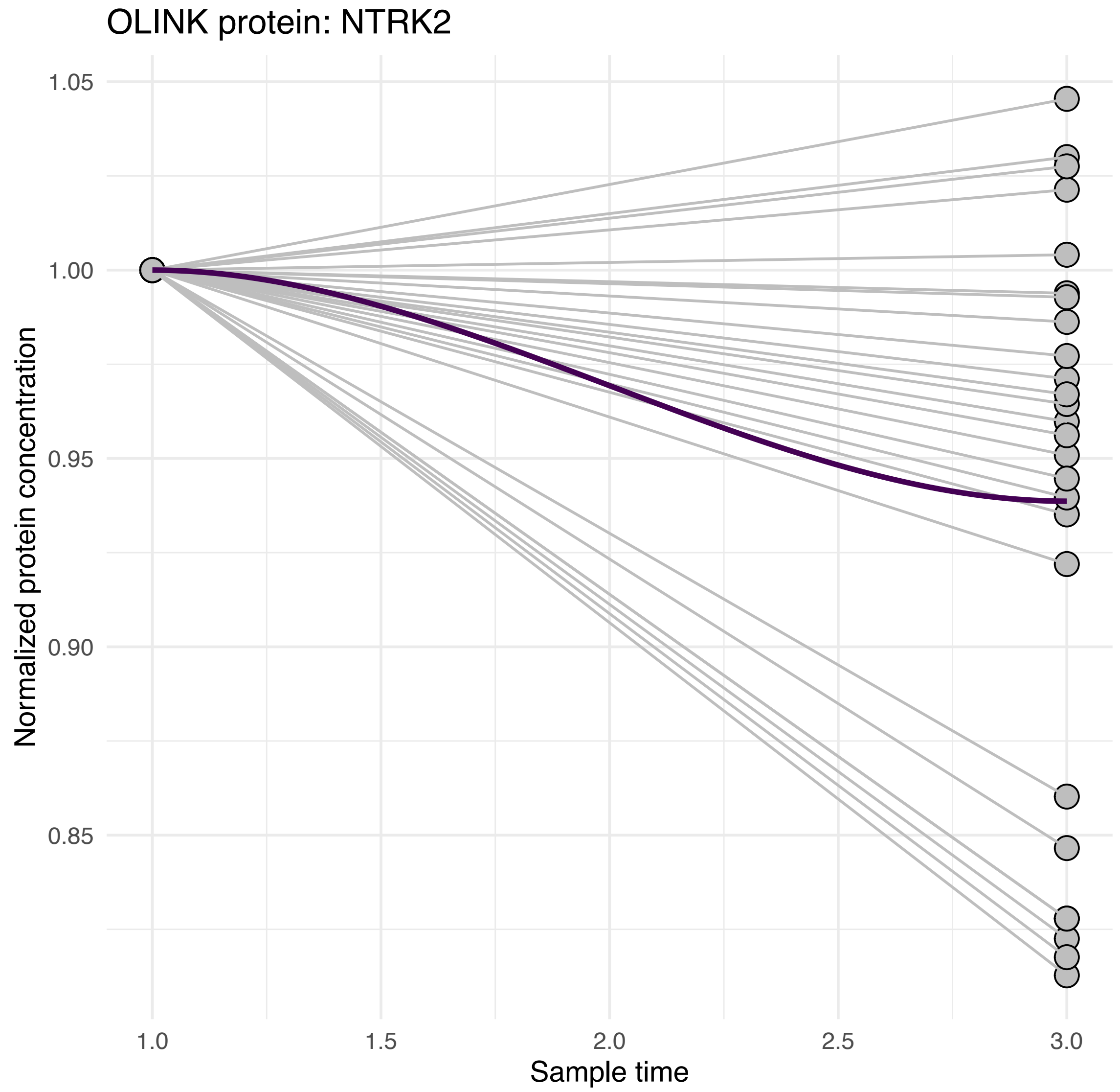
OLINK protein: NTRK2
Normalized protein concentration
1.05
1.00
0.95
0.90
0.85
1.0
1.5
2.0
2.5
3.0
Sample time

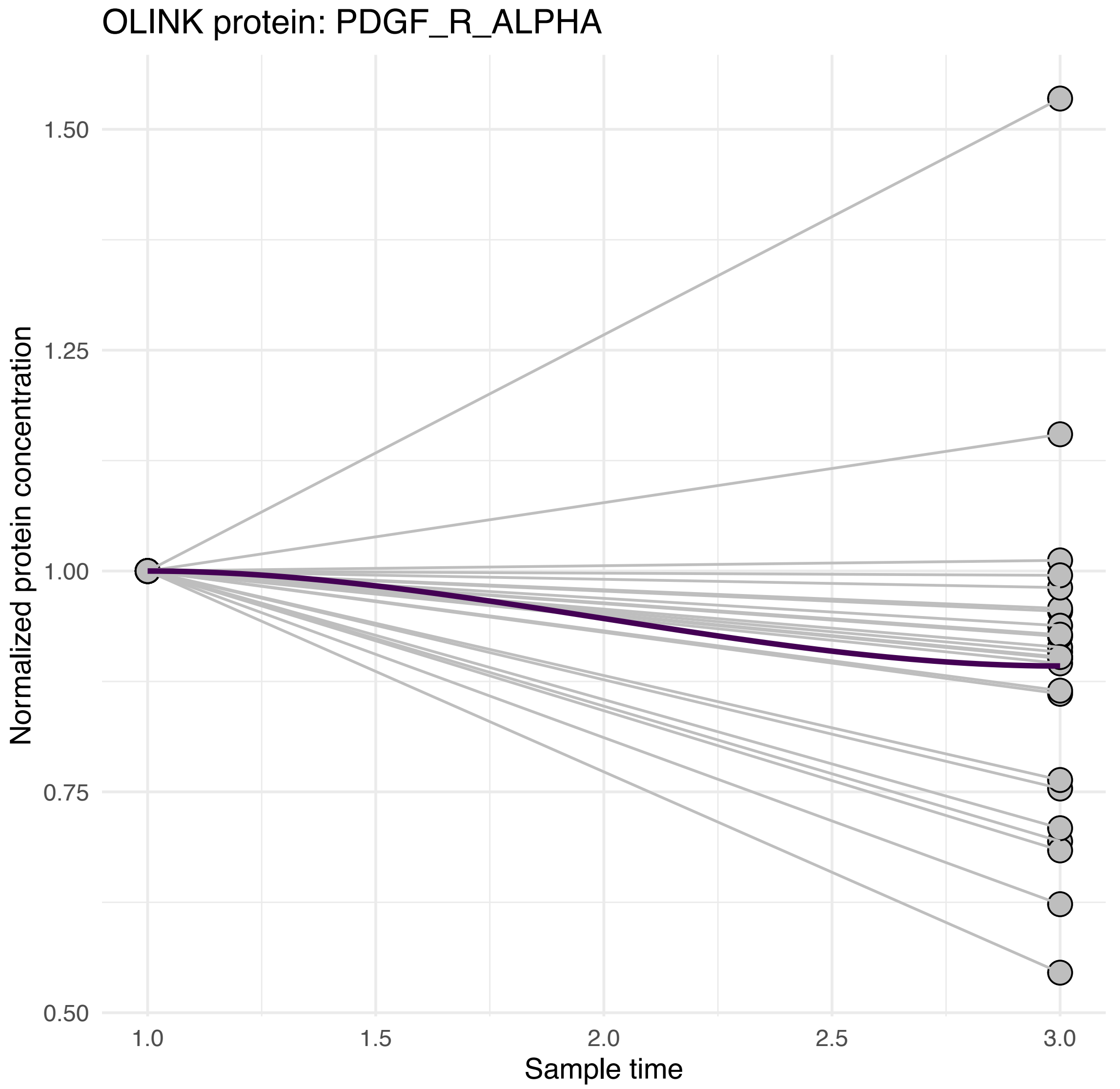
OLINK protein: PDGF_R_ALPHA
Normalized protein concentration
1.50
1.25
1.00
0.75
0.50
1.0
1.5
2.0
2.5
3.0
Sample time

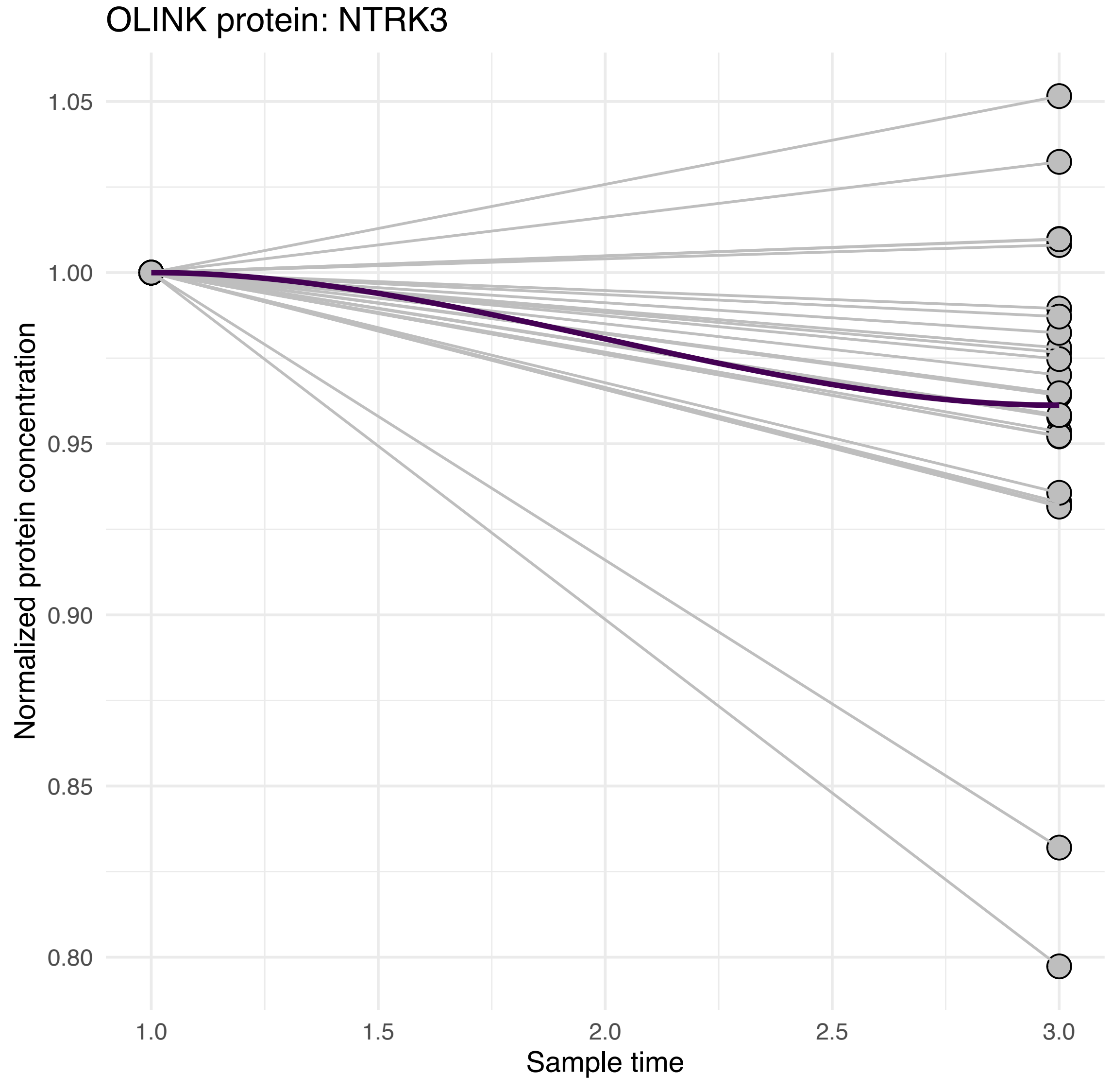
OLINK protein: NTRK3
Normalized protein concentration
1.05
1.00
0.95
0.90
0.85
0.80
1.0
1.5
2.0
2.5
3.0
Sample time

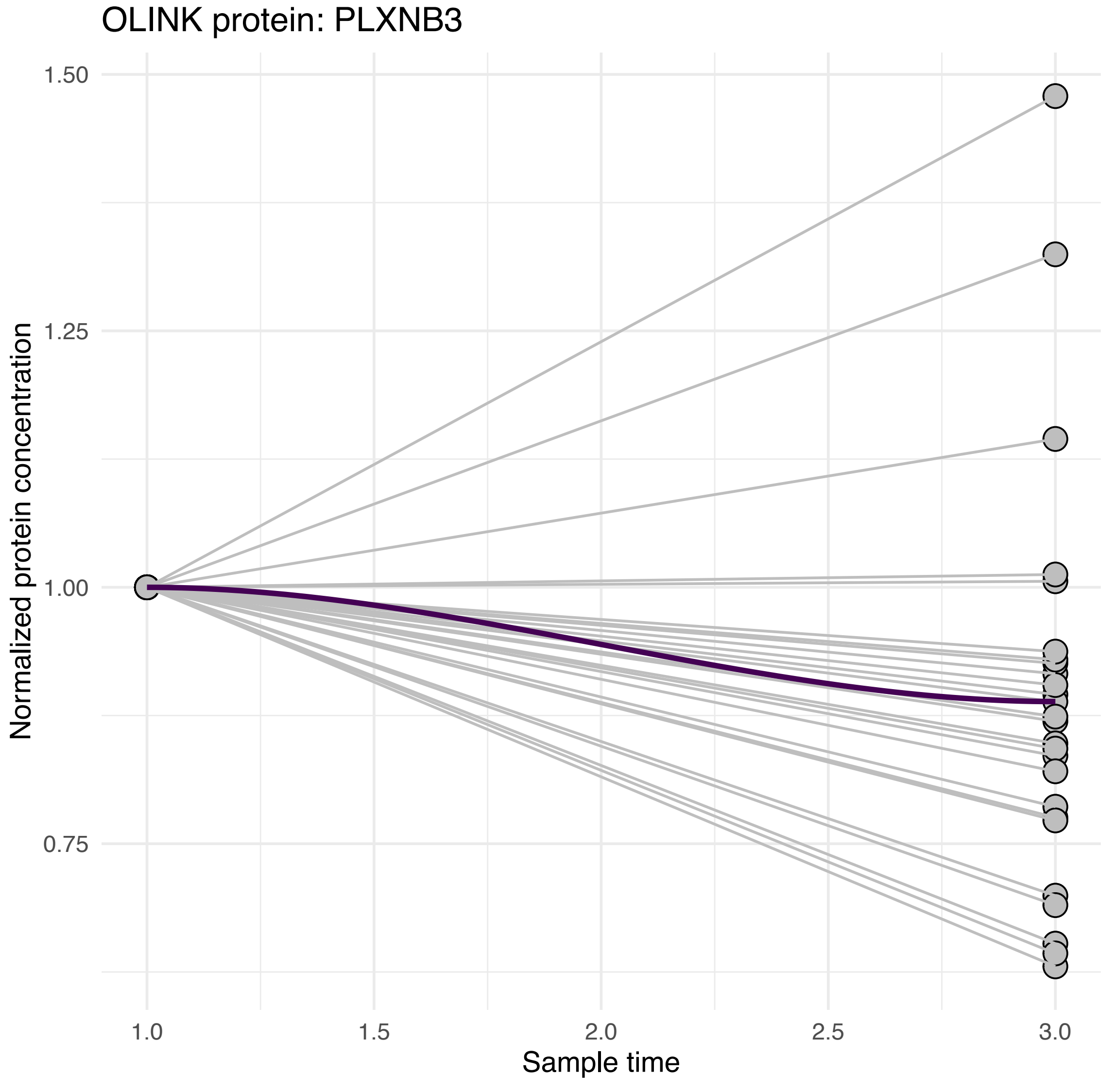
OLINK protein: PLXNB3
Normalized protein concentration
1.50
1.25
1.00
0.75
1.0
1.5
2.0
2.5
3.0
Sample time

OLINK protein: RGMA

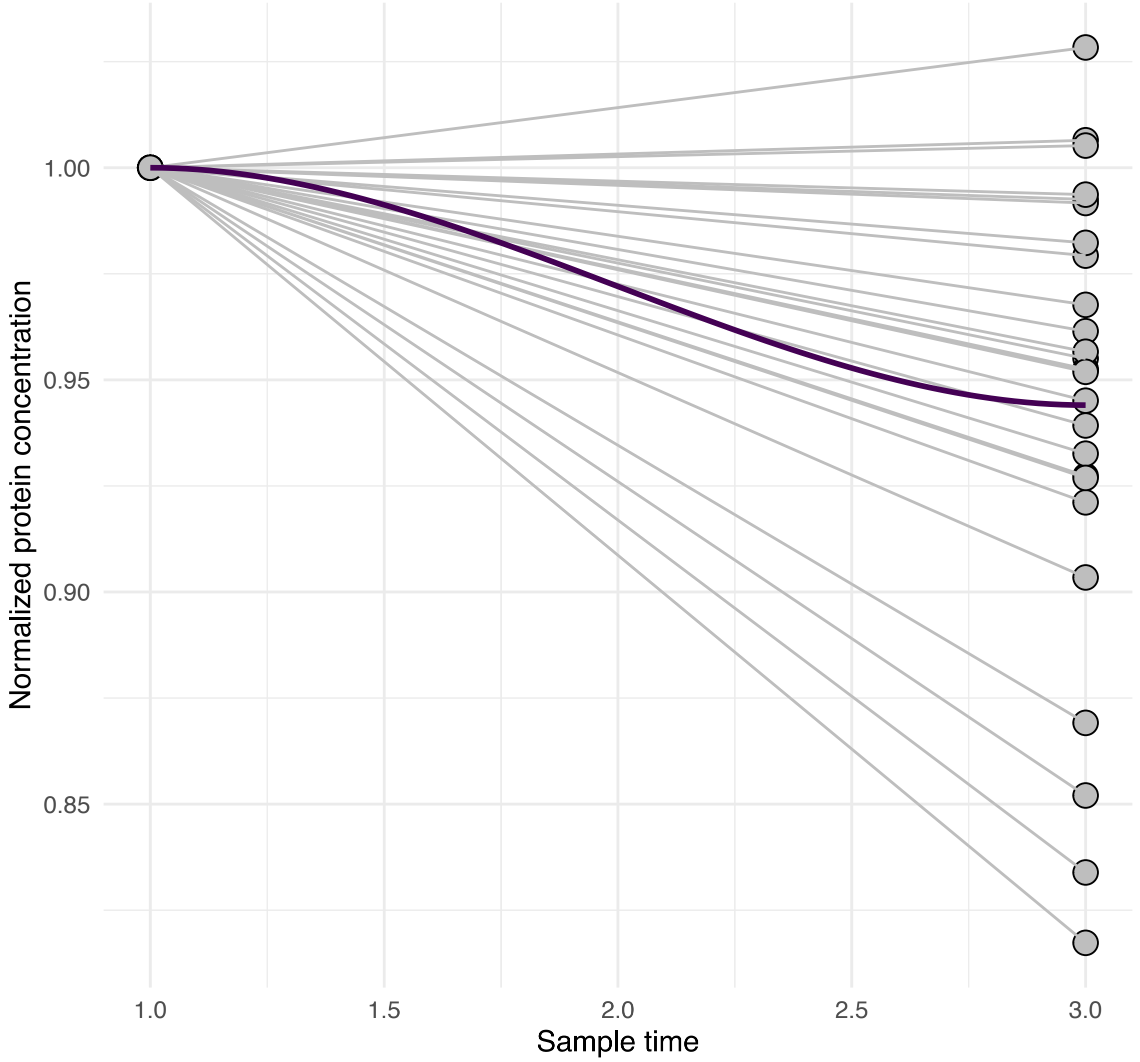

OLINK protein: SIGLEC_9

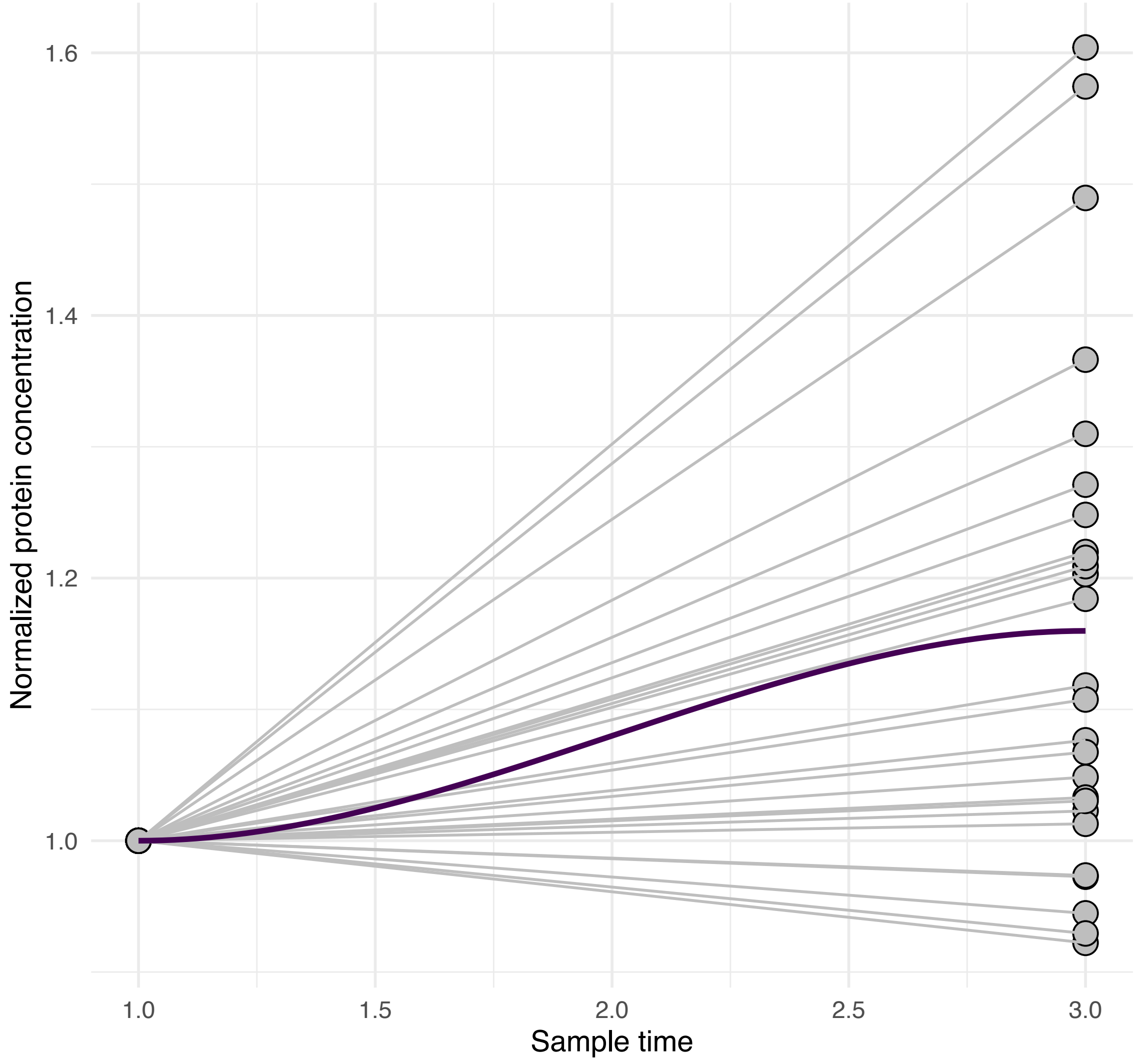

OLINK protein: RGMB

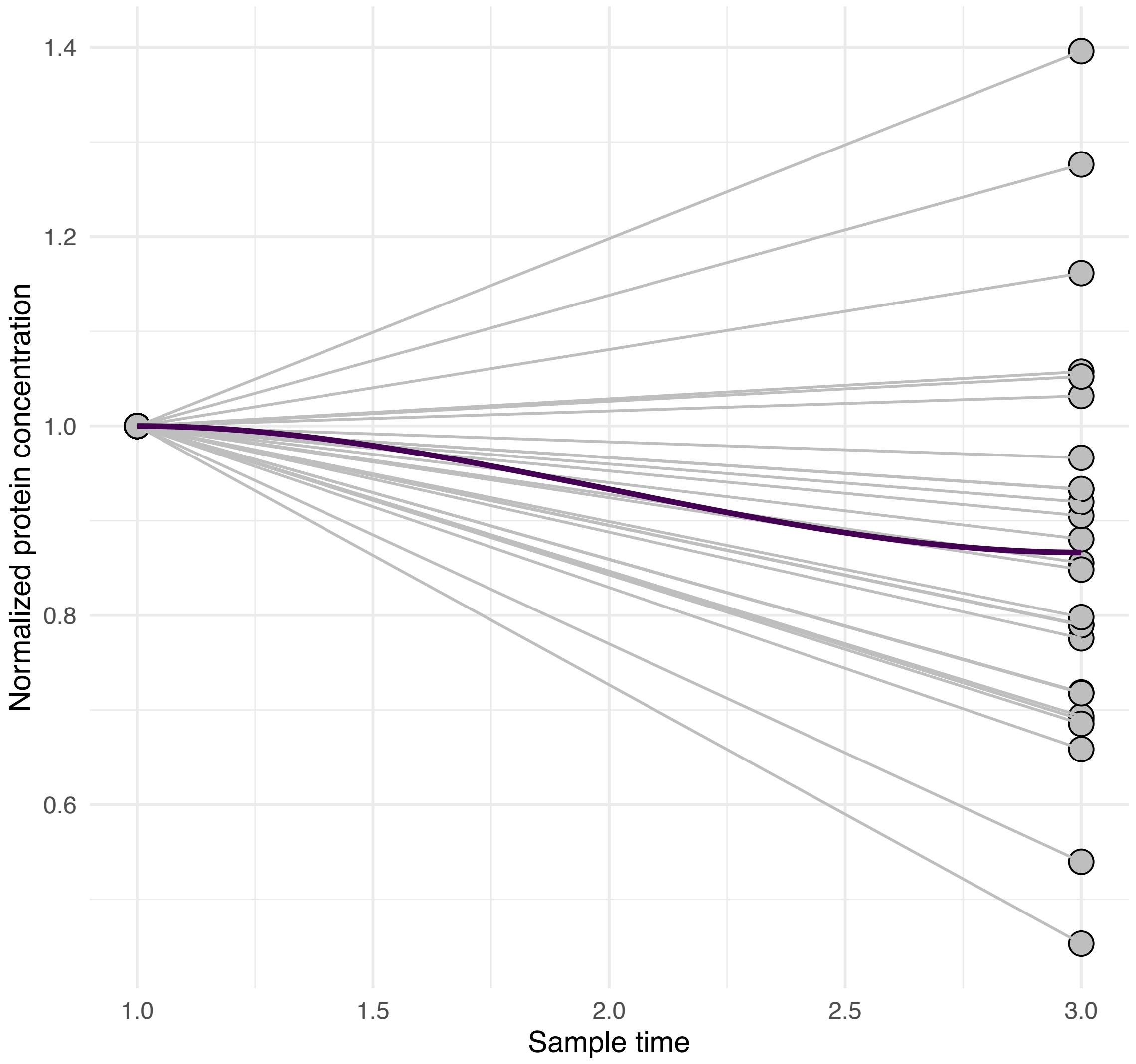

OLINK protein: SKR3

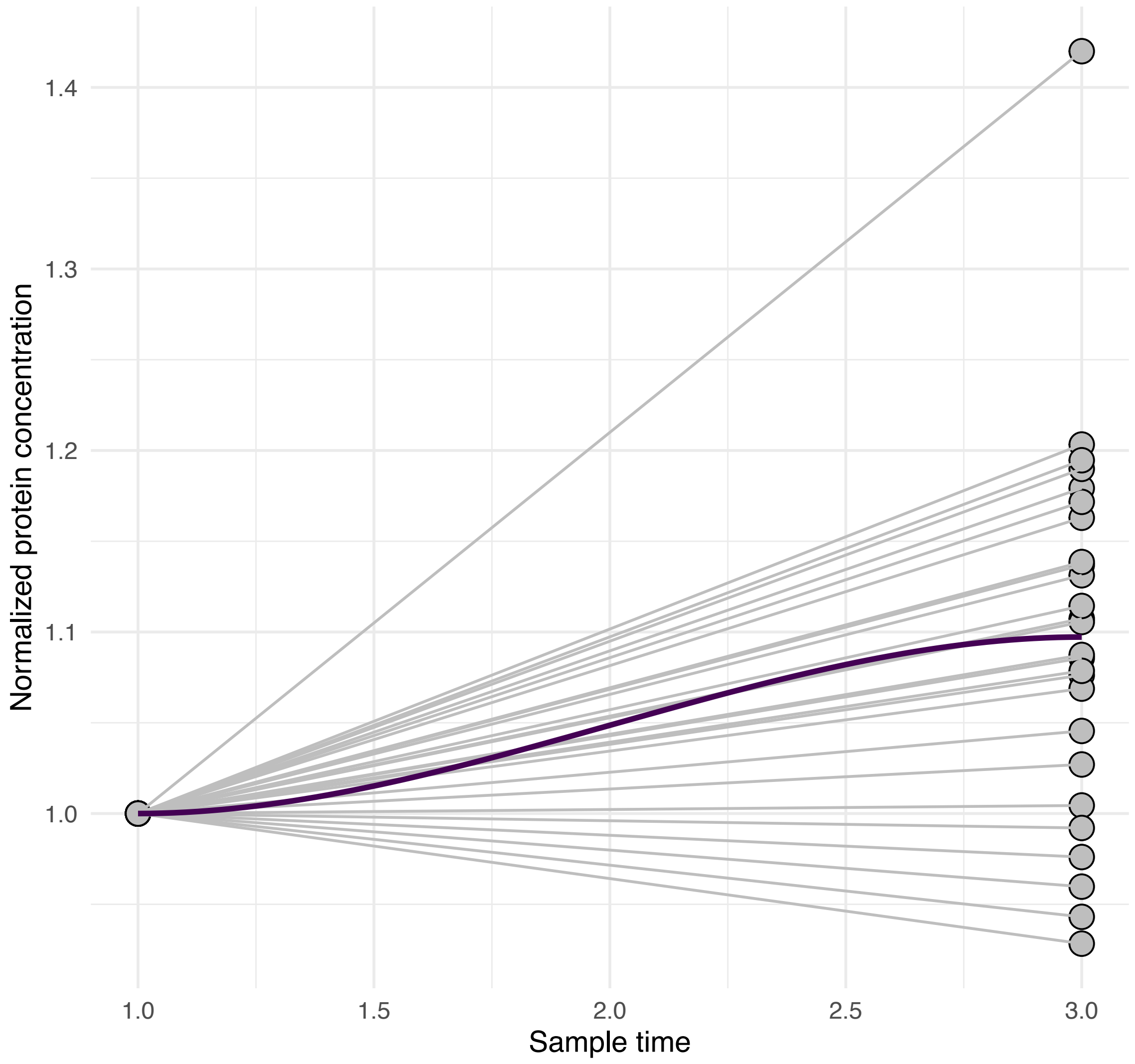

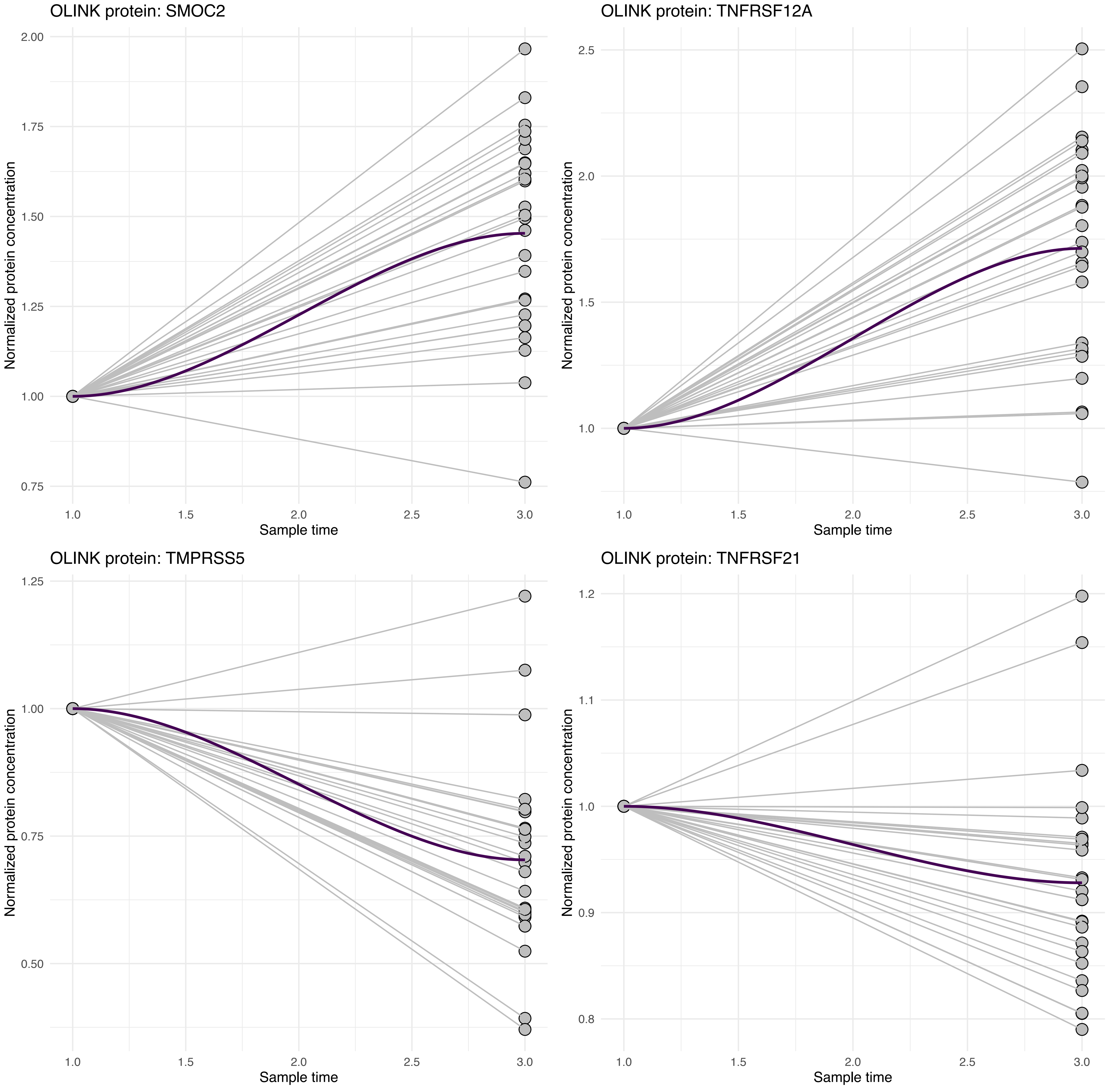
OLINK protein: SMOC2
OLINK protein: TNFRSF12A
OLINK protein: TMPRSS5
OLINK protein: TNFRSF21
Normalized protein concentration
Sample time
2.00
1.75
1.50
1.25
1.00
0.75
2.5
2.0
1.5
1.0
1.25
1.00
0.75
0.50
1.2
1.1
1.0
0.9
0.8
1.0
1.5
2.0
2.5
3.0

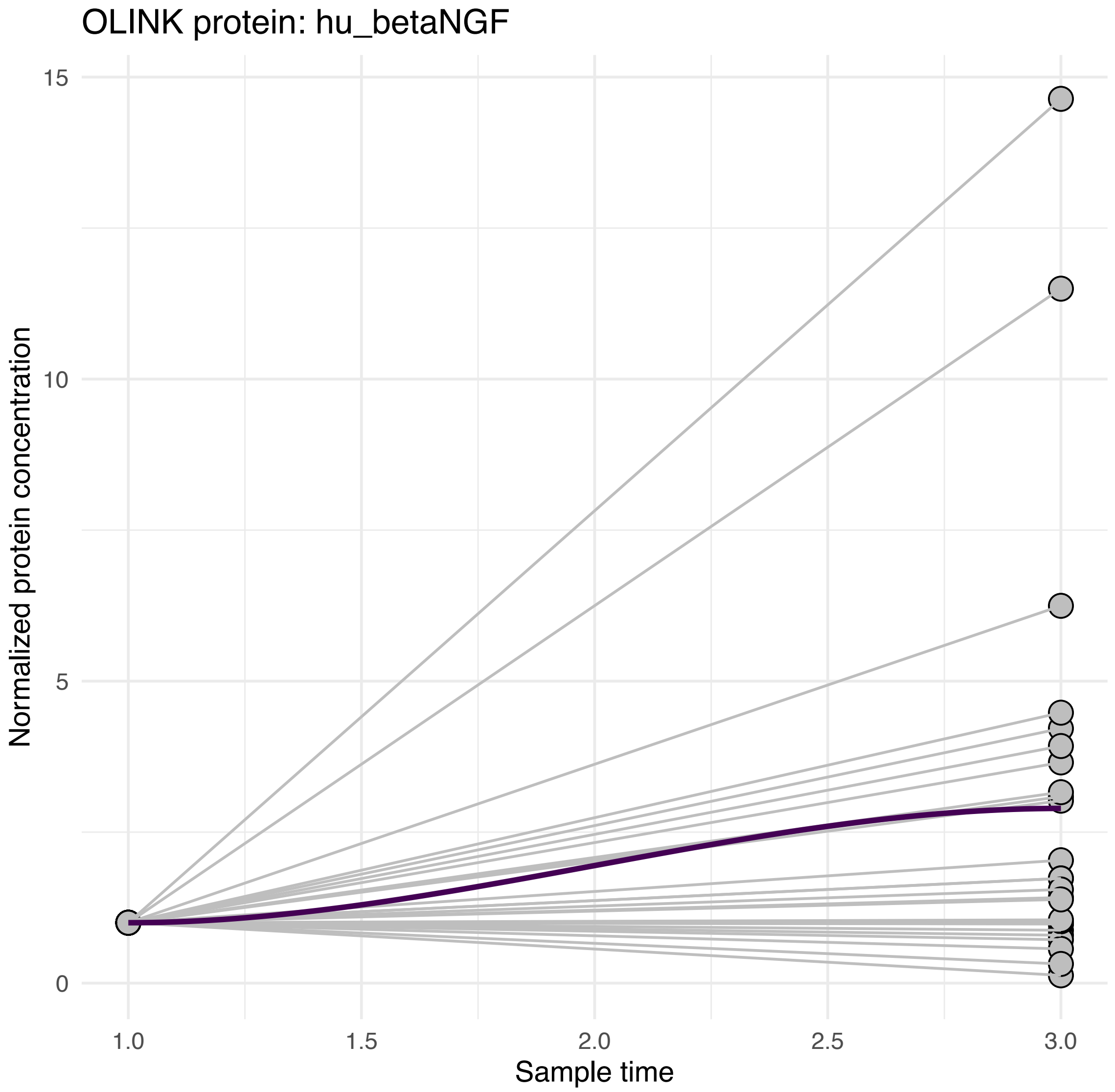
OLINK protein: hu_betaNGF
Normalized protein concentration
Sample time
0
5
10
15
1.0
1.5
2.0
2.5
3.0
